# Tuning DO:DM Ratios Modulates MHC Class II Immunopeptidomes

**DOI:** 10.1016/j.mcpro.2022.100204

**Published:** 2022-01-25

**Authors:** Niclas Olsson, Wei Jiang, Lital N. Adler, Elizabeth D. Mellins, Joshua E. Elias

**Affiliations:** 1 Department of Chemical and Systems Biology, Stanford School of Medicine, Stanford University, Stanford, California, USA; 2 Department of Pediatrics – Human Gene Therapy, Stanford University School of Medicine, Stanford University, Stanford, California, USA; 3 Stanford Immunology, Stanford University School of Medicine, Stanford, California, USA; 4 Chan Zuckerberg Biohub, Stanford, California, USA

**Keywords:** antigen presentation, HLA, DO, DM, MHC, mass spectrometry, immunopeptidome, proteome

## Abstract

Major histocompatibility complex class II (MHC-II) antigen presentation underlies a wide range of immune responses in health and disease. However, how MHC-II antigen presentation is regulated by the peptide-loading catalyst HLA-DM (DM), its associated modulator, HLA-DO (DO), is incompletely understood. This is due largely to technical limitations: model antigen-presenting cell (APC) systems that express these MHC-II peptidome regulators at physiologically variable levels have not been described. Likewise, computational prediction tools that account for DO and DM activities are not presently available. To address these gaps, we created a panel of single MHC-II allele, HLA-DR4-expressing APC lines that cover a wide range of DO:DM ratio states. Using a combined immunopeptidomic and proteomic discovery strategy, we measured the effects DO:DM ratios have on peptide presentation by surveying over 10,000 unique DR4-presented peptides. The resulting data provide insight into peptide characteristics that influence their presentation with increasing DO:DM ratios. These include DM sensitivity, peptide abundance, binding affinity and motif, peptide length, and choice of binding register along the source protein. These findings have implications for designing improved HLA-II prediction algorithms and research strategies for dissecting the variety of functions that different APCs serve in the body.

## Highlights

• A new cell line panel with varied DO:DM ratios informs MHC-II presentation.
• Tuning DO:DM ratios corresponds with strikingly divergent MHC-II immunopeptidomes.
• Our study bridges a gap in understanding and predicting MHC-II antigen presentation.

## Graphical Abstract

**Figure:**
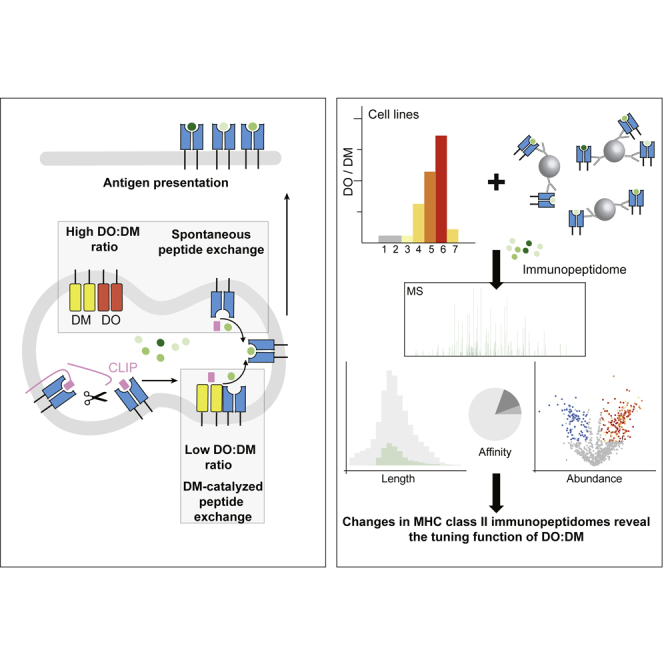

## In Brief

Immunopeptide presentation by MHC-II regulates adaptive immunity. The noncanonical MHC molecules HLA-DM and HLA-DO cooperatively regulate MHC-II function, and their relative abundances vary across APCs and cellular contexts. How this variation influences immunopeptide repertoires remains unclear. We addressed this by creating cell lines expressing HLA-DM and HLA-DO, spanning several relative abundances and measuring their immunopeptide repertoires. We found that immunopeptides clustered according to their presentation levels across different DO:DM ratios. Predicted MHC-II binding affinity substantially contributed to but did not fully account for these results.

The immune system develops effector T cell repertoires that are both tolerant of self-proteins and reactive to foreign antigens through selection steps that remove T cell clones bearing high-affinity, self-reactive antigen receptors (1, 2). Professional antigen-presenting cells (APCs) present peptides in complex with major histocompatibility complex class II (pMHC-II), in a process that is essential to CD4+ T cell development and clonal selection. During self-tolerance acquisition in the thymus, medullary thymic epithelia and other APCs present self-pMHC-II where they are encountered by developing CD4+ T cells, driving their maturation and selection (1). During antigen exposure in the periphery, diverse pMHC-II presented by APCs, like dendritic cells and B cells in the germinal centers (GCs) of lymph nodes, expand antigen-reactive mature T cell clones (2). Some APCs express a nonclassical MHC-II molecule, DO (HLA-DO in human or H2-O in mouse) (3, 4, 5, 6, 7). These include medullary thymic epithelia, B cells, and certain CD141+ dendritic cell subsets, but not macrophages (8, 9, 10). This restricted expression pattern implies a unique yet critical role for DO in regulating pMHC-II presentation and subsequent CD4+ T-dependent immune responses.

Consistent with DO-regulated immune responses, ectopic overexpression of H2-O in H2-O^neg^ dendritic cells diminished the presentation of certain pMHC-II (11) and prevented diabetogenic T cell activation and subsequent type 1 diabetes in NOD mice (12). Conversely, H2-O knockout mice spontaneously developed increased T-dependent autoantibody titers but showed delayed humoral immunity when immunized with a model antigen (13). These mice also exhibited increased susceptibility to model autoimmune diseases (14). In contrast, other researchers found that H2-O deficiency promoted virus-neutralizing antibody production (15). These experiments support the idea that the immunologic consequences of DO’s effects on pMHC-II repertoires are highly context dependent such that DO knockout animals can exhibit autoreactive or antimicrobial responses compared with DO-sufficient animals. Physiologically, DO expression is downregulated in naïve or memory B cells entering GC to recruit T cell help (5, 6, 16). DO downregulation also occurs in certain dendritic cells following their exposure to maturation stimuli (8, 9). Thus, changes in DO levels influence a wide range of adaptive immune responses.

Recently, we proposed a mechanistic model of how DO regulates MHC-II peptide presentation, considering both molecular and cellular assessments of DO function (17). DO functions through its pH-dependent association with DM (18, 19, 20, 21), a peptide exchange catalyst that selects for stable pMHC-II (22, 23), although not all high-affinity peptides are resistant to DM. The DM selection process is termed editing. We also found that the presented peptidome can be highly sensitive to DO regulation, as small changes in DO:DM stoichiometry (the DO-to-DM ratio is denoted as DO:DM) can create substantial shifts in pMHC-II levels (17). As noted earlier, several studies have shown that DO knockout or ectopic expression in murine or human cells influences certain pMHC-II or the entire MHC-II peptidome (11, 14, 24, 25, 26, 27, 28, 29, 30). However, no study has yet measured how pMHC-II repertoires change between graded DO:DM differences, such as those that exist among APC types or those that progressively occur upon APC activation (8, 17).

Here, we performed a detailed mass spectrometric (MS) analysis on the peptidomes associated with an MHC-II human leucocyte antigen allele, HLA-DR4, expressed by model antigen-presenting cell lines, by jointly considering the related proteomic information. These cell lines cover a wider range of DO:DM stoichiometry than the previously used DO+ *versus* DO- states. Analyzing these cell lines with MS enabled us to empirically define distinct HLA-II peptidome subsets based on their apparent sensitivity to DM activity and the corresponding tuning property of DO:DM. We also described a knockdown example that illustrates how DO downregulation would affect such tuning of the peptidome. Using these MS analyses, we found that DO:DM tuning has a graded effect on different pMHC-II subsets and investigated several peptide characteristics that could be associated with DO:DM-tuned pMHC-II presentation. Our findings will advance research in DMDO-regulated antigen presentation, improve computational prediction models of MHC-II antigen presentation, and suggest strategies in the management of T-dependent immune disorders.

## Experimental Procedures

### Cell Line Construction

The previously described cell lines included in this work are T2 (MHC-II^null^DO^null^DM^null^ T x B hybrid), T2DR4 (DO^null^DM^null^), T2DR4DM, and two clonal cell lines, T2DR4DMDO++ (1C3) and T2DR4DMDO+++ (2D7) (17, 21). Cell lines with graded levels of DO:DM were constructed as follows. A T2DR4DMDO parent line was constructed by transfecting the pBudCE4.1-DOA/DOB plasmid into T2DR4DM by nucleofection (21). The T2DR4DMDO++ (medium level of DO:DM, or (DO:DM)^M^) and T2DR4DMDO+++ (high level of DO:DM, or (DO:DM)^H^) clonal lines were constructed by fluorescence-activated cell sorting (FACS) of the parental, polyclonal T2DR4DMDO transfectant for single cells with low (*e.g.*, 1C3) or high (*e.g.*, 2D7) levels of surface CLIP/DR4 complexes (17). FACS-sorted single clones were then expanded in a well of a 96-well plate to establish stable, single clonal lines, including 1C3 and 2D7, as described (17). To construct T2DR4DMDO+ (low level of DO:DM, or (DO:DM)^L^), a plasmid pBudCE4.1-DOA/DOB_Gly_8_-linker_FLAG-tag was constructed similarly to the construction of pBudCE4.1-DOA/DOB. After transfection of T2DR4DM with the plasmid by nucleofection, two independent human promoters in the plasmid drove the expression of DOα and DOβ with a C-terminal FLAG-tag, respectively. Amaxa nucleofector kit C (Lonza) designed for nucleofection of T2 cells was used. Transfected cells were cultured in complete Iscove’s modified Dulbecco’s medium (IMDM-GlutaMax, 10% heat-inactivated fetal bovine serum and 1% penicillin–streptomycin) with 50 to 100 μg/ml zeocin for 3 to 5 weeks to eliminate parent nontransfected cells and to construct a stable cell line, T2DR4DMDO-G8. To construct the DOKO cell line, we used the CRISPR gene-editing strategy (31) to knock out *DO* in T2DR4DMDO-2D7 ((DO:DM)^H^) cells. Two single guided RNA (sgRNA) primers, GGCCACCAAGGCTGACCACATGG flanking the *DOA* exon 1/2 and GGGGAGAAAAGTGCAACCAGAGG flanking the *DOB* exon 2/3, were designed using the CRISPOR website (http://crispor.tefor.net/and) and synthesized from Synthego. The *Cas9*-encoding plasmid was a gift from Dr Matthew Porteus at Stanford University. sgRNA and *Cas9* were introduced into T2DR4DMDO-2D7 by nucleofection using Amaxa nucleofector kit C. The nucleofection condition: 10^6^ cells in 100 μl total nucleofection solution with 8 μg of each of the two sgRNA primers and 4 μg of *Cas9*-encoding plasmid. Transfected cells were further scaled up and analyzed for DO expression by flow cytometry as described (17, 21) (see also supplemental Fig. S1 for DO expression levels).

### Cell Culture

All cells were cultured in Iscove’s modified Dulbecco’s medium–GlutaMAX supplemented with 10% heat-inactivated fetal bovine serum and 1% of penicillin–streptomycin. All media, supplements, and selection reagents are purchased from Thermo Fisher Scientific. The expression of DR4, DM, or DO in these T2 transfectants was enforced and maintained by selection with 1 mg/ml G418 (Geneticin), 1 μg/ml puromycin, or 100 μg/ml zeocin, respectively. All cell cultures in this study were maintained in a 37 °C incubator constantly supplied with 5% CO_2_. Cells were harvested by centrifugation for immediate flow cytometric analysis or washed twice with ice-cold PBS and stored at −80 °C for downstream proteomic and peptidomic analyses.

### Cell Line Characterization Using Flow Cytometry

To measure total protein levels by flow cytometry, T2 and its derived cell lines were fixed and permeabilized using the Cytofix/Cytoperm kit (BD Biosciences, Becton, Dickinson and Company). Washed cells were then resuspended in 1× PermWash buffer (BD Biosciences) at a density of 1 million cells per 100 μl and stained on ice with fluorophore-conjugated mAbs. These mAbs included fluorescein isothiocyanate (FITC)-conjugated anti-human CLIP mAb (BD Biosciences), PE (R-phycoerythrin)-conjugated anti-HLA-DR mAb (BD Biosciences), Alexa Fluor 568-conjugated anti-human DO mAb (MagsDO5), Alexa Fluor 647- or Alexa Fluor 700-conjugated anti-human DM mAb (MapDM1). Alexa Fluor 568-MagsDO5, Alexa Fluor 647-MapDM1, and Alexa Fluor 700-MapDM1 were generated previously (17, 21). To stain DR4-associated non-CLIP peptides on the cell surface, cells were pelleted by centrifugation and resuspended in phosphate-buffered saline (PBS) + 1% bovine serum albumin (BSA) at a density of 1 million cells per 100 μl and stained on ice first with hybridoma supernatant containing NFLD.D11, a mouse IgM mAb known to recognize non-CLIP peptide/DR4 complexes (32), and second, with Alexa Fluor 647 goat anti-mouse IgM (Invitrogen, Thermo Fisher Scientific). Labeled cells were washed with corresponding staining buffer (1× PermWash buffer for intracellular staining and PBS + 1% BSA for surface staining) and resuspended in PBS + 1% BSA and analyzed on the BD LSRII flow cytometer at Stanford shared FACS Facility. Flow cytometric data were analyzed using FlowJo software (BD Biosciences).

### Protein Extraction, TMT Labeling, and Hp-RP Fractionation for Proteomic Analysis

Each T2-derived cell line was cultured as described above, from which three aliquots of 1 × 10^7^ cells were collected (three replicate aliquots per T2-derived cell line). To extract total protein contents for MS analysis, each replicate was lysed in 8 M urea, 150 mM NaCl, 5 mM DTT, 50 mM Tris-Cl (pH 8) supplemented with Complete Protease Inhibitor Cocktail tablet (Roche) and 1× Halt Protease and Phosphatase Inhibitor Cocktail (Thermo Fisher Scientific). The lysate was then centrifuged at 13,200 rpm for 15 min, and the supernatant was transferred to a fresh test tube for a second round of centrifugation. The resulting clarified supernatant was reduced with 5 mM DTT for 30 min at 37 °C, then alkylated with 14 mM iodoacetamide for 45 min at room temperature in the dark and then quenched with 5 mM DTT for 20 min at room temperature. In order to clean the proteins extracted from the lysate, a methanol–chloroform precipitation was performed, and the protein pellet was washed twice with acetone. The pellet was resuspended in 300 μl of 8 M urea, 50 mM Tris-Cl (pH 8), and the concentration of total proteins extracted from a cell line was determined using the Pierce BCA Protein Assay Kit (Pierce).

**Fig. 1:**
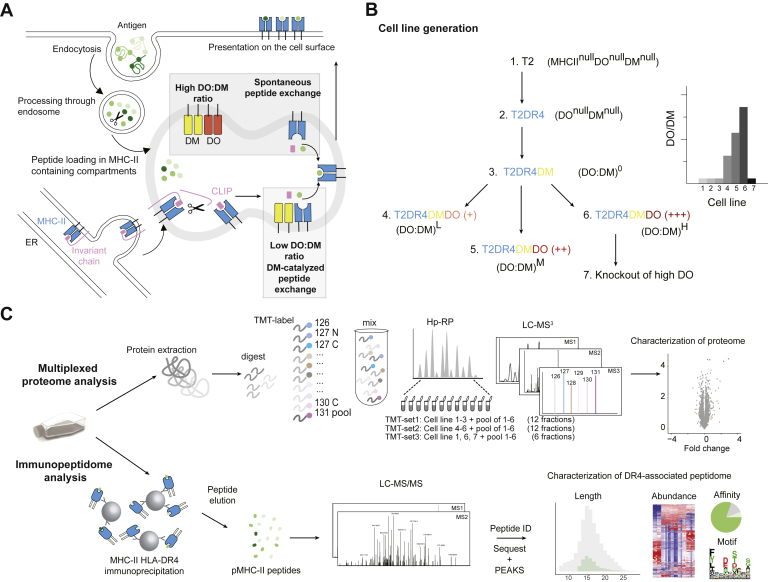
**Schematic of DO-involved antigen presentation and rationalefor experimental design.***A*, model to be tested: DO association limits the abundance of free DM that catalyzes loading of peptides derived from endocytosed antigen onto nascent MHC-II and thus influences the MHC-II peptidome. *B*, experimental design: T2-derived model cell lines expressing single MHC-II allele HLA-DR4 and different ratios of DO to DM (DO:DM) were constructed. (DO:DM)^0^, (DO:DM)^L^, (DO:DM)^M^, and (DO:DM)^H^ refer to the four DM+ cell lines with zero, low, medium, and high DO:DM. *C*, cell lines constructed in (*B*) were compared with their parental cell lines by MS-facilitated multiplexed quantitative proteomic analyses and label-free peptidomic analyses of ligands eluted from MHC-II (HLA-DR4) with respect to their identities, lengths, abundances, and predicted binding properties. CLIP, class II-associated invariant peptide.

Extracted proteins from each sample or replicate were diluted to 1 M urea, 50 mM Tris-Cl (pH 8) prior to digestion with Trypsin/Lys-C Mix (Promega) at a ratio of 1:25 (enzyme: substrate; 16 h at 37 °C). The reaction was quenched with the addition of formic acid to a final concentration of 5%. Digested peptides were desalted using a Sep-Pak C18 1 cc Vac Cartridge, 50 mg (Waters). Peptides were further labeled using tandem mass tag (TMT) reagents (Pierce) as previously described (33). In brief, each TMT reagent (0.8 mg per vial) was reconstituted in 40 μl of acetonitrile and incubated with the corresponding peptide sample for 1 h. The reaction was then quenched with a final concentration of 0.3% (v/v) hydroxylamine for 15 min at room temperature. TMT-labeled peptides were acidified with 25% formic acid to pH ∼ 2. To assess labeling efficiency, a ratio check was performed: 5 μl of TMT-labeled peptides from each cell sample or replicate were combined, desalted by StageTip (34) and then analyzed on the liquid chromatography–mass spectrometry (LC/MS) instrument (detailed methods described below). Based on the result from the ratio check, equal amounts of each individually labeled sample were then combined into a master pool to deliver a comparable average signal across all three TMT-labeling sets (see Fig. 1*C*).

TMT-labeled peptides were further desalted using a Sep-Pak C18 1 cc Vac Cartridge, 50 mg (Waters), resuspended in 10 mM ammonium formate (pH 10), and fractionated using the high-pH reverse phase (Hp-RP) fractionation approach as described (35, 36). For two of the TMT labeling sets, fractionation was performed using a 65 min + 15 min step-gradient buffer A (10 mM ammonium formate, pH 10) and buffer B (10 mM ammonium formate, 90% ACN, 10% H_2_O, pH 10) using an Agilent 1200 HPLC (Agilent Technologies). In total, 84 fractions were collected, concatenated, and combined (36) into a total of 12 fractions and dried down. All fractions were desalted using a C18-based StageTip, dried down, and stored at −80 °C until final LC-MS/MS measurement. In the case of the third TMT-labeling set (containing the DOKO line) a manual Hp-RP fractionation procedure was performed using a C18-based StageTip: a total of seven fractions were collected with sequential increases in (ammonium formate, pH = 10) concentration. The first and last fractions were combined into one fraction, whereas the remaining fractions remained distinct. This resulted in six final fractions. All fractions were desalted using a C18-based StageTip, dried down, and stored at −80 °C until final LC-MS/MS measurement.

### Mass Spectrometry–Facilitated Proteomic Analysis

Proteome-wide MS analyses were carried out as follows: TMT-labeled and Hp-RP-fractionated peptides described above were resuspended in 20 μl 0.1% formic acid, of which 10% of the material was injected onto the Dionex Ultimate 3000 autosampler and LC-system (Thermo Fisher Scientific). Peptides were separated on a 20-cm reversed phase column (100 μm inner diameter, packed in-house with ReproSil-Pur C18-AQ 3.0 m resin by Dr Maisch GmbH) over 120 min using a four-step linear gradient: 97% A (and 3% B) to 96% A for 15 min, to 75% A for 135 min, to 55% A for 15 min, and then to 5% A for 15 min (buffer A is 0.1% formic acid in water and buffer B is 0.1% formic acid in acetonitrile). Mass spectra acquisition was performed in a data-dependent mode on a Lumos Orbitrap mass spectrometer (Thermo Fischer Scientific) with precursor (MS1) scans acquired in the Orbitrap mass analyzer with a resolution of 120,000 and *m/z* scan range 400 to 1500. The automatic gain control (AGC) targets were 4 × 10^5^, and the maximum injection time was 50 ms. The most intense ions were then selected in top speed mode for sequencing using collision-induced dissociation (CID) and the fragments were analyzed in the ion trap. The normalized collision energy for CID was 35% at 0.25 activation Q. For tandem mass (MS2) scans, the AGC targets were 1 × 10^4^ and the maximum injection time was 30 ms. Monoisotopic precursor selection and charge state rejection were enabled. Singly charged ion species and ions with no unassigned charge states were excluded from MS2 analysis. Ions within ±10 ppm *m/z* window around ions selected for MS2 were excluded from further selection for fragmentation for 90 s. Following each MS2 analysis, five most intense fragment ions were selected simultaneously for higher energy collisional dissociation (HCD) MS3 analysis with isolation width of 1.2 *m/z*, normalized collision energy of 65% at resolution of 60,000, AGC target of 1 × 10^5^, and maximum injection time of 90 ms.

### Computational Interpretation of Proteomic Data

Mass spectra were initially interpreted with Proteome Discover v2.1 (Thermo Fischer Scientific) and done as described (37). In brief, the parent mass error tolerance was set to 20 ppm and the fragment mass error tolerance to 0.6 Da. Strict trypsin specificity was required allowing for up to two missed cleavages. Carbamidomethylation of cysteine (+57.021 Da), TMT-labeled N terminus and lysine (+229.163) were set as static modifications. Methionine oxidation (+15.995); phosphorylation (+79.966) on serine, tyrosine, and threonine; and N-terminal acetylation (+42.011) were set as variable modifications. The minimum required peptide length was set to seven amino acids. Spectra were queried against a “target-decoy” protein sequence database consisting of human proteins (downloaded from the Uniprot resource, June 2016, and contained *154,513* entries), 41 common contaminants, and reversed decoys of the above (38) using the SEQUEST algorithm (39). The Percolator algorithm (40) was used to estimate and remove false-positive identifications to achieve a strict false discovery rate of 1% at both peptide and protein levels. The coisolation threshold was set to 30, average reporter signal to noise was set to 10, SPS mass matches was set to 65%, and isotopic purity correction of reporter quant values was applied. Normalization was set to “total peptide amount” and scaling was done on the average of pooled controls. Known false positives (*i.e.*, decoys) and contaminants were excluded from further analysis steps. Differentially expressed proteins were identified, statistically evaluated, and visualized from log-transformed quantitative data using R, Rstudio, and Qlucore Omics Explorer v3.2 (Qlucore AB). Unless otherwise noted in the text, differentially expressed proteins were selected based on a q-value threshold <0.05 following Benjamini–Hochberg correction for multiple hypothesis testing (41).

### Purification of HLA-DR4-Associated Peptides

HLA-DR4 proteins were immunoprecipitated from T2-derived cells, and their associated peptide cargo was eluted as described (37, 42, 43), with some modifications. Briefly, each cell line was grown as two independent biological replicates, independent of cells prepared for full proteome analysis described above, and 2 × 10^8^ cells per replicate were harvested. Cells were then lysed for 20 min on ice in 20 mM Tris-HCl (pH 8), 150 mM NaCl, 1 % (w/v) CHAPS, 0.2 mM PMSF, supplemented with 1× Halt Protease and Phosphatase Inhibitor Cocktail (Thermo Fisher Scientific) and Complete Protease Inhibitor Cocktail (Roche). The lysate was centrifuged (2 × 30 min, 13,200 rpm at 4 °C), and the resulting supernatant was precleared for 30 min using rProtein A Sepharose fast-flow beads (GE Healthcare). The precleared lysate was incubated with HLA-DR-specific antibody L243 (44) (produced and purified by Genentech from hybridoma) coupled to rProtein A Sepharose fast-flow beads for 5 h at 4 °C. Following the immune-captures of DR4, the beads were washed with TBS (pH 7.4) and peptides were eluted from the purified DR4 molecules using 10% acetic acid. Eluted peptides were passed through a 10-kDa MWCO filter, followed by a concentration step using vacuum centrifugation, before being desalted on C18-based StageTips and stored at −80 °C until LC-MS/MS analysis.

### Mass Spectrometry–Facilitated Analysis of DR4 Peptidome

Peptides eluted from DR4 proteins as described above were reconstituted in 12 μl 0.1% formic acid and analyzed on an LTQ Orbitrap Elite mass spectrometer (Thermo Fischer Scientific). Samples were then injected onto the Eksigent ekspert nanoLC-425 system (SCIEX), and peptides were separated by capillary reverse phase chromatography on a 20-cm reversed phase column (100 μm inner diameter, packed in-house with ReproSil-Pur C18-AQ 3.0 m resin by Dr Maisch GmbH) over a total run time of 160 min, including a two-step linear gradient with 4% to 25% buffer B (0.2% (v/v) formic acid, 5% dimethyl sulfoxide, and 94.8% (v/v) acetonitrile) for 120 min followed by 25% to 40 % buffer B for 30 min. Each cell condition was prepared in parallel as two biological replicates, and each biological replicate was injected three times, each using a different complementary instrumental method, resulting in six raw data files per cell condition. The three instrumental methods are HCD, CID including single-charged species, and CID excluding single-charged species (42). Acquisition was executed in data-dependent mode, with full MS scans acquired in the Orbitrap mass analyzer with a resolution of 60,000 (full width at half maximum) and an *m/z* scan range 340 to 1600. The top 10 most intense ions with masses ranging from 700 to 2750 Da were then selected for fragmentation, then measured in the Orbitrap mass analyzer at a resolution of 15,000 (full width at half maximum). The ions were fragmented with a normalized collision energy of 35% and an activation time of 5 ms for CID and 30 ms for HCD. Dynamic exclusion was enabled with repeat count of 2, repeat duration of 30 s, and exclusion duration of 30 s. The minimal signal threshold was set to 500 counts.

### Identification and Quantification of DR4-Binding Peptides from Mass Spectra

All tandem mass spectra were queried against the same “target-decoy” sequence database described above for the proteome analysis. All spectra were searched using both SEQUEST (39) and PEAKS DB (Studio 8, Bioinformatics Solutions Inc) (45) search engines. The MSConvert program (version 3.0.45) was used to generate peak lists from the RAW data files, and spectra were then assigned to peptides using the SEQUEST (version 28.12) algorithm. Spectra were also interpreted by the PEAKS algorithm’s *de novo* sequencing function to improve peptide identification confidence (42). The parent mass error tolerance was set to ± 10 ppm and the fragment mass error tolerance to 0.02 Da. Enzyme specificity was set to none and oxidation (M), deamidation (N, Q), cysteinylation (C), and phosphorylation (S, T, Y) were considered as variable modifications. High-confidence peptide identifications were selected at a 1% false discovery rate with a version of the Percolator algorithm (40) which we modified for immunopeptide analysis (42). Unlike conventional proteome analysis, false discovery rates were not evaluated at the level of assembled proteins, as this would unnecessarily penalize proteins identified by just one peptide. Quantitative abundance values (MS1 peak areas) were extracted from raw data as described (37, 46). All peptide data and mass spec raw data files have been deposited in the PRIDE Archive at www.ebi.ac.uk/pride/archive (47) under accession number PXD024392.

### Characterization of DR4-Binding Cores and Prediction of Binding Affinity

Immunopeptidome datasets were evaluated with the PLAtEAU script (48) to identify DR4-binding cores. Only peptides reported in both biological replicates (with the criteria of at least observed in one of the three technical replicate injections) were considered for further PLAtEAU analysis. The minimum core length was set to 13 residues (default), and the immunopeptidome data from each T2-derived cell line were analyzed individually as well as all lines and data together. The default option, “impute with lowest measured value in each run” was enabled. The quantitative cutoff criteria for being reported as differentially presented were a *p*-value of less than 0.01 (corrected for multiple hypothesis testing using Benjamini–Hochberg (41)) and a log2 fold change either >2 or <−2. Both the entire set of peptides from the peptidome analysis and the subsequently defined core output from PLAtEAU were used as input for affinity prediction using NetMHCIIpan version 4.0 (49, 50). NetMHCIIpan-4.0 scores how well a given peptide sequence can bind an HLA-DR allele in question (*e.g.*, DRB1∗04:01). Its scoring models apply artificial neural networks trained on multiple extensive datasets that measured *in vitro* binding affinity (BA) and MS-derived eluted ligands (ELs). Optionally, NetMHCIIpan-4.0 can score binding against a model trained only on BA and not EL data. As MS data were drawn from untargeted MS studies rather than from discrete binding measurements, the two models should be expected to produce different results in some cases. We therefore designate the former prediction approach “EL” and the latter prediction approach “BA.” For both EL and BA prediction approaches, we applied rank-based thresholds of 2% and 10% to separate strong, weak, and nonbinders. See main texts and figures for details. To evaluate prevalent motifs among peptides in a more untargeted fashion we applied Gibbs cluster analyses (GibbsCluster-2.0 Server (51) with the MHC class II parameter settings) followed by visualization by Seq2Logo.

### Experimental Design and Statistical Rationale

This study was designed to include proteomic and immunopeptidomic components, which differed in the numbers of replicates and data collection modalities. Differences between these experiments were due to technical considerations, since cell input for immunopeptidome assays needed to be approximately 100-fold more than proteomic assays. Proteomic data were measured as preparative triplicates, such that each cell culture was divided into three aliquots, and each was lysed, digested, and labeled with TMT reagents in parallel as described above. Within each replicate set, all samples were processed in a single batch. LC-MS/MS analysis of HPRP-fractionated peptides proceeded according to the sequential order of the concatenated fractions, as this was not deemed to be a significant source of potentially confounding variation. LC-MS analyses were collected as single injections.

Immunopeptidomic data were collected from biological duplicates, which were processed from lysis, immunoprecipitation, and desalting at different times and in a randomized order. LC-MS analyses proceeded in a sequential order based on the nomenclature of the cell lines, one replicate set at a time. Technical triplicate LC-MS analyses were acquired per sample to increase coverage and overall signal using different experimental methods as described above. Carryover between different samples was minimized by acquiring blank analyses between each.

## Results

### Strategy for Investigating the Influence DO:DM has on pMHC-II Repertoires

Typically, antigens undergo endocytosis and intracellular processing by specialized APCs (*e.g.*, B cells) prior to pMHC-II presentation for CD4+ T cell scanning (7). Necessary steps in this process include proteolysis of the MHC-II chaperone invariant chain (Ii) to generate CLIP (Class II associated Ii peptide) that remains in the binding cleft of MHC-II and the spontaneous or DM-catalyzed CLIP removal and antigenic peptide loading (Fig. 1*A*). In B cells and several other APCs described above, this process is further modulated by DO, likely *via* the competition between DO and MHC-II for binding to DM (19). As only certain APC types express DO (8) and DO knockout models have yielded apparently contradictory immune consequences (11, 12, 13, 14, 15), the contribution of DO is puzzling. DO influences pMHC-II repertoires by controlling the level of free, active DM (17, 21, 24). High DO levels, as observed in naïve or memory B cells, limit free DM activity and permit loading of lower-affinity peptides (Fig. 1*A*). Acid-driven DO denaturation from DMDO complexes elevates free DM levels, thereby promoting the formation of stable pMHC-II (21). For instance, when the B cell receptor binds antigen, B cell activation leads to acidification of late endosomes and denatures DO (17, 52) This process is B cell receptor–antigen affinity dependent (17).

In this study, we sought to establish a tractable system to explore the peptide repertoires under varied DO:DM conditions and to elucidate the impact of DO:DM on MHC-II peptidomic landscapes. We used a series of human T x B hybrid cell lines (T2 derivatives, see Experimental procedures), each differing by only one type of protein, *i.e.*, HLA-DR4, DM and DO, or by DO:DM levels (Fig. 1*B*). To generate the cell lines with varied DO:DM, we used a polyclonal DO transfectant of T2.DR4.DM cells and two clonal lines expanded from FACS-sorted single cells, based on levels of surface CLIP/DR4 complexes (Fig. 1*B*, see also Experimental Procedures). We extracted all protein content from the full panel of cell lines for MS-facilitated proteomic analysis; in parallel, we eluted DR4-associated peptides for MS peptidomic analysis (Fig. 1*C*). This strategy allowed us to measure how DO:DM influences DR4 peptidomes, while accounting for the role protein abundance may have in peptide selection, and any subtle proteome-wide alterations that might exist between each cell line.

### pMHC-II-Specific mAbs Reflect DO:DM Influence on DR4 Peptide Presentation

**Fig. 2:**
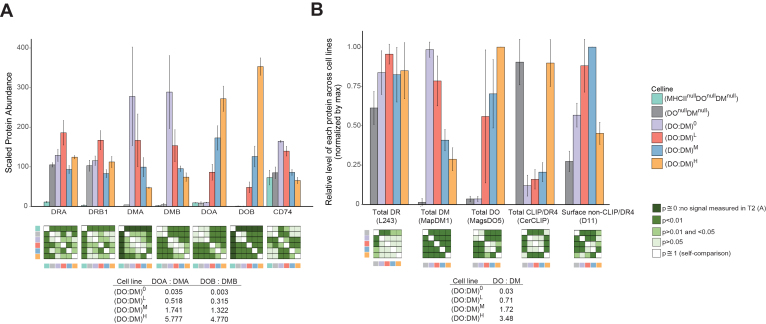
**T2-derived cell lines demonstrate anticipated abundances of HLA-DR and associated chaperones or peptides.***A*, mass spectrometric analysis of the protein abundance for the α or β chains of DR, DM, or DO, and for CD74 (the invariant chain). Data represent scaled TMT-labeled peptide signals from the corresponding protein ±SD (arbitrary units). DMA and DOB were not detected in parental T2 (DR^null^DO^null^DM^null^) cells. *B*, flow cytometric analysis showing the relative protein level for total DR, DM, DO or CLIP/DR4 complexes and surface non-CLIP/DR4 complexes. Monoclonal antibodies (mAb) used for staining are L243, MapDM1, MagsDO5, CerCLIP, and NFLD.D11 as indicated. Data represent mean fluorescence intensities of the corresponding staining in each cell line normalized to the cell line that yielded the maximum value for each given protein (set to 1) ± SD. See supplemental Fig. S1 for representative flow histograms. In both (*A*) and (*B*), a two-sample *t* test was used to compare each pair of the indicated cell lines for the indicated protein level. The calculated ratios of DO, DOA, or DOB to DM, DMA, or DMB, respectively, reflect the relative abundance differences rather than the absolute number in the four DM-expressing lines.

We confirmed the expression of DR4, DM, and DO molecules in the full panel of T2-derived stable cell lines using MS and flow cytometry. Both techniques aimed at comparing a particular protein across cell lines, rather than comparing different proteins within a given cell line. MS-facilitated proteomic analysis validated the expression of α and β chains of DR, DM, and DO (Fig. 2*A*, supplemental Datasets S1 and S2). In addition, these semiquantitative data (Fig. 2*A*) suggest that DM:DR ratios ranging from ∼1:2 ((DO:DM)^H^ cells) to ∼1:1 ((DO:DM)^L-M^ cells) to ∼2:1 ((DO:DM)^0^ cells) were captured by our cell model. Levels of the α and β components of each protein, compared across cell lines, were consistent with those of the corresponding total αβ heterodimeric protein detected by flow cytometry (Fig. 2*B*). Flow cytometry analysis further showed that DM+ lines have comparable levels of total DR and a hierarchy of total DM or DO (Fig. 2*B* and supplemental Fig. S1). The concurrent decrease of DM levels as DO levels increased was a consequence of how (DO:DM)^M^ and (DO:DM)^H^ lines were selected based on CLIP/DR4 at the cell surface. This resulted in a step-wise increase of DO:DM across these cell lines. As we observed previously (19, 21), DM expression, unopposed by DO in the (DO:DM)^0^ line, catalyzed the removal of almost all CLIP bound by DR4. In contrast, CLIP/DR4 persisted in T2DR4, which lacks DM and DO. DO expression rescued CLIP/DR4 in (DO:DM)^H^, whereas CLIP level was only slightly increased in (DO:DM)^L^ and (DO:DM)^M^ compared with (DO:DM)^0^.

Using NFLD.D11, an antibody specific for non-CLIP/DR4 (32), we further determined that (DO:DM)^H^ displayed significantly lower levels of non-CLIP/DR4 than the levels in the other DM^+^ lines. The high level of invariant chain protein (CD74, see Fig. 2*A*) found in (DO:DM)^L^ and (DO:DM)^M^ concurrent with low levels of CLIP/DR4 (Fig. 2*B*) suggests that CLIP/DR4 levels directly depend on DO:DM stoichiometry and not on CD74 protein abundance. Of interest, we found that non-CLIP/DR4 levels, as detected by NFLD.D11, were increased in (DO:DM)^L^ and (DO:DM)^M^ compared with (DO:DM)^0^ and with (DO:DM)^H^. This observation of discordant CLIP/DR4 and non-CLIP/DR4 abundances (Fig. 2*B*) is consistent with the high likelihood that NFLD.D11 stains a limited subset of non-CLIP/DR4 complexes. One possible explanation for these data is that DR4 peptide ligands in NFLD.D11-stained complexes require enough DM activity for CLIP exchange but not so much as to facilitate replacement by other, non-NFLD.D11-reactive peptides that are more DM-resistant (*i.e.*, found in (DO:DM)^L^ and (DO:DM)^M^ *versus* (DO:DM)^0^ cells). Thus, these data suggest that some DR4-presented peptides require intermediate levels of DO:DM and therefore are repressed when DO:DM is excessively low or excessively high.

Overall, these results indicate that graded DO:DM can have either substantial or nuanced influences on pMHC-II presentation. Thus, the T2-derived lines described here represent an enhanced system for directly measuring DO:DM impact on HLA class II peptidomes. Of note, DO:DM can be directly measured, whereas its functional consequence, free, active DM, cannot. Thus, we have designated cell lines by this ratio. Across the full panel of cell lines, free DM is highest in the (DO:DM)^0^ cells and decreases as the DO:DM ratio increases. It is fully absent in the DO^null^DM^null^ cells that simulate a DM-negative condition to evaluate DM-independent peptidomes, like the ones eluted from DM-resistant MHC-II alleles (53, 54, 55).

### DR4 Peptidomic Differences Between Cell Lines Corroborate Synergistic DO:DM Tuning

**Fig. 3:**
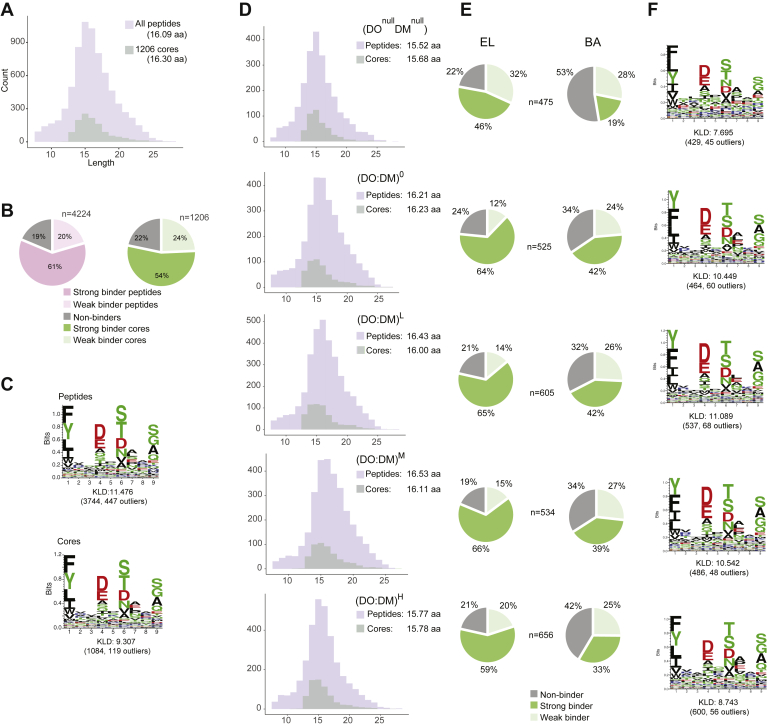
**Characterization of the combined DR4 peptidome from five cell lines or peptidomes from each individual line.***A*, length distribution for MS-identified unique peptides from all five cell lines and their cores determined using the PLAtEAU algorithm (supplemental Fig. S2*A*). The mean lengths of each are indicated in *parentheses*. *B*, percent of peptides or cores predicted to be strong (top 2% rank), weak (2%–10% rank), or non- (bottom 90% rank) DR4 binders as predicted by NetMHCIIpan-4.0 (EL approach, see Experimental Procedures). *C*, DR4 motifs generated with Seq2Logo. All peptides (*top*) or all cores (*bottom*) described in (*B*) were first clustered with GibbsCluster. Motifs indicate frequencies of particular residues at each of the nine positions of the DR4 binding register, with increased amino acid specificity at each position (anchor residue positions: P1, P4, P6, P7, P9). The top-ranked cluster for each condition is presented. The Kullback–Leibler Distance (KLD) score is listed and the size of the cluster and number of outliers is listed in *brackets*. *D*–*F*, analyses of length distributions (*D*), percent of cores predicted to be strong or weak binders using the eluted ligand (EL), and *in vitro* binding affinity (BA) models implemented by NetMHCIIpan-4.0 (see Experimental Procedures) (*E*), and motifs deduced from DR4-binding registers (supplemental Fig. S3*A*) (*F*) identified from each individual cell line.

To evaluate how DO:DM affects DR4-presented peptide repertoires, we eluted peptide ligands from DR4 in each cell line and analyzed them by MS (supplemental Dataset S3). We identified an average of 3072 unique peptides per biological replicate analysis per cell line and 10,587 unique peptides from the entire dataset (supplemental Table S1 and supplemental Dataset S3). These peptides had an average length of 16.6 amino acids (aa) (Fig. 3*A*), an appropriate average length for MHC-II-associated peptides. Peptides with no quantified signal were excluded from further consideration, resulting in 7390 quantified unique sequences. Of these, 4224 (4528 if considering different residue modifications) were quantified in both bioreplicates.

Most MHC-II-eluted peptides we identified could be grouped into nested sets of overlapping peptides. This is a common observation, resulting from the MHC-II open-ended binding groove that permits peptides of multiple lengths to bind (56). This peptide sequence heterogeneity can complicate subsequent computational procedures, such as sequence motif determination and ligand quantification. We implemented the PLAtEAU algorithm (48), which addresses this challenge by condensing overlapping peptides to single DR4-binding cores. These cores represent consensus sequences shared by nested sets of peptides, each of which should contain the same binding register with the expected P1, P4, P6, and P9 anchor positions that directly interface with the MHC-II-binding groove. Applying this approach effectively simplified our subsequent analyses, while aggregating analyte signals that were sometimes distributed across multiple peptides (supplemental Fig. S2). PLAtEAU determined 1206 unique cores with an average length of 16.30 aa (Fig. 3*A* and supplemental Table S2). Each of these cores, which mapped to 782 self-proteins (supplemental Fig. S2), contained at least one 9-aa register expected to occupy the DR4 peptide-binding groove.

To systematically assess thousands of peptides in terms of predicted DR4 binding capacities (regardless of DO:DM), the NetMHCIIpan-4.0 algorithm proved to be a useful tool with appreciable accuracy and throughput advantages (49, 50). NetMHCIIpan-4.0 predicted that 81% of 4224 peptides and 78% of 1206 cores ranked among the top 10% of sequences able to bind DR4 (Fig. 3*B*). The DR4 binding motifs generated from the entire peptidomic dataset and from the condensed cores (Fig. 3*C*) corresponded well with the reference motifs generated using the NetMHCIIpan-4.0 motif viewer (supplemental Fig. S3). Collectively, the core dataset and the MS-derived peptide dataset from which it is derived both cover informative DR4-presented peptides with expected lengths, motifs, and predicted binding features, and both are therefore appropriate for further analysis.

We next compared the condensed core pMHC-II repertoires derived from each individual cell line. Compared with DO^null^DM^null^ or (DO:DM)^H^, DM^+^ lines with zero to medium DO:DM showed a tendency toward longer peptides or cores (Fig. 3*D* and supplemental Fig. S4*B*). These lines, which have modest DO:DM, also yielded a greater proportion of predicted strong DR4-binding cores (Fig. 3*E*) and similar motifs to one another (Fig. 3*F*). The amino acid residue preferences at the four dominant anchor positions (P1, P4, P6, P9) were most similar among the (DO:DM)^0-M^ lines, suggesting a DM-mediated preference for Y over F at P1. We note, however, that the top two most frequent residues at each of the anchor positions were conserved among all five lines (Fig. 3*F*). The (DO:DM)^H^ line generated the greatest number of cores (656), compared with (DO:DM)^0-M^ (Fig. 3, *E* and *F*); this indicates that the presence of DO enables a larger number and an increased diversity of peptides to be presented by DR4, as also observed by Nanaware *et al.*, (30) for DR1. These differences are consistent with greater free DM levels in (DO:DM)^0-M^ than (DO:DM)^H^, with consequent selection for high affinity, stable pMHC-II complexes with decreased sequence diversity. Equivalent results between peptide and core sequences (supplemental Figs. S4 and S5) support the nonredundant core analysis as an appropriate data simplification tool.

### Restricted DO:DM Ratios Tune Distinct Subsets of Peptides for DR4 Presentation

**Fig. 4:**
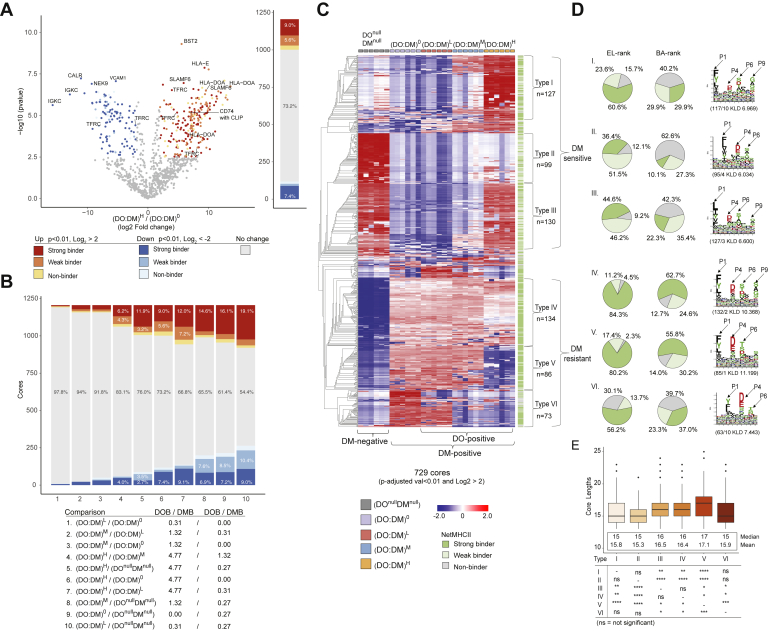
**Classification of distinct core types.***A*, a representative volcano plot illustrating abundance differences of cores between two cell lines, (DO:DM)^H^ and (DO:DM)^0^. Significance cutoff: *p* < 0.01 of *t* test after being adjusted for multiple hypothesis testing (BH) and a log2 fold change >2 (in *red color scale*) or <−2 (in *blue color scale*). Strong, weak, or nonbinders predicted using the NetMHCIIpan-4.0 EL model are indicated by different color intensities. The source proteins of a selected subset of cores are indicated. The stacked bar to the *right* aggregates the seven types of data points displayed in the volcano plot. *B*, summary of all pair-wise comparisons between five cell lines. The stacked bar chart is based on differential presentation, similar to that described in (*A*). Cell line details with the MS-determined DO:DM are indicated. *C*, heatmap (z-score normalized and hierarchically clustered) showing 729 cores with significantly different abundances between at least two cell lines using criteria described in (*A*) and classification of six core types. *Green* scales to the right indicate the corresponding core’s binding prediction using NetMHCIIpan-4.0’s EL model. The six columns per cell line represent two biological replicates, each with three technical replicate LC-MS analyses. *D*, percentage of type I–VI cores predicted to be strong-, weak-, or nonbinders using EL or BA approaches (see Experimental Procedures), and the corresponding sequence motif extracted from each, as described in Figure 3. *E*, length distribution for the six types of cores. A two-sample *t* test was used to compare the lengths of each pair of the six core types.

From a global perspective, differences between each cell line were most apparent relative to DO^null^DM^null^ or comparing (DO:DM)^H^ with (DO:DM)^0-M^ (Fig. 3). Within any of these comparisons, however, specific PLAtEAU-deduced cores demonstrated a range of abundance profiles. To develop a more granular understanding of DO:DM influence on antigen presentation, we directly compared the abundances of each core between all cell line pairs (Fig. 4, *A* and *B*). We found smaller differences between each of the three (DO:DM)^0-M^ cell line pairings and larger differences between (DO:DM)^0-M^ and DO^null^DM^null^, as compared with the differences observed between the other pairs of cell lines (Fig. 4*B*, comparisons 1–3 or 8–10 *versus* comparisons 4–7). This analysis confirmed the greater similarity between DR4 peptidomes derived from the three (DO:DM)^0-M^ lines, as mentioned above (Fig. 3, *E* and *F* and supplemental Fig. S2*A*). This grouping is surprising considering that (DO:DM)^L^ and (DO:DM)^M^ expressed DO, whereas (DO:DM)^0^ did not. This suggests that many cores did not simply increase or decrease monotonically with tuning the DO level itself; rather they might depend on a synergistic tuning of DO:DM instead. Particular DO:DM thresholds might therefore promote some peptides’ efficient loading onto DR4 while inhibiting others.

To explore DO:DM tunable profiles further, we clustered cores based on their relative abundance across cell lines, as quantified by MS (Fig. 4*C*). We found that most (729 of 1206, supplemental Table S3) cores were presented with substantially distinct abundances between any two of the five cell lines we examined. Accordingly, this clustering analysis indicated six major core types: type I: (DO^null^DM^null^, (DO:DM)^0-L^) < (DO:DM)^H^; type II: (DO:DM)^0-H^ < DO^null^DM^null^; type III: (DO:DM)^0-M^ < ((DO:DM)^H^, DO^null^DM^null^); type IV: DO^null^DM^null^ < (DO:DM)^0-H^; type V: (DO^null^DM^null^, (DO:DM)^H^) < (DO:DM)^0-M^; type VI: (DO^null^DM^null^, (DO:DM)^M-H^) < (DO:DM)^0-L^. The majorities of types II and III (up to 89.9%) were predicted by NetMHCIIpan-4.0 as weak or nonbinders, whereas the majorities of types IV and V (up to 84.3%) were predicted as strong binders, regardless of prediction approaches (El *versus* BA) used (Fig. 4*D*). The deduced motif types associated with these core types were related to the canonical DR4 binding motifs (supplemental Fig. S3).

These hierarchical clusters (Fig. 4*C* and supplemental Fig. S2*A*) further suggest cores fall into two primary categories: DM-sensitive (types I–III), which showed low abundance in the (DO:DM)^0-M^ lines, and DM-resistant (types IV–VI), which showed high abundance in (DO:DM)^0-M^ lines, as compared with DO^null^DM^null^ cells. DM-sensitive cores may spontaneously bind DR in the absence of DM, notably in DO^null^DM^null^ cells. These cores may be readily displaced or competed off by DM-resistant cores if DM is present. This appears to be the case particularly for types II and III, half of which were predicted by NetMHCIIpan-4.0 to be weak binders (EL-rank, Fig. 4, *C* and *D*). For others such as types I and III, binding to DR4 in the presence of DM can be protected with high DO:DM, which effectively opposes DM activity. In contrast, DM-resistant cores were presented almost exclusively in the presence of DM, and many were predicted to be strong DR4 binders (Fig. 4, *C* and *D*). These data suggest that some cores, such as those of type IV can be selected for DR4 presentation at higher DO:DM condition when there is less, but apparently adequate, free DM activity. In contrast, other cores like those of type VI may not be presented until DO:DM reaches very low levels.

We note that type II cores (DM sensitive, primarily found in DO^null^DM^null^ cells) were shorter, on average, than the other types (mean length = 15.3; Fig. 4*E*) and further diminished in length compared with all cores observed from DO^null^DM^null^ cells (mean length = 15.68, Fig. 3*D*). Conversely, type V cores (DM resistant, primarily excluded from DO^null^DM^null^ and (DO:DM)^H^ cells) were longer on average than the other types (mean length = 17.1, Fig. 4*D*), which is substantially longer than the average core length recorded from any single cell line (maximum mean length = 16.23 for (DO:DM)^0^; Fig. 3*D*). We further note that type II cores and type VI cores (DM resistant, primarily identified in (DO:DM)^0-L^ cells) showed an unusual register shift of the anticipated dominant anchor residues (Fig. 4*D*; P1 is located at the third position in these cores, whereas it is in the first position in the other types). This shift implies longer N-terminal extensions among the underlying peptides. We cannot rule out the possibility that this apparent register shift was due to algorithmic limitations and suboptimal input amount (<100 core sequences) (see also Discussion). However, we found a modest bias toward longer flanking N termini among DO^null^DM^null^ cells, but not in (DO:DM)^0^ cells, which yielded the most abundant type VI cores (supplemental Fig. S6). We conclude from these data that DO:DM does not substantially influence the register positioning within peptides presented by DR4 in these cells, from a peptidomic perspective. In addition, motif analyses showed no amino acid preference at positions other than the anchor residue positions within the 9-aa register themselves across all cell lines (Fig. 4*D* and supplemental Fig. S7).

### pMHC-II Characteristics Contribute to DO:DM-Tuned Peptide Presentation

**Fig. 5:**
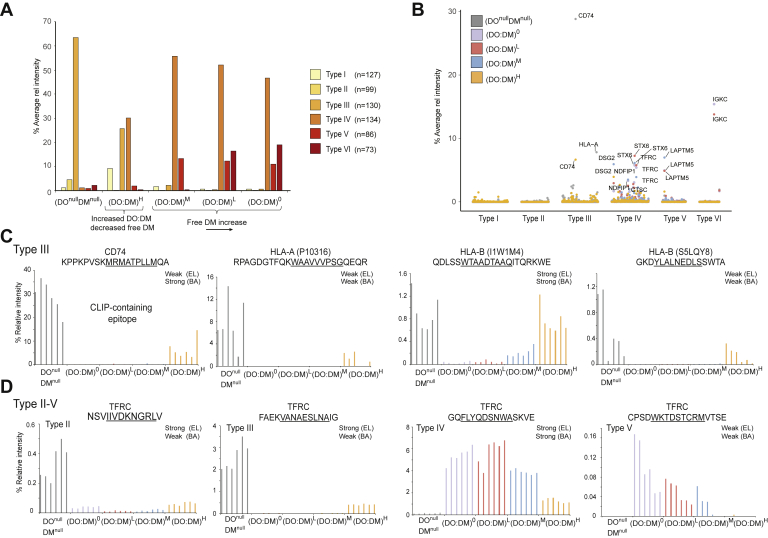
**Abundance comparisons between each core type across the DO:DM gradient.***A*, percentage abundance subtotal of each type of core (clustered in Fig. 4*C*). Percentage relative average intensity spanning the two biological replicates displayed for the six types. In addition, the residual % relative intensity covering all the six subtypes (*I*–*V*) is also displayed. *B*, percentage abundance subtotal of each core (clustered in Fig. 4*C*). *C*, four examples of type III cores from CD74, HLA-A, and HLA-B. *D*, example of cores for type II–V for the transferrin receptor protein 1 (TFRC).

The intracore Z-score normalization we used to generate Figure 4*C* effectively showed relative abundance changes between each cell line for a given core but could mask gross abundance differences between the various cores we measured. To further examine how tuning DO:DM can affect different subsets of peptides presented by DR4, we compared the relative abundances of each core type across all cell lines (Fig. 5*A*). Despite the similar numbers of cores clustered into each of the three DM-sensitive core types (Fig. 4*C*), types I and II showed substantially lower abundances than type III (Fig. 5*A*). Thus, types I and II account for a small contribution to the overall peptidome in any of the cell lines. In contrast, the abundances associated with the DM-resistant cores of types IV–VI appeared to scale approximately with the number of cores represented in each group (Figs. 4*C* and 5*A*). Of these, type IV was the most abundant core type in the three cell lines with substantial DM activity ((DO:DM)^0-M^) (Figs. 4*C* and 5*A*), but notably, a strong binding type IV subset was enriched when DM activity was most limited by DO (Fig. 4*C*).

To better understand how DO:DM effects might differentially apply to specific DM-sensitive or -resistant peptides, we evaluated how cores’ abundances (supplemental Table S2) contributed to these type-specific observations (supplemental Fig. S8). Among the DM-sensitive type III core sequences, the CLIP (CD74) sequence, KPPKPVSKMRMATPLLMQA (binding register underlined), was dominant, with 28.8% normalized intensity in DO^null^DM^null^ and 6.66% in (DO:DM)^H^ (Fig. 5*B*). This core represents the most abundant overlapping peptide sequences in our entire dataset (supplemental Dataset S3). The CLIP core demonstrated greatly reduced abundance in (DO:DM)^0-M^ cells (0.05%, 0.14%, and 0.20%, respectively; Fig. 5*C*). The trend was consistent with the flow cytometric measurements of CLIP/DR4 (Fig. 2*B*) and confirms the known role DO and DM have on catalyzing peptide exchange for CLIP. The second most abundant DM-sensitive type III core (RPAGDGTFQKWAAVVVPSGQEQR), with 7.8% normalized intensity in DO^null^DM^null^ (Fig. 5, *B* and *C*), was derived from the HLA-A class I protein. It also followed a similar decrease in abundance (normalized intensities of 0.02%, 0.01%, 0.03%) across the (DO:DM^0-M^ gradient) with a substantial increase in (DO:DM)^H^ (1.2%, Fig. 5*C*). Two HLA-B-derived cores demonstrated similar DM-sensitive profiles across the five cell lines, albeit with 10-fold lower intensities (Fig. 5*C*). A majority of DO:DM-dependent reductions in peptide abundance did not reflect any underlying protein abundance differences, and protein abundances largely were consistent across the five cell lines (supplemental Fig. S9). Exceptions were expected, such as a core derived from HLA-DOA, which was undetectable in the DO^null^DM^null^ cell line. Overall, these results support the notion that DM can select against subsets of weak-binding peptides in a DO-dependent fashion.

In type III DM-sensitive cores, large abundance swings between cell lines (Fig. 5*A*) tended to be dominated by a small number of cores (*e.g.*, CLIP/CD74) in DO^null^DM^null^ and (DO:DM)^H^ cells (Fig. 5*B*). In contrast, the greater abundance of DM-resistant cores of types IV-VI among (DO:DM)^0-M^ cells (Fig. 5*A*) can be attributed to a more diverse set of cores, each with moderately high abundance, for example, cores derived from DSG2, IGKC, LAPTM5, NDFIP1, STX6, and TFRC (Fig. 5*B*). These were mostly concentrated among type IV cores, whereas types V and VI core abundances were more restricted to single protein sources (LAPTM5 and IGKC, respectively). As with the DM-resistant cores, these patterns relative to DO^null^DM^null^ cells cannot simply be attributed to abundance differences at the protein level (supplemental Fig. S9). Instead, these three DM-resistant core types appear to have distinct DO:DM thresholds that govern their presentation by DR. We attributed this differential peptide presentation to the graded DO:DM effects on DM selection for high-affinity, stable peptide/DR4 complexes, as most related cores were predicted by NetMHCIIpan-4.0 to be strong binders.

These data further demonstrate that single proteins can harbor cores of multiple types. For example, we identified multiple cores across the length of TFRC with moderate to high abundance belonging to four core types (II–V) (Fig. 5*D*): in agreement with the aggregated quantifications of DM-sensitive peptides (Fig. 5*A*), the type III core, FAEKVANAESNAIG, was approximately 10-fold more abundant than the type II core, NSVIIVDKNGRLV, in DO^null^DM^null^ and (DO:DM)^H^ cells, but neither were robustly presented in (DO:DM)^0-M^ (Fig. 5*D*). In contrast, the type IV DM-resistant core, GQFLYQDSNWASKVE, decreased with increasing (DO:DM)^0-H^ (Fig. 5*D*). This most abundant core from TFRC (up to 6% relative intensity in (DO:DM)^0-L^) was predicted to be a strong binder by both EL and BA models (Fig. 5*D*). It is noteworthy that the type V DM-resistant TFRC core, CPSDWKTDSTCRMVTSE, also decreased with increasing (DO:DM)^0-H^ (Fig. 5*D*). Of the 40 peptides containing this core that were mapped in the (DO:DM)^0^ line, 35 were cysteinylated on the N-terminal cysteine residue, whereas only two peptides containing this core were mapped in the (DO:DM)^H^ line for this core (supplemental Dataset S3). These data suggest that, even when considered together, intrinsic binding affinities and protein abundance only partially influence the extent of peptide presentation by DR4. Instead, these examples demonstrate that, even when protein levels are fairly constant, tuning DO:DM ratios not only can change the abundances of individual peptides, it can shift which cores from a given protein are most robustly presented. For some, an intermediate DO:DM ratio may be optimal for presentation by DR4 as our prior staining with the NFLD.D11 antibody suggested (Fig. 2*B*).

### DO Knockdown Supports DO:DM Tuning of DR4-Presented Peptides

**Fig. 6:**
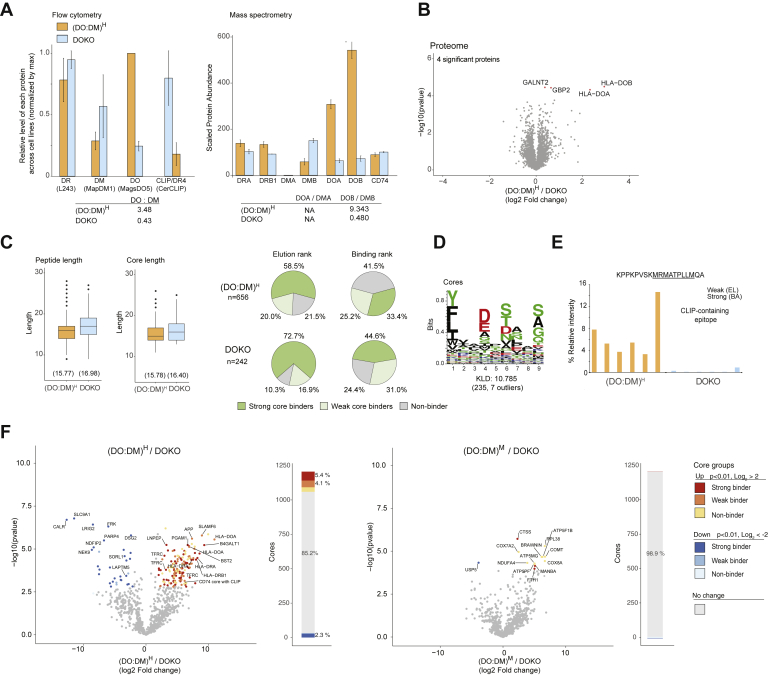
**CRISPR-mediated transformation of high DO:DM to low replicates (DO:DM)**^**L**^**cell phenotype.***A*, flow cytometric analysis and mass spectrometry quantification of DR, DM, DO, and CLIP. Relative protein expression levels between the cell lines for DR, DM, DO, and CD74 based on total cell lysate mass spectrometry–based TMT-proteomics data. No peptides were sequenced from the *DMα* (*DMA*) gene in this TMT-labeling set, resulting in undefined ratios involving this protein. *B*, minimal differential proteome expression differences were observed between the (DO:DM)^H^ line and its knockout. Differentially upregulated expressed proteins with a q-value of <0.05 in the (DO:DM)^H^ line are indicated in *red*. *C*, peptide length, core epitope length, and binding prediction. *D*, DOKO motif generated as in Figure 3*C*. The Kullback–Leibler Distance (KLD) score is listed and the size of the cluster and number of outliers is listed in *brackets*. *E*, DOKO demonstrated markedly reduced levels of the dominant CLIP-containing core. *F*, differential presentation between DOKO cells and its parental (DO:DM)^H^ line or the lower DO:DM cell line (DO:DM)^M^. As in Figure 4*A*, data are presented both as volcano plots and stacked bar charts based on differential presentation (*t* test, with a *p* < 0.01 after being adjusted for multiple hypothesis testing (BH) and a Log_2_ fold change larger than 2 or smaller than −2). Cores with different binding affinities (predicted by NetMHCIIpan-4.0) using elution rank are indicated by different color intensities. Proteins that gave rise to a selected subset of relevant cores are indicated.

DO is downregulated in naïve and memory B cells that enter GC for antigen presentation and affinity maturation of their antigen receptors. To mimic this regulation and to verify our model, we used CRISPR (31) to knock down DO levels in (DO:DM)^H^ cells (DOKO) and measured the corresponding DR4 peptidomes and proteomes using the aforementioned strategy (Fig. 1*C* and supplemental Fig. S10). Flow cytometric assays indicated lower levels of the DO heterodimer in the DOKO cell line compared with (DO:DM)^H^ cells (Fig. 6*A* and supplemental Fig. S10). This was confirmed by MS-based proteomic measurements of the DO α and β chains (Fig. 6*A*). Of note, using CRISPR to edit the *DO* gene preserved a similar degree of cell heterogeneity for DO expression (supplemental Figs. S10 and S11). This should also reflect a similar scope of DO downregulation as might be observed in physiologic contexts. We further confirmed that 99.9% (4060 of 4064) quantified proteins (TMT-label set 3) demonstrated consistent expression between the DOKO and (DO:DM)^H^ cell lines (Fig. 6*B*). Notable exceptions included the DO α and β chains (both *p* < 0.01 and log2 fold change >2) that we specifically targeted by CRISPR.

Consistent with the aforementioned difference between cores presented in (DO:DM)^H^ and (DO:DM)^0-M^, increased free DM in the DOKO resulted in longer peptides and longer cores, a larger proportion of which were predicted strong binders (Fig. 6*C*). The sequence motif generated from the DOKO peptidomic data (Fig. 6*D*) was more homologous to that generated using the (DO:DM)^0-M^ peptidomic data (Fig. 3*F*) than either DO^null^DM^null^ or (DO:DM)^H^ cells, consistent with free DM activity. As expected, a drastic reduction in the dominant CLIP containing core was observed in the knockout (Fig. 6*E*). In keeping with their lower DO:DM ratio, pair-wise volcano plot comparisons indicated that DR4-presented cores in DOKO cells were qualitatively more similar to those presented in (DO:DM)^M^ cells than in (DO:DM)^H^ cells (Fig. 6*F*), consistent with the overall expected peptidomic outcome after DO:DM was reduced (supplemental Fig. S11*A*). Considering all cores mentioned earlier (Fig. 5, *C* and *D*), abundances in DOKO changed from the level in (DO:DM)^H^ to a level that was similar to (DO:DM)^0-M^ (supplemental Figs. S11, *B* and *C* and S12). These changes were dependent on decreased DO expression and the corresponding tuning of DO:DM, not the differential expression of the proteins from which these cores were derived (supplemental Fig. S9). Collectively, this knockdown study demonstrates that a major function of DO expression in specialized APCs is to maintain an appropriate DO:DM ratio, the tuning of which controls DM editing and the resulting DR4-presented peptide repertoires.

## Discussion

In previous studies, we measured correlations between DO:DM and DM-catalyzed peptide exchange using soluble proteins (21), B cell lines, and *ex vivo* human tonsillar B cells (17) as three model systems. Our findings suggested that the tunable DO:DM is a crucial factor governing free DM levels and the consequent spectrum of presented peptides. This kind of regulation could help explain how different APCs expressing the same complement of MHC-II alleles and exposed to the same antigens might present different peptides in different contexts. As naïve and memory B cells enter the GC, and as certain dendritic cells mature, they downregulate DO from otherwise high expression levels. Accordingly, these APCs can experience widely ranging DO:DM states and, consequently, pMHC-II repertoires bearing markedly different DM editing signatures. If one were to simply consider DO expression as either on or off, many antigen-specific outcomes, particularly when measured as T-dependent immune responses, would not be explained (11, 14, 24, 25, 26, 27, 28, 29, 30).

We sought an experimental compromise between the limited information that can be gleaned by comparing just two DO expression states (DO+ *versus* DO-) *versus* the technical challenge of precisely recapitulating widely ranging DO and DM expression levels spanning many different types of antigen-presenting cells. We therefore developed a model system in which DM and DO levels were varied with respect to one another, resulting in a graded range of DO:DM stoichiometries. We report here five new cell lines that span three DO+ states and two DO- states. We used these cell lines to approximate “infinitely” opposed DM (*i.e.*, No DM function in DO^null^DM^null^ cells), and an increasing gradient of DM activity, based on high, medium, low, and zero DO:DM ratios. MS-facilitated peptidomic and proteomic measurements demonstrated the critical tuning function DO:DM can have in modulating HLA-DR4-presented peptidomes.

Two major categories of peptides, as represented by their PLAtEAU-deduced cores, emerged from these data: those that depended on there being minimal, if any, DM activity (“DM-sensitive” types I–III, Fig. 4*C*) and those that depended on or can tolerate at least some DM activity for their presentation and detection (“DM-resistant types IV–VI, Fig. 4*C*). In particular, we found that decreasing DO:DM yielded increasingly varied peptide subsets ([DO:DM]^H^ presented type IV peptides; [DO:DM]^M^ presented types IV-V; [DO:DM]^0-L^ presented types IV–VI; Fig. 4*C*). Concurrently, a large proportion of DM-sensitive peptides were measurable when DM activity was effectively opposed by DO in (DO:DM)^H^ cells (types I and III peptides, Fig. 4*C*) or when DM was “infinitely” opposed in (DO:DM)^null/null^ cells (type III peptides, Fig. 4*C*). Knocking down DO relative to (DO:DM)^H^ cells (highest DO level) resulted in a DO:DM state similar to that of (DO:DM)^M^ cells (Fig. 6*F*). Together, these data predict that, upon antigen exposure, DO downregulation in DO-expressing APCs would favor increased MHC-II loading of DM-resistant peptides and diminished loading of DM-sensitive ones.

We note that the increased repertoire of DM-resistant peptides we found with decreasing DO:DM roughly correspond with predicted binding affinities (Fig. 4*D*). It should be stressed, however, that binding predictions alone were poor predictors of DM sensitivity and resistance (Fig. 4*D*). Many self or foreign peptides hold the potential to bind DR with high affinity and should satisfy the models employed by computational prediction tools such as NetMHCIIpan-4.0. However, the peptides that survive DO:DM-tuned DM editing can be selected based on multiple factors that are difficult to model. These include the abundance of source proteins and their sequence susceptibility to proteolysis; inherent allelic characteristics and intracellular modifications of MHC-II that influence binding specificities; intracellular modifications of source proteins or their peptide derivatives; and endolysosomal locations where proteins are degraded or protected in a pH-dependent fashion. Our MS-based peptidomic and proteomic analyses across seven cell lines with varying DO:DM ratio states empirically revealed several pMHC-II presentation characteristics related to these factors, in addition to DO:DM tuning, as discussed below.

First, a spectrum of DO:DM can correspond with different free DM activity levels, and consequently different types of peptides that survive DM editing. Although DO can block DM-catalyzed CLIP removal from DR4 (57), our model demonstrated that CLIP can be effectively replaced by other peptides when DO:DM is reduced to moderate levels (*i.e.*, (DO:DM)^M^ or even (DO:DM)^H^, Figs. 4 and 5). For example, type IV cores with high predicted binding affinity (both BA and EL models) like GQFLYQDSNWASKVE from TFRC (Fig. 5*D* and supplemental Fig. S12) were presented by all cells with any DM activity, including (DO:DM)^H^. In contrast, other cores with low predicted binding affinities (BA model) were either included (FAEKVANAESLNAIG from TFRC) or excluded (CPSDWKTDSTCRMVTSE from TFRC) among cores presented by (DO:DM)^H^ cells. Although these data point to nuanced HLA-DR4 presentation patterns (Fig. 4*C*), we note that the specific patterns observed here likely are not universal. Other MHC-II alleles can have wide-ranging thresholds at which DO:DM or free DM levels promote CLIP removal and peptide exchange. To an extreme extent, some alleles, *e.g.*, HLA-DQ2 and HLA-DQ8, have evolved to require very high DM expression to overcome their intrinsically low DM susceptibility (53, 54, 55). The DRB1∗04:01 allele studied in our model has lower affinity for CLIP than many other MHC-II alleles (58). As a result, spontaneous CLIP displacement by other peptides was observed when free DM levels were zero or low, in DO^null^DM^null^ and (DO:DM)^H^, respectively, and likely occurred across all DO:DM states in these DR4-positive cells.

Second, in addition to the underlying DO:DM context, a peptide’s abundance, binding affinity, and DM sensitivity influence the likelihood it will measurably contribute to pMHC-II repertoires. Our assessment of binding strength was based on scoring with predictive algorithms (NetMHCIIpan-4.0’s BA and EL models) rather than direct measurement. Nonetheless, it is likely true that eluted peptides from each DO:DM cell state and across all six types of cores include both strong and weak binders. However, a large proportion of DM-sensitive cores were predicted to be weak or nonbinders (ranging from 39.3% for type I EL-rank to 89.9% for type II BA-rank, Fig. 4*D*). Prominent examples include the CLIP peptides derived from CD74 and RPAGDGTFQKWAAVVVPSGQEQR from HLA-A (Fig. 5*B*). Conversely, a large proportion of cores among DM-resistant types IV–VI from (DO:DM)^0-M^ were predicted to be strong binders (*e.g.*, cores derived from STX6, LAPTM5, and TFRC) (Figs. 4*D* and 5*B*). Therefore, we propose that APCs can be regulated by DO:DM tuning, allowing more peptides with wider affinity ranges to be presented when DO:DM is high and the degree of DM editing is low. One caveat to this notion is that our analysis was based on isolating total DR4 rather than surface-expressed molecules; thus in (DO:DM)^0-M^ cells, pMHC-II with weaker binding peptides might represent intermediates along the presentation pathway.

An implication of these findings is that, when building MHC-II binding prediction models that incorporate MS eluted peptide (EL) data, it could be useful to account for DO:DM and its correlation with abundant peptides that might otherwise be deemed weak binders. Such a model could, for example, be appropriately applied to naïve or memory B cells’ repertoires as opposed to activated B cells, which have high and low DO:DM, respectively. Relatedly, motif prediction, which is integral to binding prediction, also should consider the possible contribution of high DO:DM *versus* low DO:DM. For example, we observed a swap of the P1 dominant residue (F->Y) between cells with highly or “infinitely” opposed DM ((DO:DM)^H^ and DO^null^:DM^null^) *versus* cells with appreciable DM activity ((DO:DM)^0-M^ (Fig. 3*F*)). A similar P1 residue swap was also found when comparing the EL- *versus* BA-based motif models (supplemental Fig. S3). These data suggest that DM editing could select alternate binding registers between different DO:DM states.

Third, DO:DM tuning in APCs likely affects the length of presented peptides: longer peptides were presented in (DO:DM)^0-M^ as compared with DO^null^DM^null^ and (DO:DM)^H^ cells (Fig. 4*E*). A DO:DM influence on peptide length can affect T cell recognition and peptide immunogenicity (59, 60, 61). A peptide’s length can also influence its ability to bind DR in several ways: longer peptides imply more residues flanking a peptide’s canonical 9-mer binding register, and these flanking residues can enhance MHC-II binding and increase DM resistance (62, 63). Alternatively, if a long peptide sits in the MHC-II-binding groove using a register biased toward the peptide’s C terminus (supplemental Fig. S6), additional N-terminal amino acids extending beyond the binding groove could create steric interference for DM access, as DM associates with MHC-II from the N-terminal end of the loaded peptide (23, 64). Longer peptides could also provide alternative binding registers. Furthermore, specific amino acids within these flanking sequences have been shown to modulate individual peptides’ binding affinities for DR molecules (62, 65). Our analysis of flanking sequences across all core types (supplemental Fig. S7) did not suggest these kinds of amino acid biases extend to wider peptide populations. This observation would be expected if the position of critical flanking amino acids can vary from peptide to peptide, even while the binding register sequence is consistent. It is also possible that the HLA-DR4 allele studied here is not as dependent on these flanking sequences as the alleles studied in prior reports. However, based on these data, it seems that specific amino acid–position biases in flanking sequences do not broadly account for the differential presentation we observed across the DO:DM ratios examined here. Another explanation for this increasing peptide length trend observation could relate to endosomal processing: highly opposed DM in (DO:DM)^H^ cells might not show any DM activity except in acidic late endosomes (17), where peptides tend to be extensively proteolyzed and thus are short in length. In contrast, increased free DM activity in (DO:DM)^0-L^ cells could promote peptide exchange in earlier, less acidic endosomal compartments, which likely contain longer and less processed peptides for DR binding.

Last, DO:DM tuning in APCs may affect immunodominant T cell epitope selection from across the sequence of a given protein. One such source protein, TFRC, generated multiple DR4-binding cores belonging to four different major types (II–V). We identified multiple cores derived from the TFRC protein with substantially different abundances in DO^null^DM^null^ and (DO:DM)^H^ cells as compared with in (DO:DM)^0-M^ cells, corresponding with these cells’ limited and appreciable amounts of free DM activity, respectively. These differences were independent of TFRC protein’s abundance across these cell states and mostly consistent with the intrinsic DM sensitivity, DR4 binding affinity, and abundance characteristics of each pMHC-II mentioned above. This observation leads us to speculate that, upon antigen stimulation, DO-expressing APCs could experience similar DO:DM tuning state progressions over the course of their maturation until DM activity reaches a point that promotes stable and abundant epitope presentation. In this way, a range of pMHC-II could be made available for T cell inspection over the course of APC maturation.

In conclusion, our study points to DO:DM tuning as an important factor that modulates pMHC-II repertoires. Our observations were made possible by a model system designed to recapitulate the variable levels of DO and DM seen in different kinds of APCs with different differentiation states—states that regulate the balance between vigilance against pathogens *versus* tolerance of self. Accounting for multiple DO:DM states in the context of DO regulation will guide future investigations, such as comparing pMHC-II repertoires derived from multiple HLA-II alleles with respect to varying DO:DM and from primary cells that are sorted into different DO:DM states. In addition, further exploration of scenarios in which DO expression is limited (*e.g.*, when macrophages or DO^null^ dendritic cells present antigen to T cells) is warranted, because it is likely that we have also underestimated the graded effects that varied levels of DM can have on pMHC-II presentation. Experiments like these stand to promote improved models of MHC-II antigen presentation that can be generalized to a wider array of APCs with important relevance to human health.

## Data Availability

All peptide data and mass spectrometry raw data files have been deposited in the PRIDE Archive at www.ebi.ac.uk/pride/archive under accession number PXD024392.

## Supplemental data

This article contains supplemental data.

## Conflict of interest

The authors declare no competing interests.
