# Supplementary material for: Tuning DO:DM Ratios Modulates MHC Class II Immunopeptidomes
Source: Mol Cell Proteomics. 2022 Jan 25;21(3):100204. doi: 10.1016/j.mcpro.2022.100204 (PMC10329146; doi:10.1016/j.mcpro.2022.100204)
Supplement: Supplemental Figures S1–S12 [file mmc1.pdf]

Supplementary Information for:

Tuning DO:DM ratios modulates MHC class II immunopeptidomes

Niclas Olsson[1,2†], Wei Jiang[3,4†], Lital N. Adler[3,5], Elizabeth D. Mellins[3,4††], Joshua E. Elias[6††*]

[1]Department of Chemical and Systems Biology, Stanford School of Medicine, Stanford University, Stanford, CA 94025, USA

[2]Current address: Calico LLC, South San Francisco, CA 94080, USA

[3]Department of Pediatrics – Human Gene Therapy, Stanford University School of Medicine, Stanford University, Stanford, CA 94305, USA

[4]Stanford Immunology, Stanford University School of Medicine, Stanford, CA 94305, USA

[5]Current address: Department of Biological Regulation, Weizmann Institute of Science, Rehovot, 7610001, Israel

[6]Chan Zuckerberg Biohub, Stanford, CA 94025, USA

[†, ††]These authors contributed equally to this work

*Corresponding author: Dr. Joshua E. Elias (josh.elias@czbiohub.org)

SUPPLEMENTARY TABLES

Sample	Unique peptide IDs	Unique peptide IDs (quant both bioreps.)	Cores	Cores for NetMHC
T2DR4 (biorep1)<br>T2DR4 (biorep2)	2768<br>2873	1380	476	475
T2DR4DM (biorep1)<br>T2DR4DM (biorep2)	3119<br>3349	1943	527	525
T2DR4DMDO(+) (biorep1)<br>T2DR4DMDO(+) (biorep2)	3514<br>3594	2187	607	605
T2DR4DMDO(++) (biorep1)<br>T2DR4DMDO(++) (biorep2)	3337<br>3125	1839	538	534
T2DR4DMDO(+++) (biorep1)<br>T2DR4DMDO(+++) (biorep2)	3423<br>3529	1849	658	656
T2DR4DMDO-Knockout (biorep1)<br>T2DR4DMDO-Knockout (biorep2)	1502<br>2727	799	243	242
Total # of unique peptides in dataset	10587	4528	1206*	1206*

*Total core epitopes when all cell lines analyzed together

Supplementary Table 1.

Overview of the MS-derived HLA-DR4 peptidome data, summarizing peptide and PLAtEAU-deduced core identifications. In case of multiple cores sharing an identical amino acid sequence but identified by MS to contain variable modifications (such as oxidized methionine, deamidation, cysteinylation or phosphorylation), only the sequence was included for NetMHCIIpan-4.0 analysis and enumerated once. A small number of cores containing unidentified amino acids denoted as "X" or "Z" were excluded from NetMHCIIpan-4.0 analysis.

Supplementary Table 2. 1,206 cores deduced from Plateau algorithm. See file Supplemental_table_2.xlsx

Supplementary Table 3. 729 cores with significant variance across cell lines, and associated elution data, corresponding with Figure 4C. See file Supplemental_table_3.xlsx

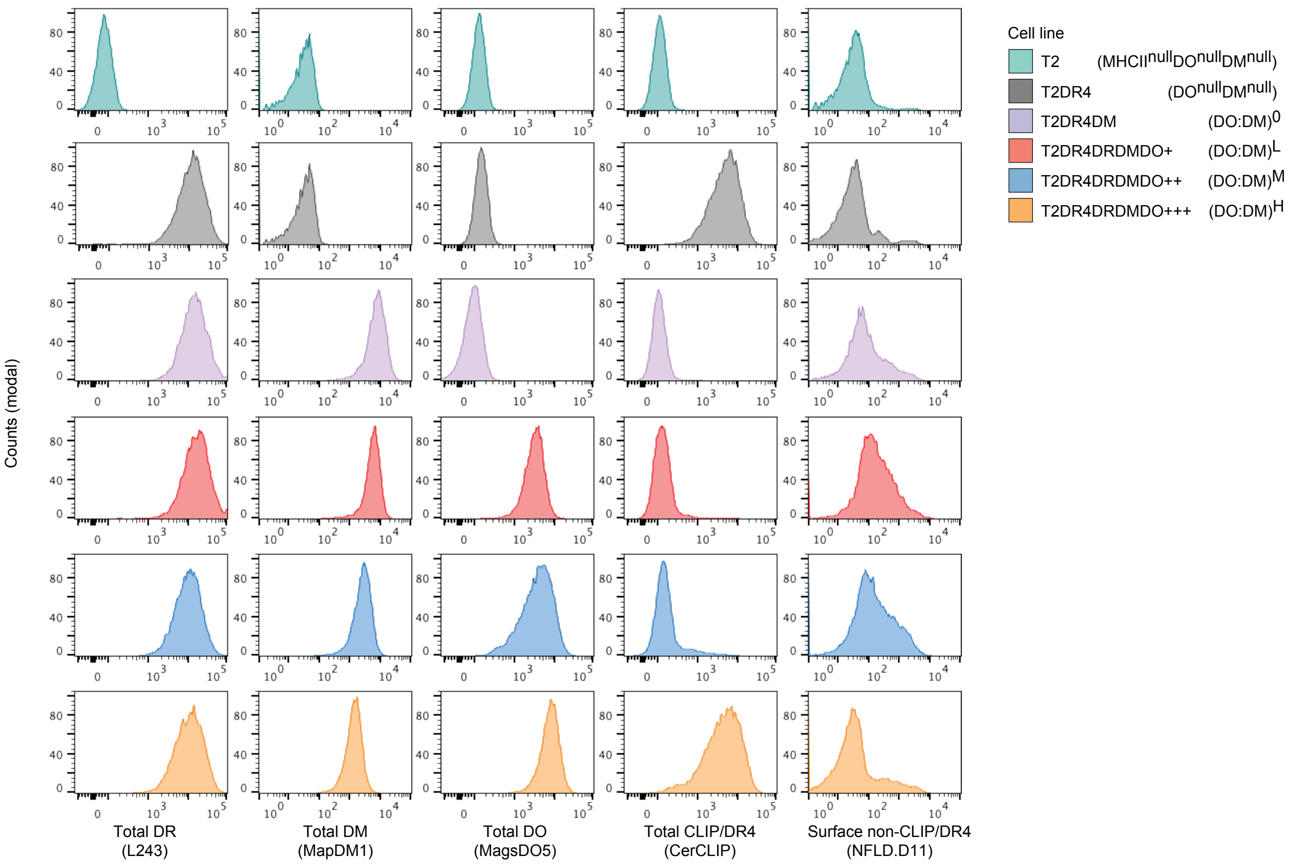

Supplementary Figure 1. Representative flow cytometric histograms showing the expression of DR, DM, DO, CLIP/DR4 complexes and surface non-CLIP/DR4 complexes in different T2-derived cell lines. Monoclonal antibodies (mAb) used for staining are L243, MapDM1, MagsDO5, CerCLIP, and NFLD.D11, as indicated. See Fig. 2B for quantification.

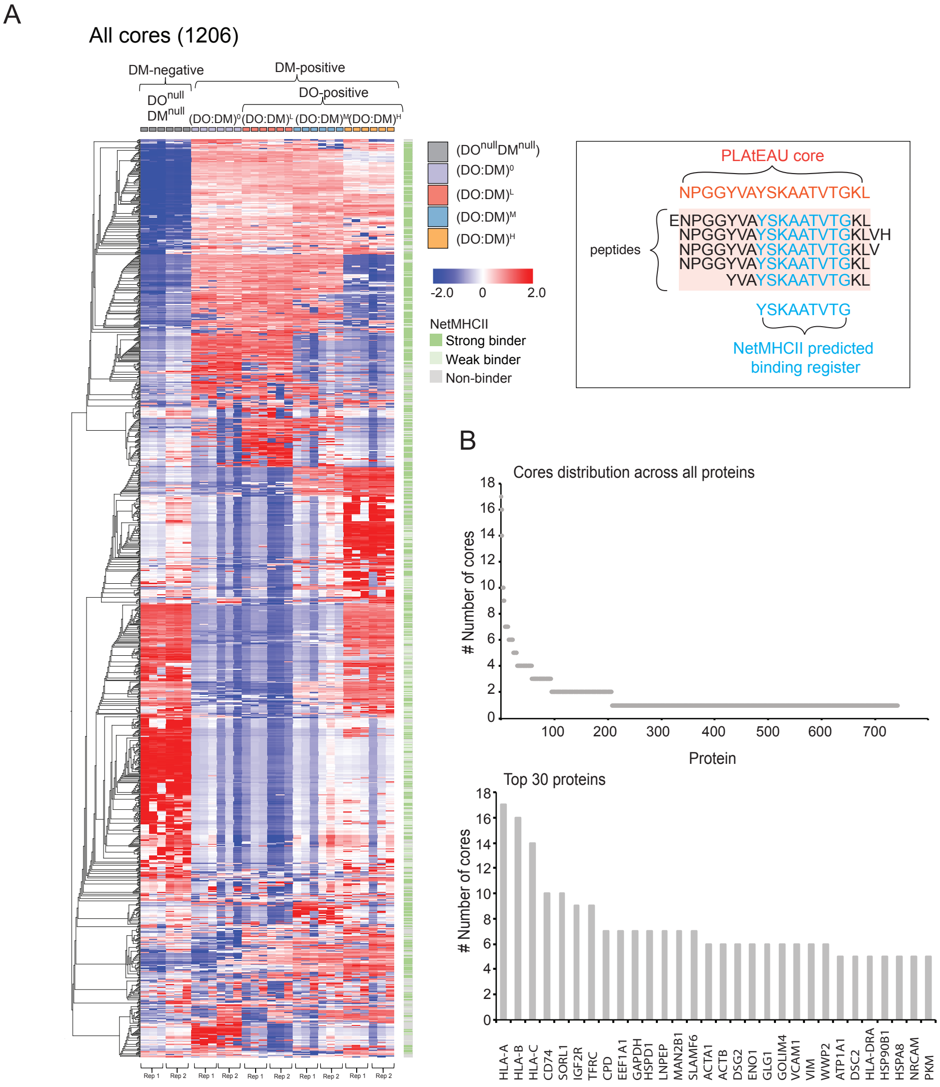

Supplementary Figure 2. Identification of cores and their distribution across proteins. (**A**) Heatmap illustrating all 1,206 cores (z-score normalized) for which we quantified the corresponding peptide in both biological replicates of any single cell line. Binding predictions to DR4 by NetMHCIIpan-4.0 are also depicted to the right as in Figure 4C. **(B)** The distribution of cores across (top) all proteins and or (bottom) the 30 indicated proteins with highest number of total cores.

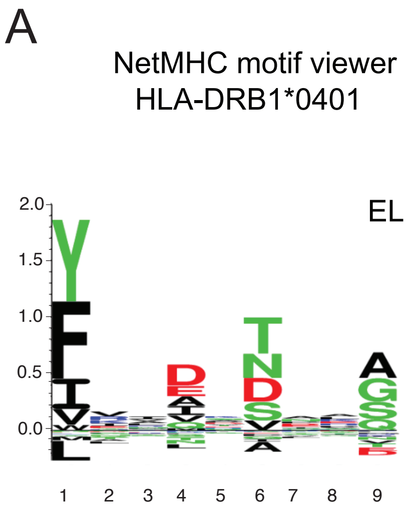

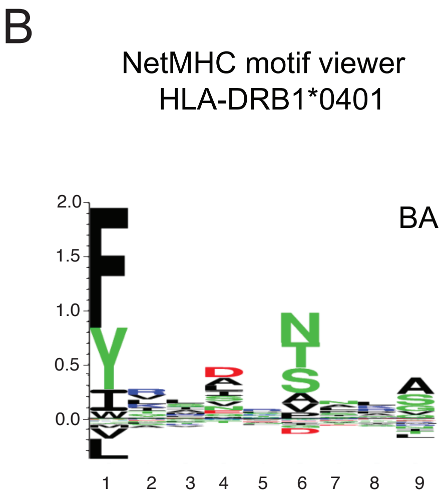

Supplementary Figure 3. DR4 motifs downloaded from the NetMHCIIpan-4.0 motif viewer (http://www.cbs.dtu.dk/services/NetMHCIIpan/logos.php). (**A**) Motif based on eluted ligand mass spectrometry (EL) data **(B)** Motif based on Binding Affinity (BA).

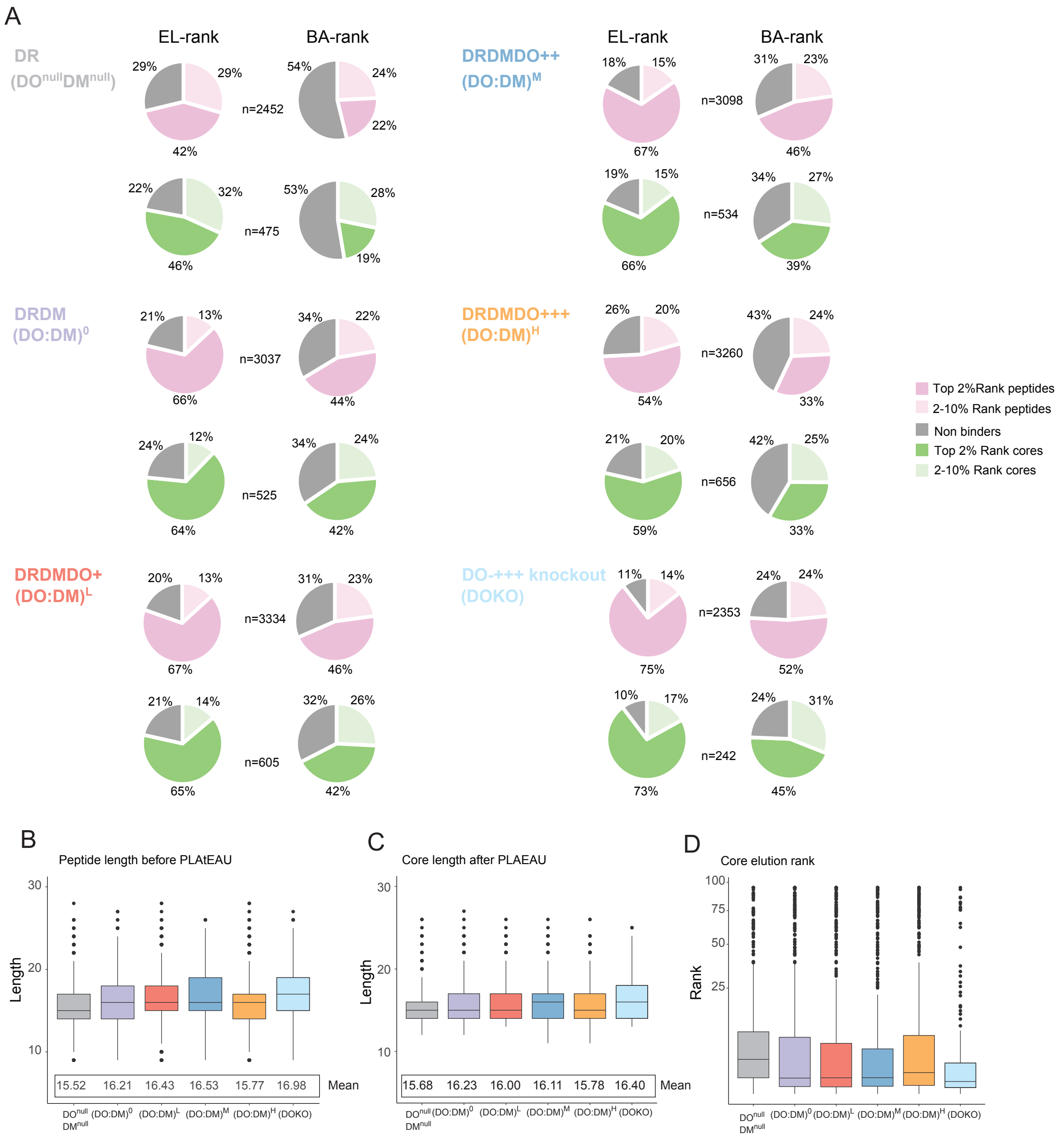

Supplementary Figure 4. Evaluation of peptide and core epitope lengths, elution rank and binding rank affinities. (A) Core epitope analysis through PLAtEAU was performed for each individual cell line, using all identified peptides per cell line. Binding affinity prediction results from NetMHC are shown for both the elution rank (EL) and the binding affinity (BA) scores. **(B)** Peptide length distribution of all identified peptides in each cell line. **(C)** Core length distributions. **(D)** Elution rank score distribution for all cores.

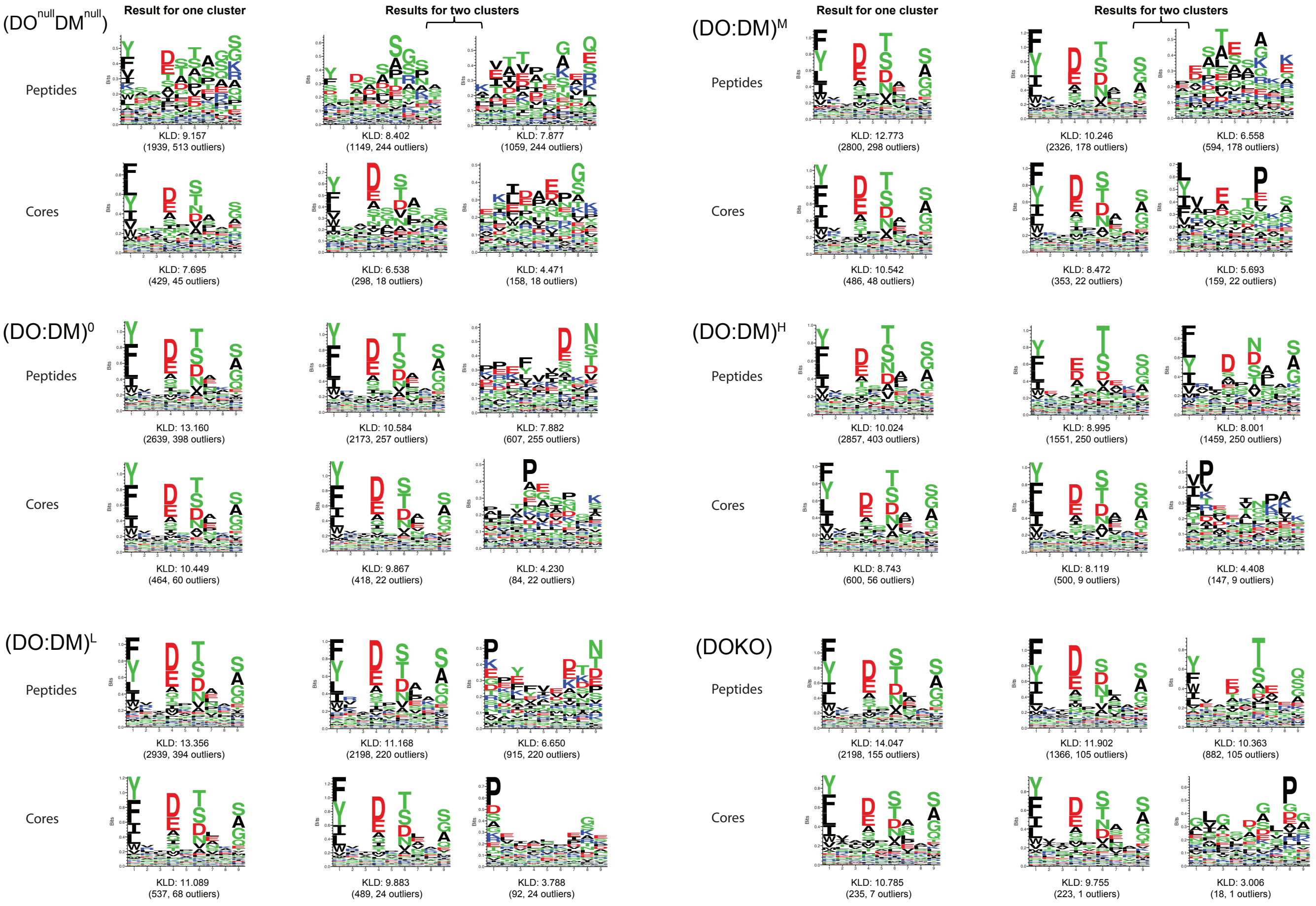

Supplementary Figure 5. Identification of DR4-like binding motifs using Gibbs cluster analysis for each individual cell line. Gibbs cluster analysis for all peptides or cores (through PLAtEAU) are shown, as illustrated using Seq2Logo. The top or top two reported clusters for each condition are presented. The Kullback-Leibler Distance (KLD) score is listed and the size of the cluster(s) and number of outliers in each cluster are listed in brackets.

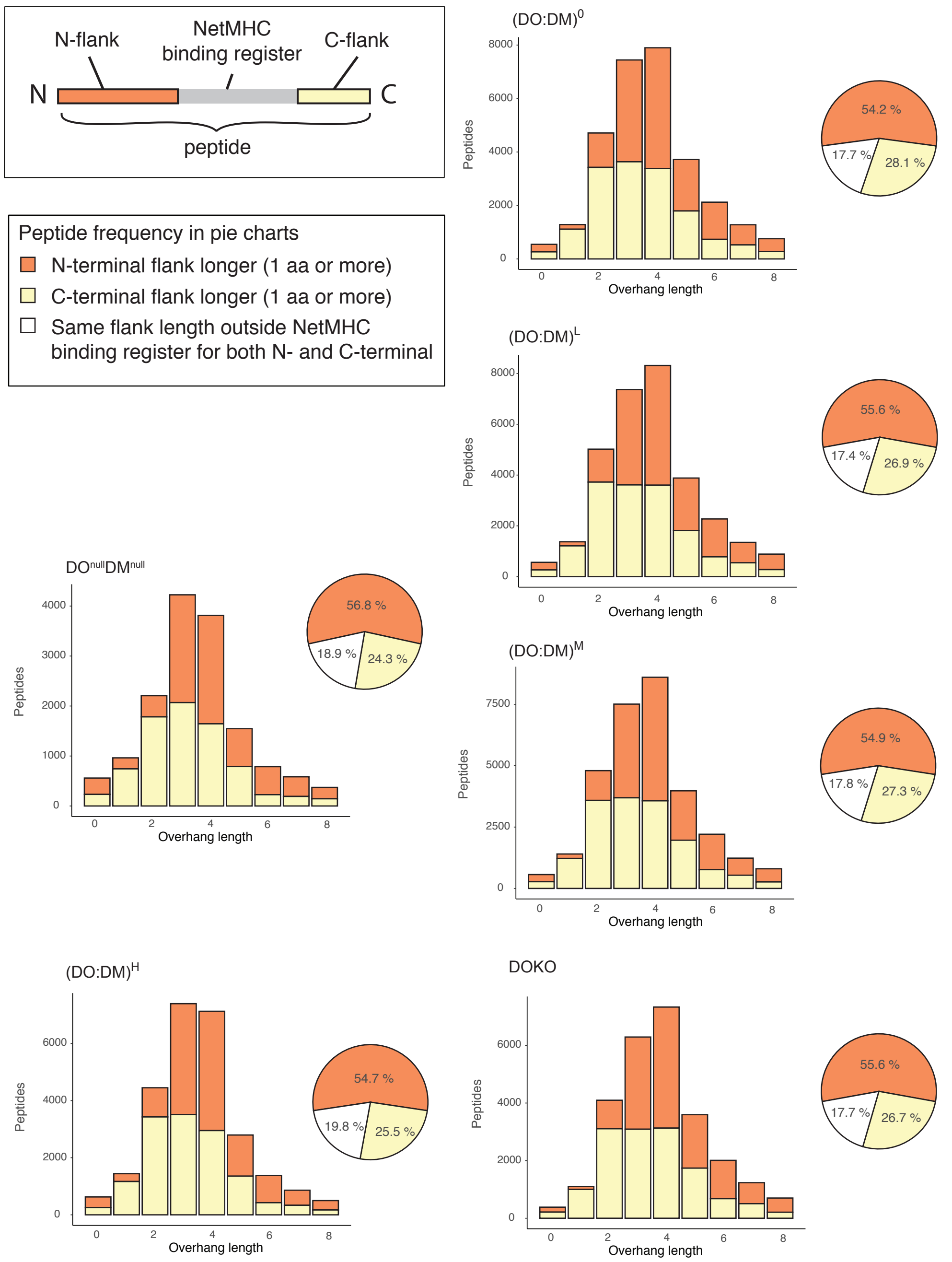

Supplementary Figure 6. HLA-DR4-presented peptides' binding registers are skewed towards their carboxyl termini, and this is not substantially influenced by DO:DM. 9-aa registers were predicted using NetMHCIIpan 4.0, as described in the methods. The number of amino acids between a peptide's N-terminus and the N-terminal start of its register (N-overhang) was tallied for all peptides identified from each cell line. Likewise, the number of amino acids between a peptide register's C-terminal end and the peptide's C-terminus was similarly tallied (C-overhang). The C-terminal skewing appeared to be exacerbated in cell lines with the lowest apparent DM activity ($DO^{null}DM^{null}$ and $(DO:DM)^{H}$) relative to those with low-to-moderate DM activity [$(DO:DM)^{0\text{-}M}$]. Whether this more C-terminal favored register positioning is directly related to DM activity or to an overall bias towards shorter peptides (Fig. 3D) is beyond the peptidomic analysis. We note, however that the $(DO:DM)^{KO}$ cells resembled $(DO:DM)^{M}$ cells' overhang length distribution more so than that of $(DO:DM)^{H}$ cells, consistent with our observations shown in Figure 6. All peptides confidently identified from each noted cell line (Supplemental Data 2) were used in this analysis.

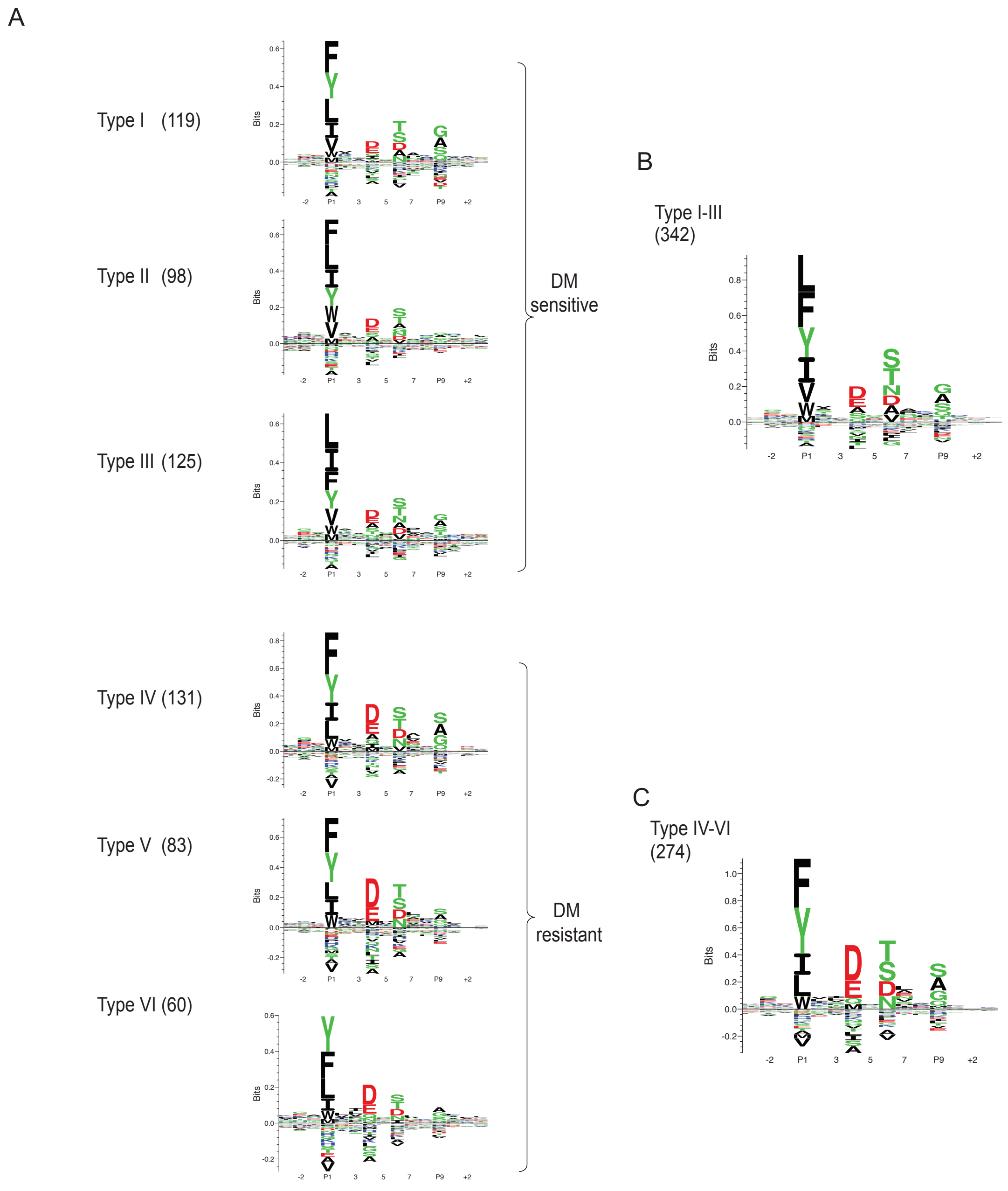

Supplementary Figure. 7. Motif analysis revealing the amino acid sequence preferences at each position within the 9-aa binding register (positions adjusted according to Fig. 4D) and at three additional N- or C-terminal flanking residue positions. Core types were inferred with the PLAtEAU algorithm and clustered as in Fig. 4C. (A) Motif analysis of the individual core types I-IV. These analyses were limited to sequences where three amino acid residues upstream of P1 and downstream of P9 were present (i.e., the nine-mer binding register was at least three residues from the protein's N- and C-terminus). In addition, a specific binding register was only allowed to be present once per core. The motifs were visualized using Seq2Logo with the Kullback-Leibler and a Hobohm1 clustering with default threshold (0.63) and weight on prior (pseudo counts) set to 200.(B) Combined flanking motif analysis of type I-III (DM-sensitive) sequences. (C) Combined flanking motif analysis of type IV-VI (DM-resistant) sequences.

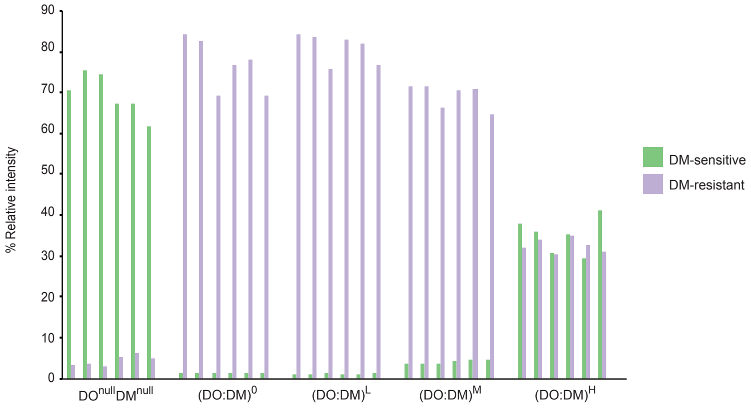

Supplementary Figure. **8**. Percent abundance subtotal of DM-sensitive (Green=Type I-III) vs DM-resistant (Purple=Type IV-VI) cores (clustered in Fig. 4C) quantified in each technical replicate of different cell lines.

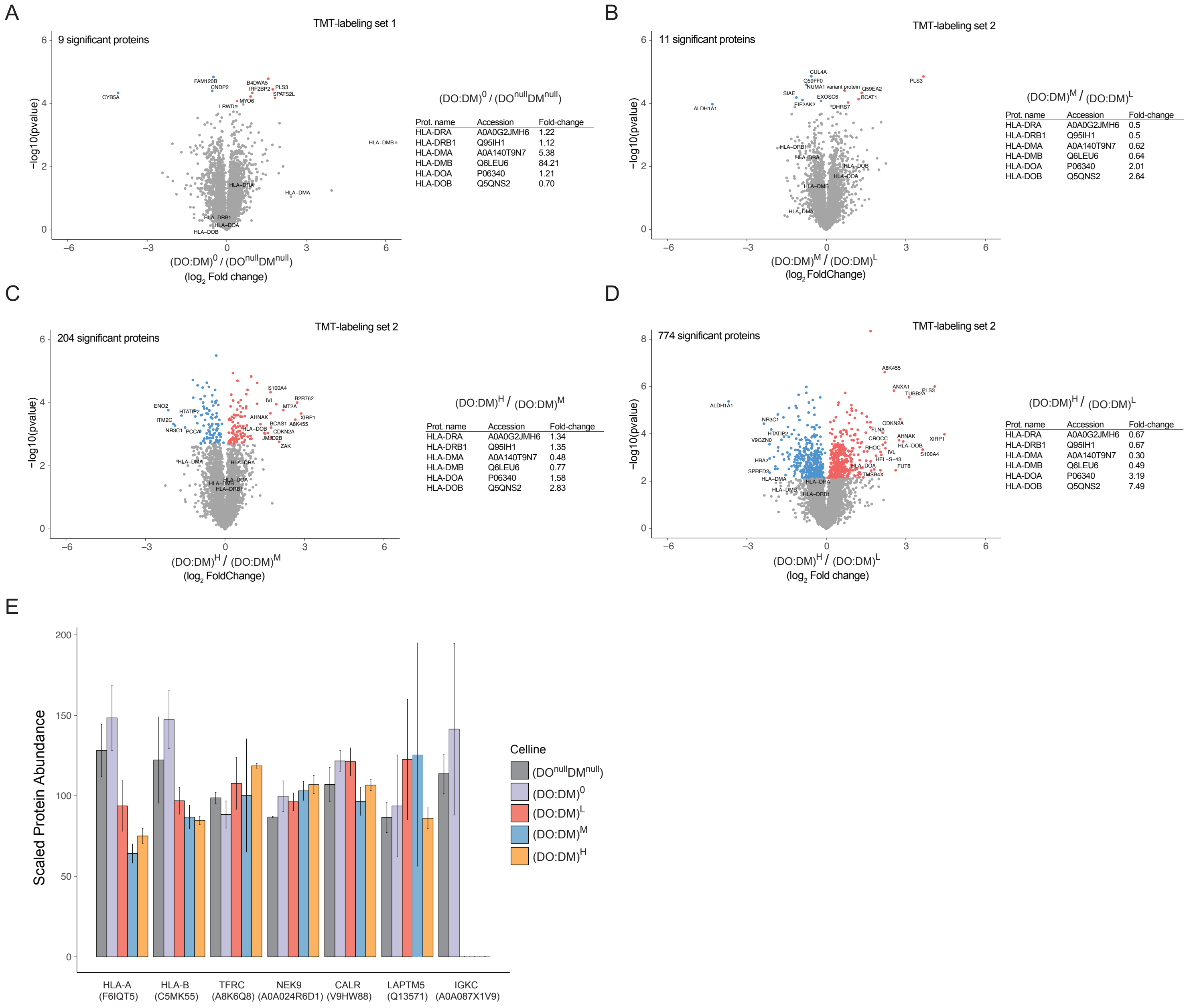

Supplementary Figure 9. Evaluation of differences in the proteomes between the DM-negative, the DM-positive and the three DO-positive lines. **(A)** Minimal proteome expression differences were observed between the $(DO{:}DM)^0$ and the $DM^{null}DO^{null}$ line. **(B)** Proteome expression differences between the $(DO{:}DM)^M$ and the $(DO{:}DM)^L$ cell line. **(C)** Proteome expression differences between the $(DO{:}DM)^H$ and the $(DO{:}DM)^M$ lines. **(D)** Proteome expression differences between the $(DO{:}DM)^H$ and the $(DO{:}DM)^L$ lines. In addition, to a subset of the most differentially expressed proteins, a selected set of proteins (including HLA-DRA, HLA-DRB1, HLA-DMA, HLA-DMB, HLA-DOA) are also called out. (A-D): Differentially expressed upregulated and downregulated proteins are indicated in red and blue, respectively (q-value of <0.05). In addition to all the differentially expressed proteins, a set of key proteins (including HLA-DRA, HLA-DRB1, HLA-DMA, HLA-DMB, HLA-DOA) are called out. **(E)** Histograms represent scaled TMT-labeled protein signals from the corresponding proteins HLA-A, HLA-B, TFRC, NEK9, CALR, LAPTM5 and IGKC ± SD. The signal was scaled through the pooled “bridge” samples used in each TMT-label set (Fig. 1).

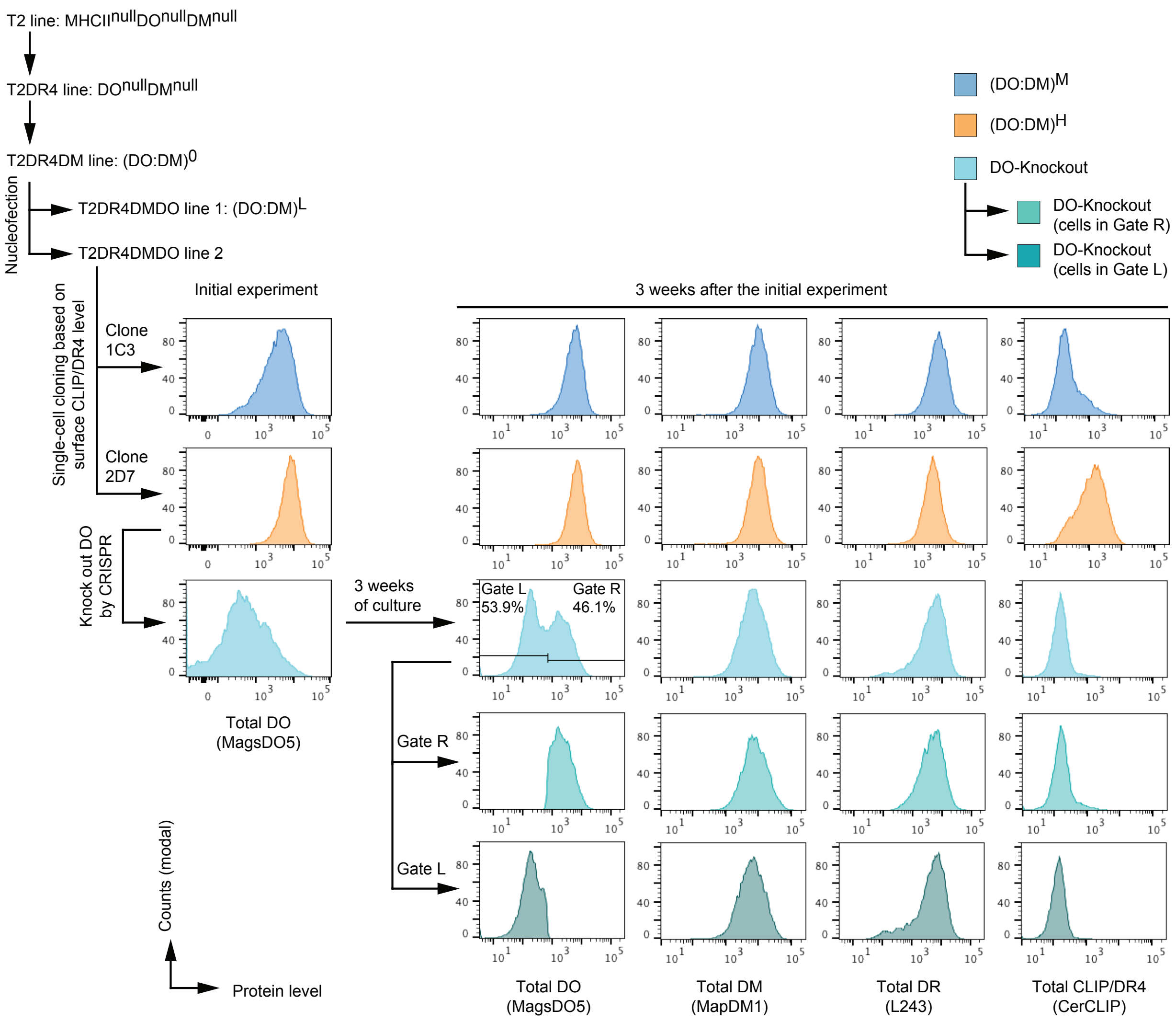

Supplementary Figure 10. Flow data for DOKO to show varied DO expression levels in DOKO subpopulations.

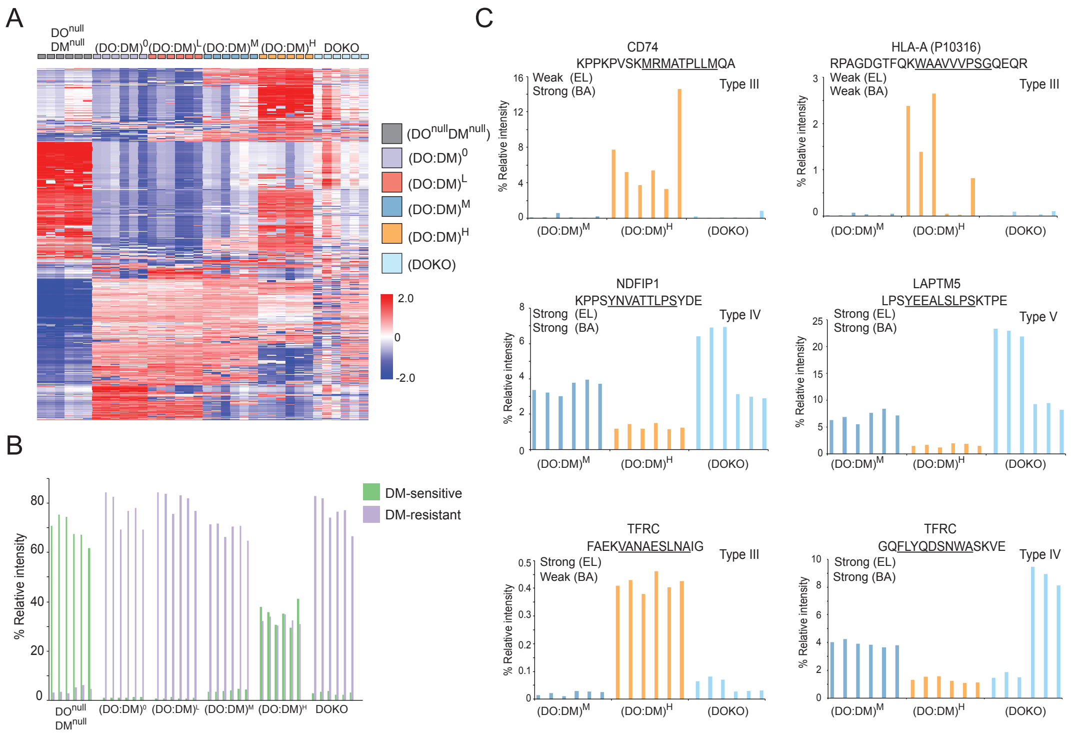

Supplementary Figure 11. Reducing the levels of DO by CRISPR knockout results in a more (DO:DM)^{L} like and typical DM-resistant phenotype. **(A)** Heatmap (z-score normalized and ordered based on the hierarchy clustering established in Fig. 4C with the addition of the DOKO line. **(B)** % abundance subtotal of DM-sensitive vs DM-resistant cores (clustered in Fig. 4C) quantified in each technical replicate of DOKO vs others. **(C)** Three examples of type III cores from CD74, HLA-A and TFRC. Two examples of Type IV cores for the NDFIP1 and TFRC protein. One example of a type V core from LAPTM5. The predicted binding register by NetMHCII is underlined.

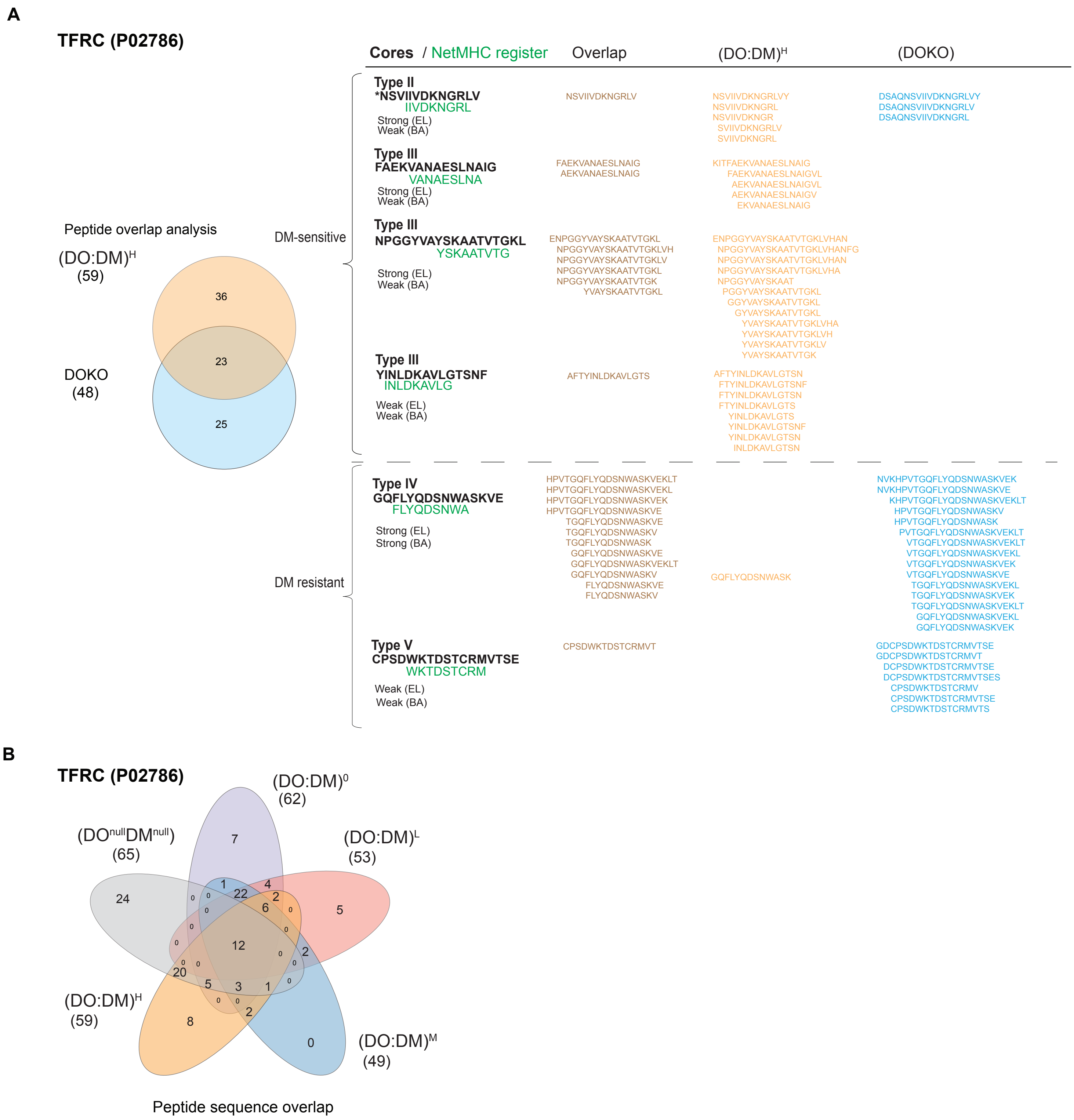

Supplementary Figure 12. Differential DO:DM influences TFRC cores and peptide presentation. (A) Direct comparison of peptides presented by the DOKO and the $(DO:DM)^H$ lines. The peptides were matched to the corresponding core types from Figure 4C. **(B)** Unique peptide sequence overlap analysis illustrated as a Venn-diagram between the five lines, excluding the DOKO.

SUPPLEMENTARY DATA

Supplementary Data 1. Peptides and proteins identified and quantified from three TMT data sets spanning T2 and six T2-derived cell lines. See file Supplemental_Data_1.xlsx

Supplementary Data 2. Annotated spectra for all single-peptide protein identifications from the proteome analysis (Supplementary Dataset 1). See file Supplemental_Data_2.pdf

Supplementary Data 3. Peptides identified and quantified from six T2-derived cell lines. See file Supplemental_Data_3.xlsx
