# Supplementary material for: Tuning DO:DM Ratios Modulates MHC Class II Immunopeptidomes
Source: Mol Cell Proteomics. 2022 Jan 25;21(3):100204. doi: 10.1016/j.mcpro.2022.100204 (PMC10329146; doi:10.1016/j.mcpro.2022.100204)

Single peptide IDs in proteome datasets

SQSPTCQMCGEK
Q12999

Xcorr: 4.00

#1	b+	b2+	Seq.	y+	y2+	#2
1	317.20224	159.10476	S-TMT6plex			12
2	445.26081	223.13405	Q	1554.69120	777.84924	11
3	532.29284	266.65006	S	1426.63262	713.81995	10
4	629.34561	315.17644	P	1339.60059	670.30394	9
5	730.39328	365.70028	T	1242.54783	621.77755	8
6	890.42393	445.71561	C-Carbami...	1141.50015	571.25371	7
7	1018.48251	509.74489	Q	981.46950	491.23839	6
8	1149.52300	575.26514	M	853.41093	427.20910	5
9	1309.55364	655.28046	C-Carbami...	722.37044	361.68886	4
10	1366.57511	683.79119	G	562.33979	281.67353	3
11	1495.61770	748.31249	E	505.31833	253.16280	2
12			K-TMT6plex	376.27574	188.64151	1

FL0008638.raw #24125 RT: 52.6782 min
ITMS, 935.9479@cid35.00, z=+2, Mono m/z=935.94794 Da, MH+=1870.88860 Da, Match Tol.=0.6 Da

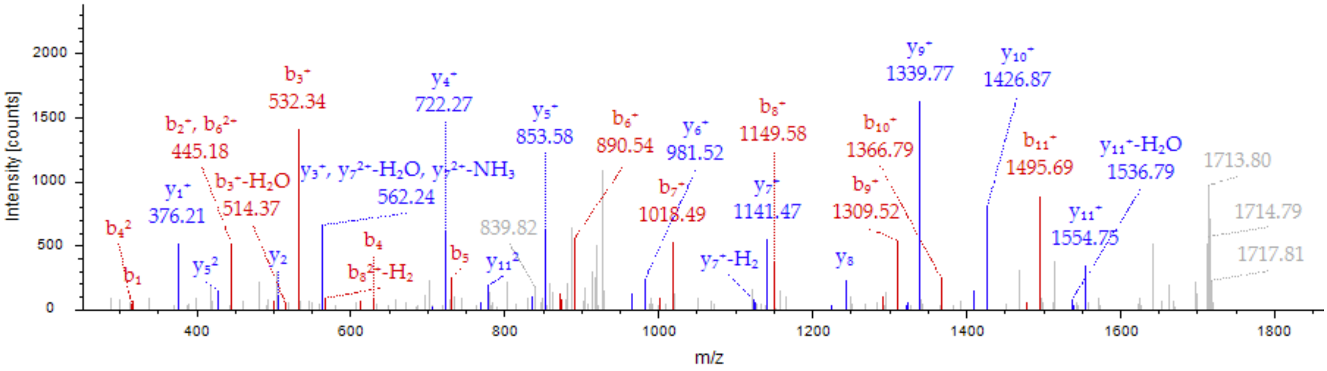

HGSVSADEAAR (A0A024R601)

Xcorr: 3.45

#1	b⁺	b²⁺	b³⁺	Seq.	y⁺	y²⁺	y³⁺	#2
1	367.22912	184.11820	123.08122	H-TMT6plex				11
2	424.25058	212.62893	142.08838	G	962.45376	481.73052	321.48944	10
3	511.28261	256.14494	171.09906	S	905.43230	453.21979	302.48228	9
4	610.35103	305.67915	204.12186	V	818.40027	409.70377	273.47161	8
5	697.38305	349.19517	233.13254	S	719.33186	360.16957	240.44880	7
6	768.42017	384.71372	256.81157	A	632.29983	316.65355	211.43813	6
7	883.44711	442.22719	295.15389	D	561.26272	281.13500	187.75909	5
8	1012.48970	506.74849	338.16809	E	446.23577	223.62152	149.41678	4
9	1083.52682	542.26705	361.84712	A	317.19318	159.10023	106.40258	3
10	1154.56393	577.78560	385.52616	A	246.15607	123.58167	82.72354	2
11				R	175.11895	88.06311	59.04450	1

FL0008608.raw #10303 RT: 26.9889 min
ITMS, 443.5629@cid35.00, z=+3, Mono m/z=443.56293 Da, MH+=1328.67423 Da, Match Tol.=0.6 Da

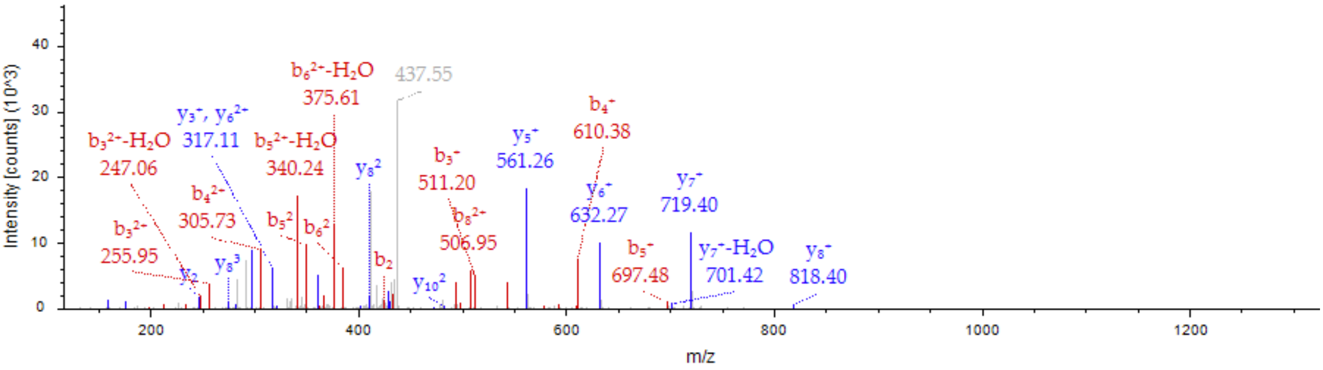

DALSSVQESQVAQQAR (B0YIW2)

Xcorr: 5.8

#1	b⁺	b²⁺	Seq.	y⁺	y²⁺	#2
1	345.19715	173.10221	D-TMT6plex			16
2	416.23427	208.62077	A	1601.82417	801.41572	15
3	529.31833	265.16280	L	1530.78706	765.89717	14
4	616.35036	308.67882	S	1417.70300	709.35514	13
5	703.38239	352.19483	S	1330.67097	665.83912	12
6	802.45080	401.72904	V	1243.63894	622.32311	11
7	930.50938	465.75833	Q	1144.57052	572.78890	10
8	1059.55197	530.27962	E	1016.51195	508.75961	9
9	1146.58400	573.79564	S	887.46935	444.23832	8
10	1274.64258	637.82493	Q	800.43733	400.72230	7
11	1373.71099	687.35913	V	672.37875	336.69301	6
12	1444.74810	722.87769	A	573.31033	287.15881	5
13	1572.80668	786.90698	Q	502.27322	251.64025	4
14	1700.86526	850.93627	Q	374.21464	187.61096	3
15	1771.90237	886.45482	A	246.15607	123.58167	2
16			R	175.11895	88.06311	1

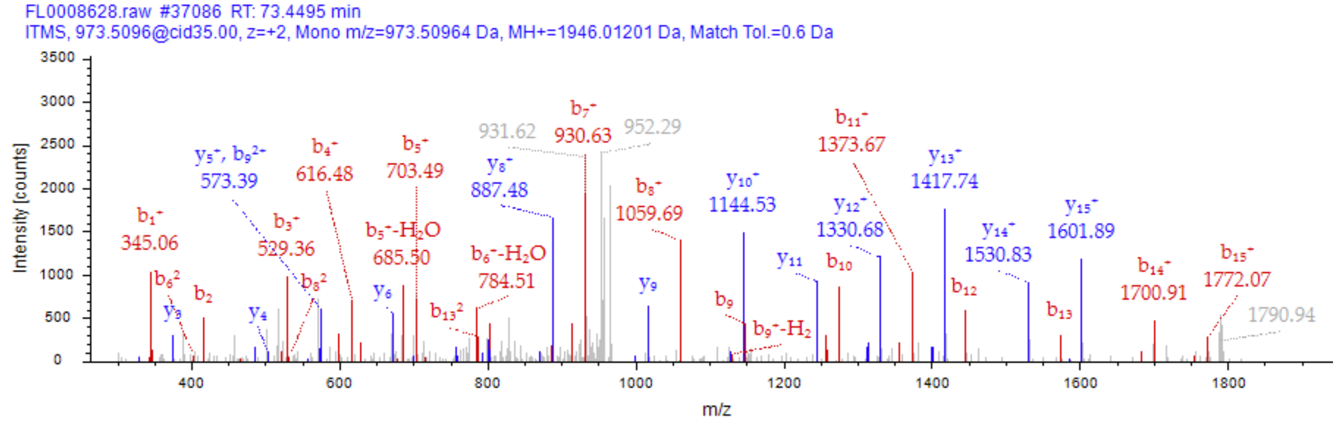

IVTDALPSALTGGEGFQD (I3L3P1)

Xcorr: 5.1

#1	b⁺	b²⁺	Seq.	y⁺	y²⁺	#2
1	343.25427	172.13077	I-TMT6plex			18
2	442.32269	221.66498	V	1677.79662	839.40195	17
3	543.37036	272.18882	T	1578.72821	789.86774	16
4	658.39731	329.70229	D	1477.68053	739.34390	15
5	729.43442	365.22085	A	1362.65359	681.83043	14
6	842.51849	421.76288	L	1291.61647	646.31187	13
7	939.57125	470.28926	P	1178.53241	589.76984	12
8	1026.60328	513.80528	S	1081.47964	541.24346	11
9	1097.64039	549.32383	A	994.44762	497.72745	10
10	1210.72446	605.86587	L	923.41050	462.20889	9
11	1311.77213	656.38971	T	810.32644	405.66686	8
12	1368.79360	684.90044	G	709.27876	355.14302	7
13	1425.81506	713.41117	G	652.25730	326.63229	6
14	1554.85765	777.93247	E	595.23583	298.12155	5
15	1611.87912	806.44320	G	466.19324	233.60026	4
16	1758.94753	879.97740	F	409.17178	205.08953	3
17	1887.00611	944.00669	Q	262.10336	131.55532	2
18			D	134.04478	67.52603	1

FL0008562.raw #78122 RT: 143.6425 min
ITMS, 1010.5226@cid35.00, z=+2, Mono m/z=1010.52258 Da, MH+=2020.03789 Da, Match Tol.=0.6 Da

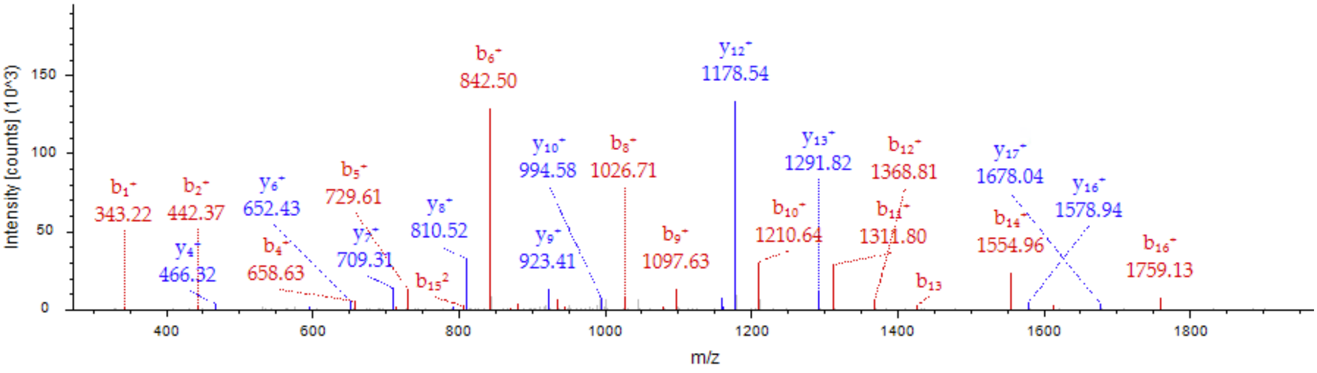

NLSDIDLMAPQPGV (Q96B49)

Xcorr: 3.89

#1	b^+	b^{2+}	Seq.	y^+	y^{2+}	#2
1	344.21314	172.61021	N-TMT6plex			14
2	457.29720	229.15224	L	1355.68753	678.34740	13
3	544.32923	272.66825	S	1242.60346	621.80537	12
4	659.35617	330.18172	D	1155.57144	578.28936	11
5	772.44024	386.72376	I	1040.54449	520.77588	10
6	887.46718	444.23723	D	927.46043	464.23385	9
7	1000.55124	500.77926	L	812.43349	406.72038	8
8	1131.59173	566.29950	M	699.34942	350.17835	7
9	1202.62884	601.81806	A	568.30894	284.65811	6
10	1299.68160	650.34444	P	497.27182	249.13955	5
11	1427.74018	714.37373	Q	400.21906	200.61317	4
12	1524.79295	762.90011	P	272.16048	136.58388	3
13	1581.81441	791.41084	G	175.10772	88.05750	2
14			V	118.08626	59.54677	1

FL0008614.raw #70677 RT: 133.5511 min
ITMS, 849.9503@cid35.00, z=+2, Mono m/z=849.95026 Da, MH+=1698.89324 Da, Match Tol.=0.6 Da

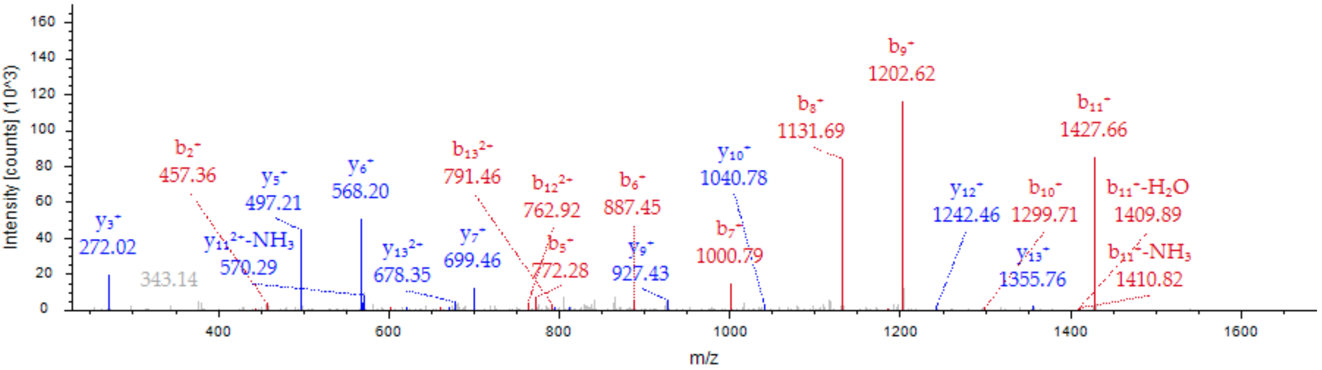

EQLGPVTQEFWDNLEK (A0A024R3E3)

Xcorr: 6.18

#1	b⁺	b²⁺	Seq.	y⁺	y²⁺	#2
1	359.21280	180.11004	E-TMT6plex			16
2	487.27138	244.13933	Q	2033.05412	1017.03070	15
3	600.35544	300.68136	L	1904.99554	953.00141	14
4	657.37691	329.19209	G	1791.91148	896.45938	13
5	754.42967	377.71847	P	1734.89002	867.94865	12
6	853.49808	427.25268	V	1637.83725	819.42226	11
7	954.54576	477.77652	T	1538.76884	769.88806	10
8	1082.60434	541.80581	Q	1437.72116	719.36422	9
9	1211.64693	606.32710	E	1309.66258	655.33493	8
10	1358.71535	679.86131	F	1180.61999	590.81363	7
11	1544.79466	772.90097	W	1033.55158	517.27943	6
12	1659.82160	830.41444	D	847.47226	424.23977	5
13	1773.86453	887.43590	N	732.44532	366.72630	4
14	1886.94859	943.97794	L	618.40239	309.70483	3
15	2015.99119	1008.49923	E	505.31833	253.16280	2
16			K-TMT6plex	376.27574	188.64151	1

FL0008566.raw #79155 RT: 144.6197 min
ITMS, 1196.6331@cid35.00, z=+2, Mono m/z=1196.13135 Da, MH+=2391.25542 Da, Match Tol.=0.6 Da

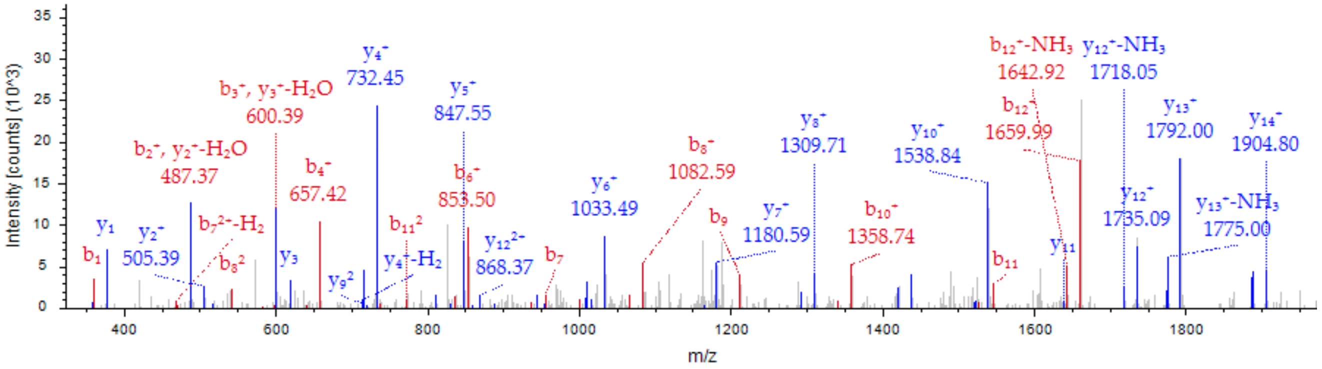

NQVIAPLTEELVFR (Q0P5W4)

Xcorr: 4.13

#1	b^+	b^{2+}	b^{3+}	Seq.	y^+	y^{2+}	y^{3+}	#2
1	344.21314	172.61021	115.40923	N-TMT6plex				14
2	472.27171	236.63950	158.09542	Q	1514.85771	757.93249	505.62409	13
3	571.34013	286.17370	191.11823	V	1386.79913	693.90320	462.93789	12
4	684.42419	342.71573	228.81291	I	1287.73071	644.36900	429.91509	11
5	755.46131	378.23429	252.49195	A	1174.64665	587.82696	392.22040	10
6	852.51407	426.76067	284.84287	P	1103.60954	552.30841	368.54136	9
7	965.59813	483.30270	322.53756	L	1006.55677	503.78202	336.19044	8
8	1066.64581	533.82654	356.22012	T	893.47271	447.23999	298.49575	7
9	1195.68840	598.34784	399.23432	E	792.42503	396.71615	264.81319	6
10	1324.73100	662.86914	442.24852	E	663.38244	332.19486	221.79900	5
11	1437.81506	719.41117	479.94320	L	534.33984	267.67356	178.78480	4
12	1536.88347	768.94538	512.96601	V	421.25578	211.13153	141.09011	3
13	1683.95189	842.47958	561.98881	F	322.18737	161.59732	108.06731	2
14				R	175.11895	88.06311	59.04450	1

FL0008572.raw #75624 RT: 138.7007 min
ITMS, 620.3590@cid35.00, z=+3, Mono m/z=620.02429 Da, MH+=1858.05832 Da, Match Tol.=0.6 Da

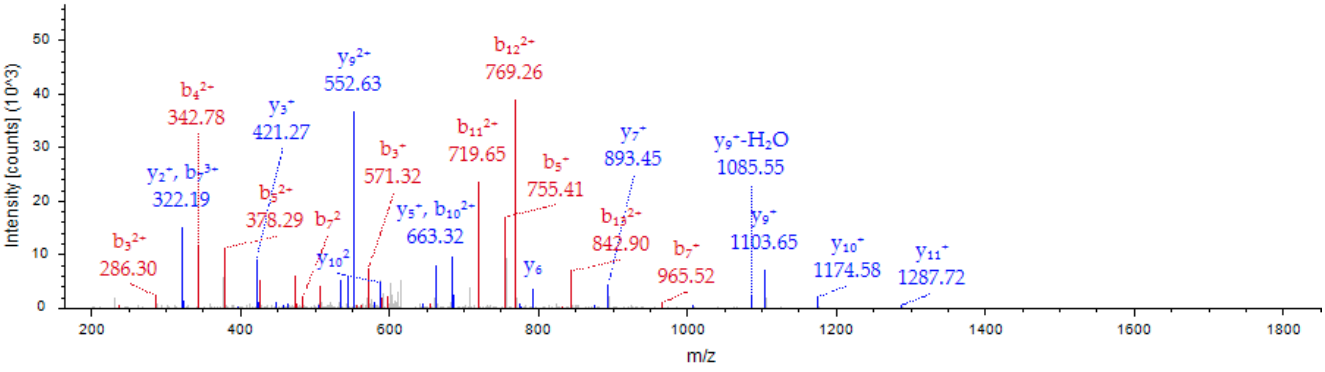

QQSEEDLLLQDFSR (Q9UNL2)

Xcorr: 5.66

#1	b⁺	b²⁺	Seq.	y⁺	y²⁺	#2
1	358.22879	179.61803	Q-TMT6plex			14
2	486.28736	243.64732	Q	1579.75984	790.38356	13
3	573.31939	287.16333	S	1451.70126	726.35427	12
4	702.36198	351.68463	E	1364.66924	682.83826	11
5	831.40458	416.20593	E	1235.62664	618.31696	10
6	946.43152	473.71940	D	1106.58405	553.79566	9
7	1059.51558	530.26143	L	991.55711	496.28219	8
8	1172.59965	586.80346	L	878.47304	439.74016	7
9	1285.68371	643.34549	L	765.38898	383.19813	6
10	1413.74229	707.37478	Q	652.30491	326.65610	5
11	1528.76923	764.88825	D	524.24634	262.62681	4
12	1675.83765	838.42246	F	409.21939	205.11334	3
13	1762.86968	881.93848	S	262.15098	131.57913	2
14			R	175.11895	88.06311	1

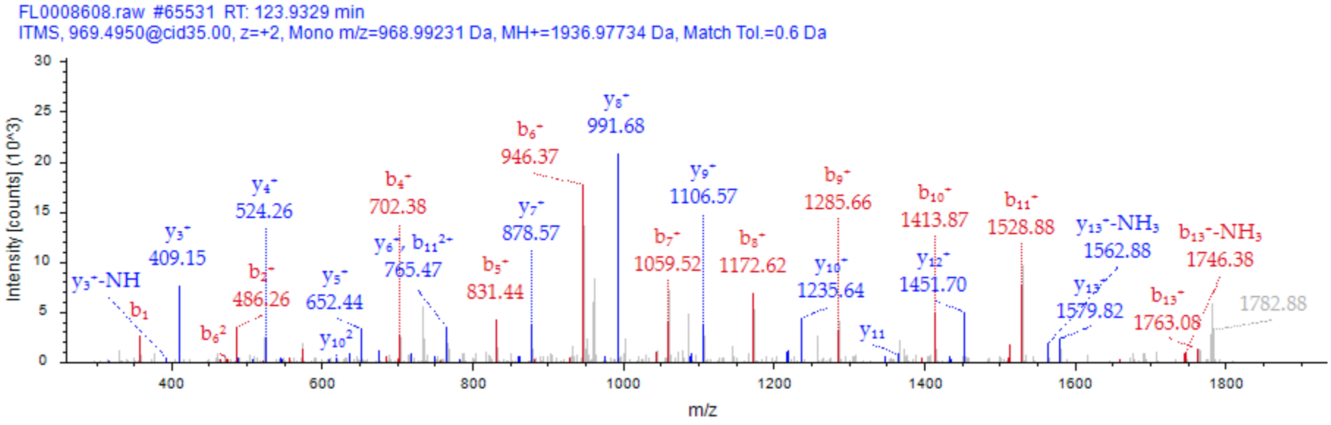

TATNNASSSCQSTPR (Q92833)

Xcorr: 4.39

#1	b⁺	b²⁺	Seq.	y⁺	y²⁺	#2
1	331.21789	166.11258	T-TMT6plex			15
2	402.25500	201.63114	A	1480.64449	740.82589	14
3	503.30268	252.15498	T	1409.60738	705.30733	13
4	617.34561	309.17644	N	1308.55970	654.78349	12
5	731.38853	366.19791	N	1194.51677	597.76203	11
6	802.42565	401.71646	A	1080.47385	540.74056	10
7	889.45768	445.23248	S	1009.43673	505.22201	9
8	976.48970	488.74849	S	922.40471	461.70599	8
9	1063.52173	532.26450	S	835.37268	418.18998	7
10	1223.55238	612.27983	C-Carbami...	748.34065	374.67396	6
11	1351.61096	676.30912	Q	588.31000	294.65864	5
12	1438.64299	719.82513	S	460.25142	230.62935	4
13	1539.69067	770.34897	T	373.21939	187.11334	3
14	1636.74343	818.87535	P	272.17172	136.58950	2
15			R	175.11895	88.06311	1

FL0008606.raw #11836 RT: 30.1195 min
ITMS, 905.9292@cid35.00, z=+2, Mono m/z=905.92920 Da, MH+=1810.85112 Da, Match Tol.=0.6 Da

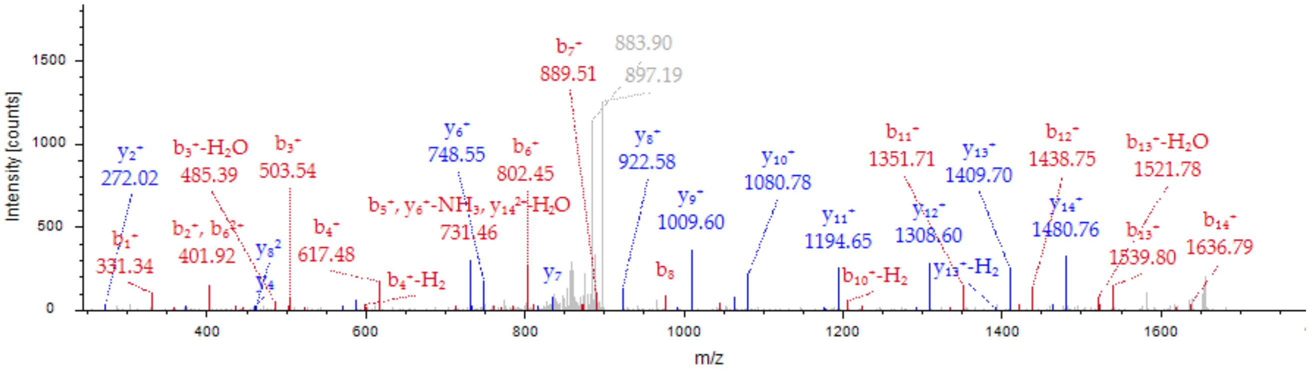

YIMVPSGNMGVFDPTEIHNR (Q53HE6)

Xcorr: 5.29

#1	b⁺	b²⁺	b³⁺	Seq.	y⁺	y²⁺	y³⁺	#2
1	393.23354	197.12041	131.74936	Y-TMT6plex				20
2	506.31760	253.66244	169.44405	I	2114.01576	1057.51152	705.34344	19
3	637.35809	319.18268	213.12421	M	2000.93169	1000.96948	667.64875	18
4	736.42650	368.71689	246.14702	V	1869.89121	935.44924	623.96859	17
5	833.47926	417.24327	278.49794	P	1770.82279	885.91503	590.94578	16
6	920.51129	460.75928	307.50861	S	1673.77003	837.38865	558.59486	15
7	977.53276	489.27002	326.51577	G	1586.73800	793.87264	529.58418	14
8	1091.57568	546.29148	364.53008	N	1529.71654	765.36191	510.57703	13
9	1222.61617	611.81172	408.21024	M	1415.67361	708.34044	472.56272	12
10	1279.63763	640.32245	427.21739	G	1284.63312	642.82020	428.88256	11
11	1378.70604	689.85666	460.24020	V	1227.61166	614.30947	409.87540	10
12	1525.77446	763.39087	509.26300	F	1128.54325	564.77526	376.85260	9
13	1640.80140	820.90434	547.60532	D	981.47483	491.24106	327.82980	8
14	1737.85417	869.43072	579.95624	P	866.44789	433.72758	289.48748	7
15	1838.90184	919.95456	613.63880	T	769.39513	385.20120	257.13656	6
16	1967.94444	984.47586	656.65300	E	668.34745	334.67736	223.45400	5
17	2081.02850	1041.01789	694.34768	I	539.30486	270.15607	180.43980	4
18	2218.08741	1109.54734	740.03399	H	426.22079	213.61403	142.74511	3
19	2332.13034	1166.56881	778.04830	N	289.16188	145.08458	97.05881	2
20				R	175.11895	88.06311	59.04450	1

FL0008588.raw #68898 RT: 126.9334 min
ITMS, 836.4179@cid35.00, z=+3, Mono m/z=836.08270 Da, MH+=2506.23355 Da, Match Tol.=0.6 Da

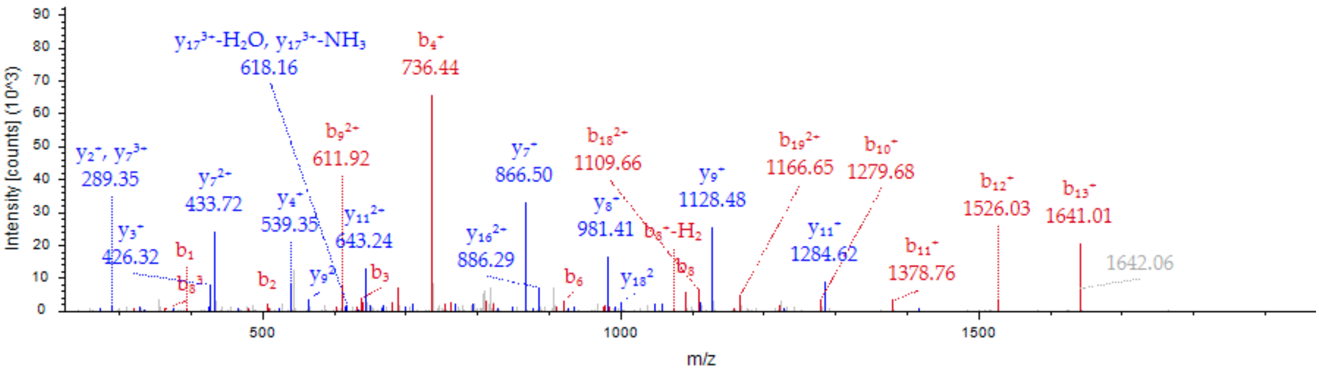

QEEACQCQCACR (Q14872)

Xcorr: 3.96

#1	b⁺	b^{2+}	b^{3+}	Seq.	y⁺	y^{2+}	y^{3+}	#2
1	358.22879	179.61803	120.08111	Q-TMT6plex				12
2	487.27138	244.13933	163.09531	E	1471.51811	736.26270	491.17756	11
3	616.31397	308.66062	206.10951	E	1342.47552	671.74140	448.16336	10
4	687.35109	344.17918	229.78855	A	1213.43293	607.22010	405.14916	9
5	847.38173	424.19451	283.13210	C-Carbami...	1142.39581	571.70155	381.47012	8
6	975.44031	488.22379	325.81829	Q	982.36517	491.68622	328.12657	7
7	1135.47096	568.23912	379.16184	C-Carbami...	854.30659	427.65693	285.44038	6
8	1263.52954	632.26841	421.84803	Q	694.27594	347.64161	232.09683	5
9	1423.56019	712.28373	475.19158	C-Carbami...	566.21736	283.61232	189.41064	4
10	1494.59730	747.80229	498.87062	A	406.18671	203.59700	136.06709	3
11	1654.62795	827.81761	552.21417	C-Carbami...	335.14960	168.07844	112.38805	2
12				R	175.11895	88.06311	59.04450	1

FL0008628.raw #13390 RT: 32.5706 min
ITMS, 610.5855@cid35.00, z=+3, Mono m/z=610.25085 Da, MH+=1828.73801 Da, Match Tol.=0.6 Da

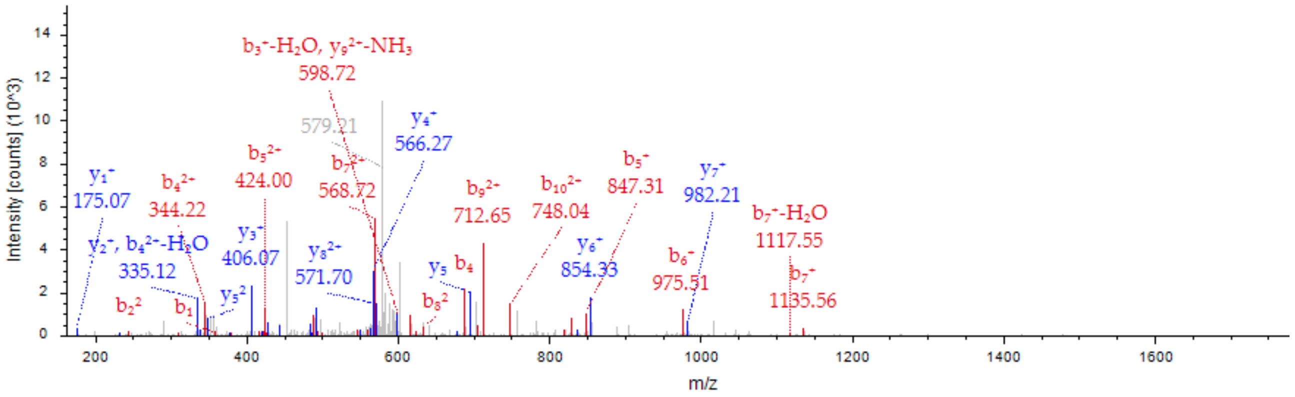

GPNVVGPYGLLQPFADAMK (A0A023Q875)

Xcorr: 4.79

#1	b⁺	b²⁺	b³⁺	Seq.	y⁺	y²⁺	y³⁺	#2
1	287.19167	144.09947	96.40208	G-TMT6plex				19
2	384.24444	192.62586	128.75300	P	2146.15681	1073.58204	716.05712	18
3	498.28736	249.64732	166.76731	N	2049.10405	1025.05566	683.70620	17
4	597.35578	299.18153	199.79011	V	1935.06112	968.03420	645.69189	16
5	696.42419	348.71573	232.81291	V	1835.99271	918.49999	612.66909	15
6	753.44565	377.22647	251.82007	G	1736.92429	868.96579	579.64628	14
7	850.49842	425.75285	284.17099	P	1679.90283	840.45505	560.63913	13
8	1013.56175	507.28451	338.52543	Y	1582.85007	791.92867	528.28821	12
9	1070.58321	535.79524	357.53259	G	1419.78674	710.39701	473.93376	11
10	1183.66727	592.33728	395.22728	L	1362.76527	681.88628	454.92661	10
11	1296.75134	648.87931	432.92196	L	1249.68121	625.34424	417.23192	9
12	1424.80992	712.90860	475.60816	Q	1136.59715	568.80221	379.53723	8
13	1521.86268	761.43498	507.95908	P	1008.53857	504.77292	336.85104	7
14	1668.93109	834.96919	556.98188	F	911.48581	456.24654	304.50012	6
15	1739.96821	870.48774	580.66092	A	764.41739	382.71233	255.47731	5
16	1854.99515	928.00121	619.00323	D	693.38028	347.19378	231.79828	4
17	1926.03226	963.51977	642.68227	A	578.35333	289.68031	193.45596	3
18	2057.07275	1029.04001	686.36243	M	507.31622	254.16175	169.77692	2
19				K-TMT6plex	376.27574	188.64151	126.09676	1

FL0008620.raw #80231 RT: 146.8239 min
ITMS, 811.7859@cid35.00, z=+3, Mono m/z=811.45197 Da, MH+=2432.34134 Da, Match Tol.=0.6 Da

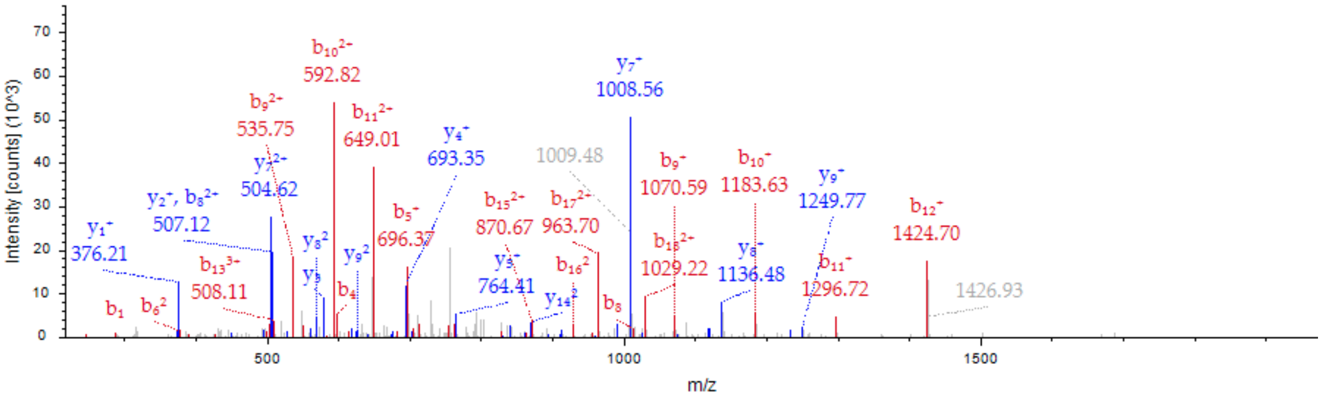

GGYGGGMPANVQMQLVDTK (Q9NRR3)

Xcorr: 5.71

#1	b⁺	b²⁺	Seq.	y⁺	y²⁺	#2
1	287.19167	144.09947	G-TMT6plex			19
2	344.21314	172.61021	G	2095.05136	1048.02932	18
3	507.27646	254.14187	Y	2038.02990	1019.51859	17
4	564.29793	282.65260	G	1874.96657	937.98692	16
5	621.31939	311.16333	G	1817.94511	909.47619	15
6	678.34086	339.67407	G	1760.92364	880.96546	14
7	809.38134	405.19431	M	1703.90218	852.45473	13
8	906.43410	453.72069	P	1572.86169	786.93449	12
9	977.47122	489.23925	A	1475.80893	738.40810	11
10	1091.51415	546.26071	N	1404.77182	702.88955	10
11	1190.58256	595.79492	V	1290.72889	645.86808	9
12	1318.64114	659.82421	Q	1191.66047	596.33388	8
13	1449.68162	725.34445	M	1063.60190	532.30459	7
14	1577.74020	789.37374	Q	932.56141	466.78434	6
15	1690.82426	845.91577	L	804.50284	402.75506	5
16	1789.89268	895.44998	V	691.41877	346.21302	4
17	1904.91962	952.96345	D	592.35036	296.67882	3
18	2005.96730	1003.48729	T	477.32341	239.16535	2
19			K-TMT6plex	376.27574	188.64151	1

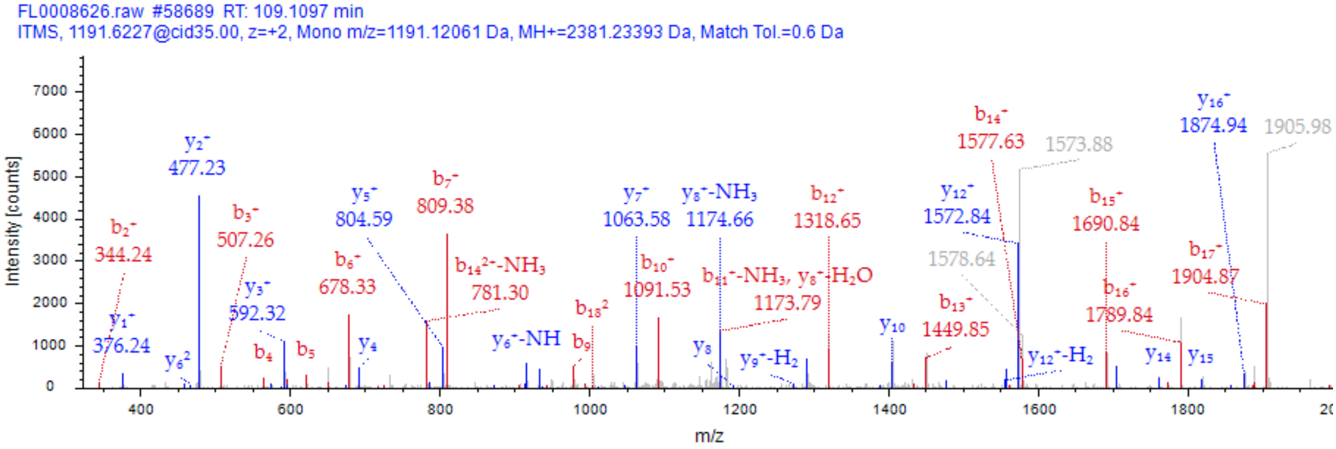

SVIEDVINDVR (P52655)

Xcorr: 3.58

#1	b+	b2+	b3+	Seq.	y+	y2+	y3+	#2
1	317.20224	159.10476	106.40560	S-TMT6plex				11
2	416.27065	208.63896	139.42840	V	1171.63173	586.31950	391.21543	10
3	529.35471	265.18100	177.12309	I	1072.56331	536.78530	358.19262	9
4	658.39731	329.70229	220.13729	E	959.47925	480.24326	320.49793	8
5	773.42425	387.21576	258.47960	D	830.43666	415.72197	277.48374	7
6	872.49266	436.74997	291.50241	V	715.40971	358.20850	239.14142	6
7	985.57673	493.29200	329.19709	I	616.34130	308.67429	206.11862	5
8	1099.61966	550.31347	367.21140	N	503.25724	252.13226	168.42393	4
9	1214.64660	607.82694	405.55372	D	389.21431	195.11079	130.40962	3
10	1313.71501	657.36114	438.57652	V	274.18737	137.59732	92.06731	2
11				R	175.11895	88.06311	59.04450	1

FL0016322.raw #73152 RT: 141.8675 min
ITMS, 496.9470@cid35.00, z=+3, Mono m/z=496.61328 Da, MH+=1487.82529 Da, Match Tol.=0.6 Da

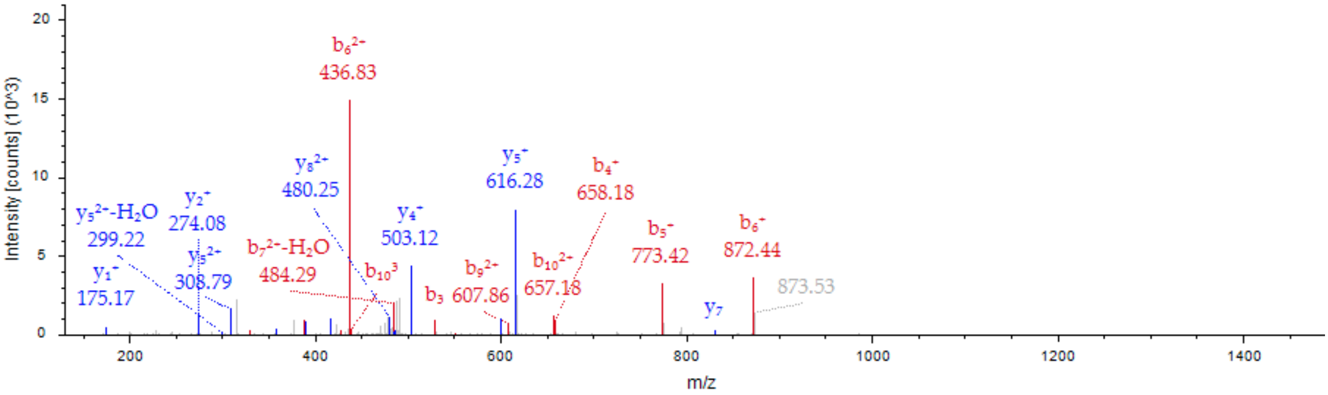

ISDEILSDTIGEK

Xcorr: 4.41

(Q9NVA4)

#1	b⁺	b²⁺	Seq.	y⁺	y²⁺	#2
1	343.25427	172.13077	I-TMT6plex			13
2	430.28630	215.64679	S	1535.80020	768.40374	12
3	545.31324	273.16026	D	1448.76817	724.88772	11
4	674.35584	337.68156	E	1333.74123	667.37425	10
5	787.43990	394.22359	I	1204.69863	602.85296	9
6	900.52396	450.76562	L	1091.61457	546.31092	8
7	987.55599	494.28163	S	978.53051	489.76889	7
8	1102.58294	551.79511	D	891.49848	446.25288	6
9	1203.63061	602.31895	T	776.47154	388.73941	5
10	1316.71468	658.86098	I	675.42386	338.21557	4
11	1373.73614	687.37171	G	562.33979	281.67353	3
12	1502.77874	751.89301	E	505.31833	253.16280	2
13			K-TMT6plex	376.27574	188.64151	1

FL0008614.raw #66517 RT: 126.4407 min
ITMS, 940.0278@cid35.00, z=+2, Mono m/z=939.52710 Da, MH+=1878.04692 Da, Match Tol.=0.6 Da

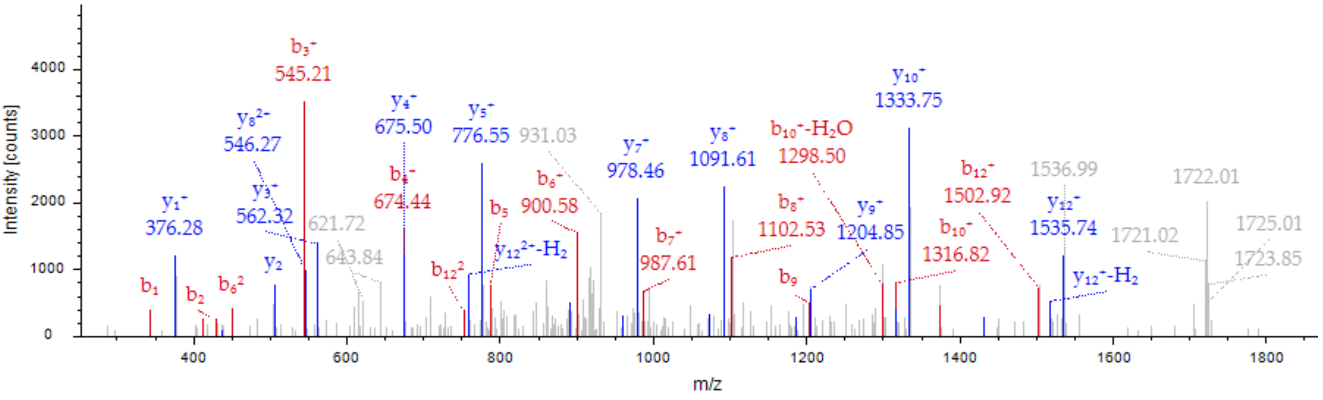

LHVQQHQQNQNR (Q96LC9)

Xcorr: 3.94

#1	b^+	b^{2+}	b^{3+}	b^{4+}	Seq.	y^+	y^{2+}	y^{3+}	y^{4+}	#2
1	343.25427	172.13077	115.08961	86.56903	L-TMT6plex					12
2	480.31318	240.66023	160.77591	120.83375	H	1416.68393	708.84560	472.89949	354.92644	11
3	579.38160	290.19444	193.79872	145.60086	V	1279.62502	640.31615	427.21319	320.66171	10
4	707.44018	354.22373	236.48491	177.61550	Q	1180.55661	590.78194	394.19039	295.89461	9
5	835.49875	418.25301	279.17110	209.63015	Q	1052.49803	526.75265	351.50419	263.87996	8
6	972.55766	486.78247	324.85741	243.89487	H	924.43945	462.72336	308.81800	231.86532	7
7	1100.61624	550.81176	367.54360	275.90952	Q	787.38054	394.19391	263.13170	197.60059	6
8	1228.67482	614.84105	410.22979	307.92416	Q	659.32196	330.16462	220.44551	165.58595	5
9	1342.71775	671.86251	448.24410	336.43489	N	531.26338	266.13533	177.75931	133.57130	4
10	1470.77632	735.89180	490.93029	368.44954	Q	417.22046	209.11387	139.74500	105.06057	3
11	1584.81925	792.91326	528.94460	396.96027	N	289.16188	145.08458	97.05881	73.04593	2
12					R	175.11895	88.06311	59.04450	44.53520	1

FL0008572.raw #11213 RT: 28.1796 min
ITMS, 440.4875@cid35.00, z=+4, Mono m/z=440.48749 Da, MH+=1758.92812 Da, Match Tol.=0.6 Da

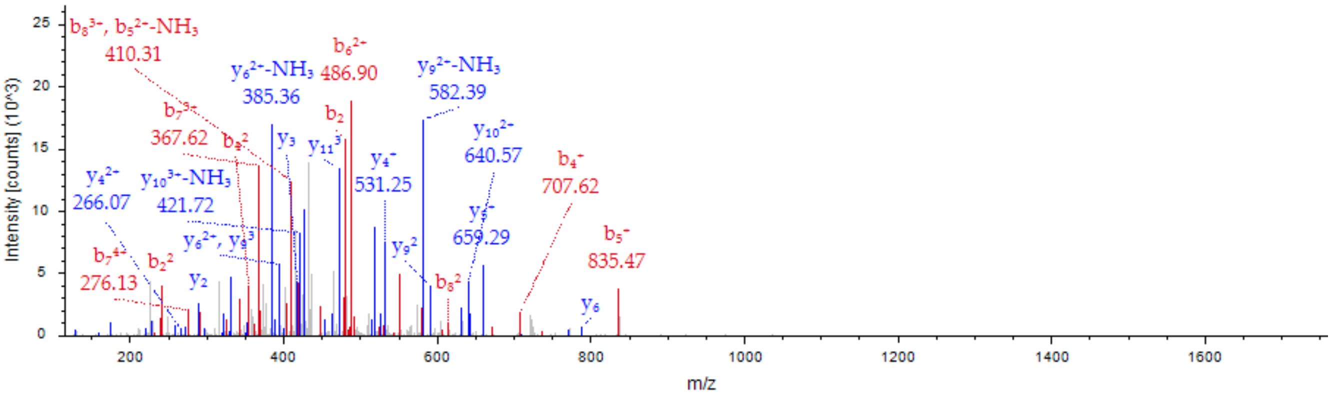

VPFLVLECPNLK (A0A024RDJ1)

Xcorr: 4.02

#1	b⁺	b²⁺	Seq.	y⁺	y²⁺	#2
1	329.23862	165.12295	V-TMT6plex			12
2	426.29139	213.64933	P	1558.88645	779.94686	11
3	573.35980	287.18354	F	1461.83369	731.42048	10
4	686.44386	343.72557	L	1314.76527	657.88628	9
5	785.51228	393.25978	V	1201.68121	601.34424	8
6	898.59634	449.80181	L	1102.61280	551.81004	7
7	1027.63893	514.32311	E	989.52873	495.26800	6
8	1187.66958	594.33843	C-Carbami...	860.48614	430.74671	5
9	1284.72235	642.86481	P	700.45549	350.73138	4
10	1398.76527	699.88628	N	603.40273	302.20500	3
11	1511.84934	756.42831	L	489.35980	245.18354	2
12			K-TMT6plex	376.27574	188.64151	1

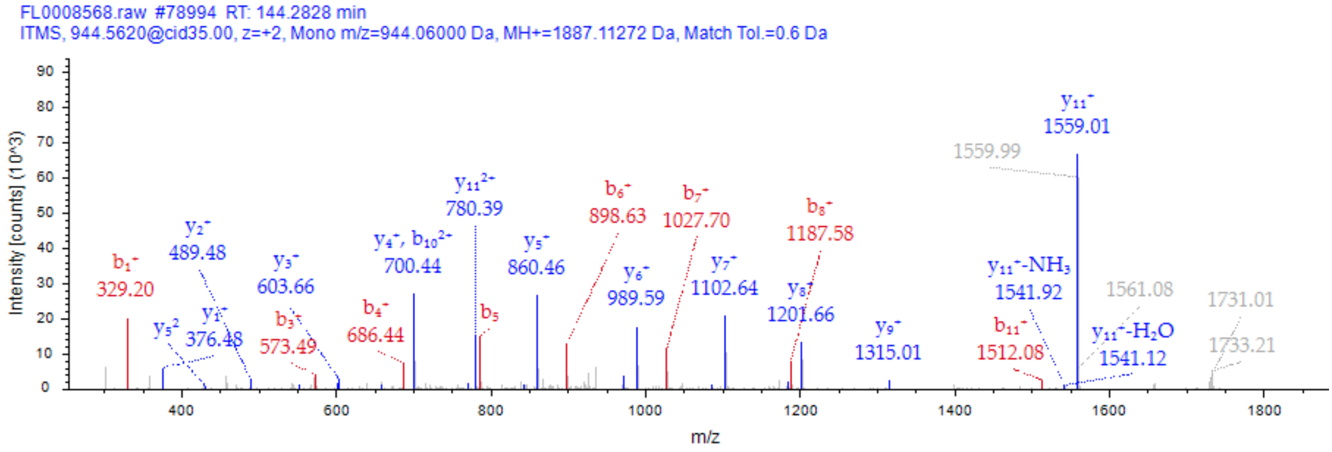

SHEAEAEPSEPR (Q9NQG1)

Xcorr: 3.79

#1	b+	b2+	b3+	Seq.	y+	y2+	y3+	#2
1	317.20224	159.10476	106.40560	S-TMT6plex				12
2	454.26115	227.63421	152.09190	H	1251.56002	626.28365	417.85819	11
3	583.30374	292.15551	195.10610	E	1114.50111	557.75419	372.17189	10
4	654.34086	327.67407	218.78514	A	985.45851	493.23290	329.15769	9
5	783.38345	392.19536	261.79933	E	914.42140	457.71434	305.47865	8
6	854.42056	427.71392	285.47837	A	785.37881	393.19304	262.46445	7
7	983.46316	492.23522	328.49257	E	714.34169	357.67449	238.78542	6
8	1080.51592	540.76160	360.84349	P	585.29910	293.15319	195.77122	5
9	1167.54795	584.27761	389.85417	S	488.24634	244.62681	163.42030	4
10	1296.59054	648.79891	432.86836	E	401.21431	201.11079	134.40962	3
11	1393.64330	697.32529	465.21929	P	272.17172	136.58950	91.39542	2
12				R	175.11895	88.06311	59.04450	1

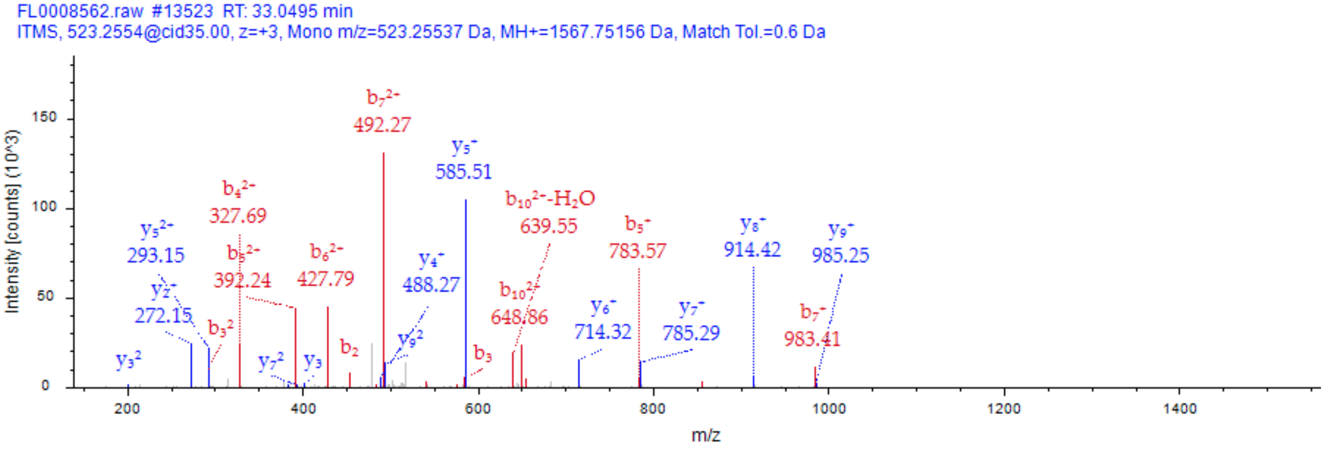

AEANQHVVDEK
(P49795)

Xcorr: 2.98

#1	b+	b2+	b3+	Seq.	y+	y2+	y3+	#2
1	301.20732	151.10730	101.07396	A-TMT6plex				11
2	430.24992	215.62860	144.08816	E	1397.72222	699.36475	466.57893	10
3	501.28703	251.14715	167.76719	A	1268.67963	634.84345	423.56473	9
4	615.32996	308.16862	205.78150	N	1197.64252	599.32490	399.88569	8
5	743.38853	372.19791	248.46770	Q	1083.59959	542.30343	361.87138	7
6	880.44745	440.72736	294.15400	H	955.54101	478.27414	319.18519	6
7	979.51586	490.26157	327.17680	V	818.48210	409.74469	273.49888	5
8	1078.58427	539.79578	360.19961	V	719.41369	360.21048	240.47608	4
9	1193.61122	597.30925	398.54192	D	620.34527	310.67627	207.45328	3
10	1322.65381	661.83054	441.55612	E	505.31833	253.16280	169.11096	2
11				K-TMT6plex	376.27574	188.64151	126.09676	1

FL0008560.raw #22579 RT: 49.9115 min
ITMS, 566.6445@cid35.00, z=+3, Mono m/z=566.64453 Da, MH+=1697.91904 Da, Match Tol.=0.6 Da

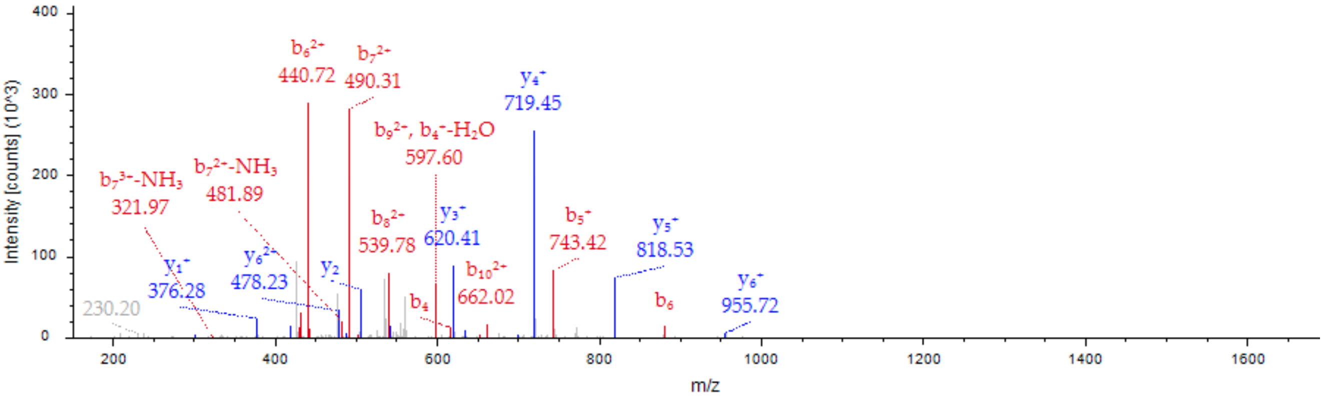

NDGALYHNNEEK (A0A024RDT6)

Xcorr: 3.91

#1	b+	b2+	Seq.	y+	y2+	#2
1	344.21314	172.61021	N-TMT6plex			12
2	459.24008	230.12368	D	1518.73860	759.87294	11
3	516.26154	258.63441	G	1403.71166	702.35947	10
4	587.29866	294.15297	A	1346.69019	673.84874	9
5	700.38272	350.69500	L	1275.65308	638.33018	8
6	863.44605	432.22666	Y	1162.56902	581.78815	7
7	1000.50496	500.75612	H	999.50569	500.25648	6
8	1114.54789	557.77758	N	862.44678	431.72703	5
9	1228.59082	614.79905	N	748.40385	374.70556	4
10	1357.63341	679.32034	E	634.36092	317.68410	3
11	1486.67600	743.84164	E	505.31833	253.16280	2
12			K-TMT6plex	376.27574	188.64151	1

FL0008562.raw #24762 RT: 53.5747 min
ITMS, 931.9763@cid35.00, z=+2, Mono m/z=931.47314 Da, MH+=1861.93901 Da, Match Tol.=0.6 Da

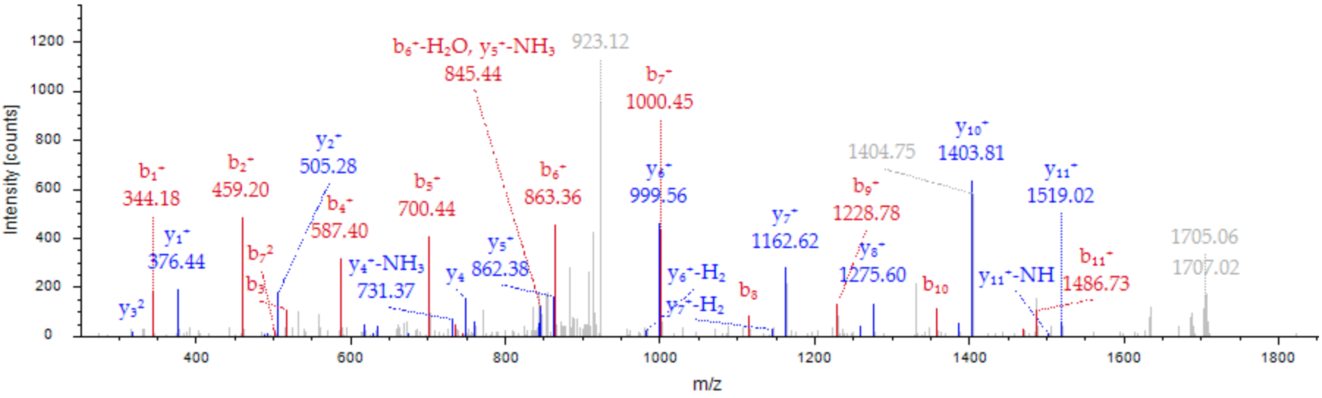

VHSDEDNPER (J3KTA2)

Xcorr: 3.65

#1	b⁺	b²⁺	b³⁺	Seq.	y⁺	y²⁺	y³⁺	#2
1	329.23862	165.12295	110.41773	V-TMT6plex				10
2	466.29753	233.65241	156.10403	H	1098.44466	549.72597	366.81974	9
3	553.32956	277.16842	185.11471	S	961.38574	481.19651	321.13343	8
4	668.35651	334.68189	223.45702	D	874.35372	437.68050	292.12276	7
5	797.39910	399.20319	266.47122	E	759.32677	380.16702	253.78044	6
6	912.42604	456.71666	304.81353	D	630.28418	315.64573	210.76624	5
7	1026.46897	513.73812	342.82784	N	515.25724	258.13226	172.42393	4
8	1123.52173	562.26450	375.17876	P	401.21431	201.11079	134.40962	3
9	1252.56433	626.78580	418.19296	E	304.16155	152.58441	102.05870	2
10				R	175.11895	88.06311	59.04450	1

FL0008566.raw #12127 RT: 30.0934 min
ITMS, 476.2291@cid35.00, z=+3, Mono m/z=476.22906 Da, MH+=1426.67264 Da, Match Tol.=0.6 Da

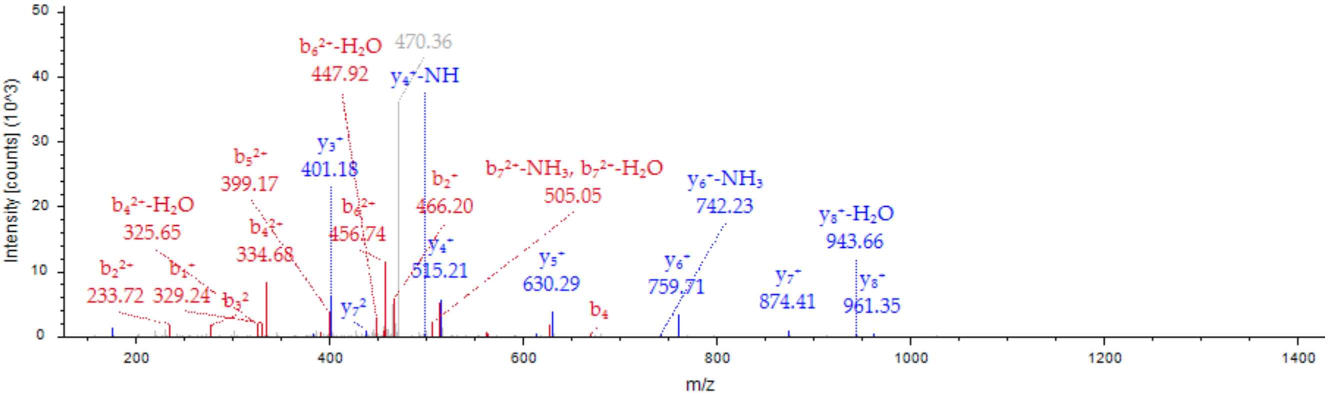

SLQPSVLMQLK (Q9GZQ3)

Xcorr: 3.77

#1	b⁺	b²⁺	Seq.	y⁺	y²⁺	#2
1	317.20224	159.10476	S-TMT6plex			11
2	430.28630	215.64679	L	1385.83877	693.42303	10
3	558.34488	279.67608	Q	1272.75471	636.88099	9
4	655.39764	328.20246	P	1144.69613	572.85170	8
5	742.42967	371.71847	S	1047.64337	524.32532	7
6	841.49808	421.25268	V	960.61134	480.80931	6
7	954.58215	477.79471	L	861.54293	431.27510	5
8	1085.62263	543.31495	M	748.45886	374.73307	4
9	1213.68121	607.34424	Q	617.41838	309.21283	3
10	1326.76527	663.88628	L	489.35980	245.18354	2
11			K-TMT6plex	376.27574	188.64151	1

FL0008620.raw #67074 RT: 124.1445 min
ITMS, 851.5203@cid35.00, z=+2, Mono m/z=851.52032 Da, MH+=1702.03337 Da, Match Tol.=0.6 Da

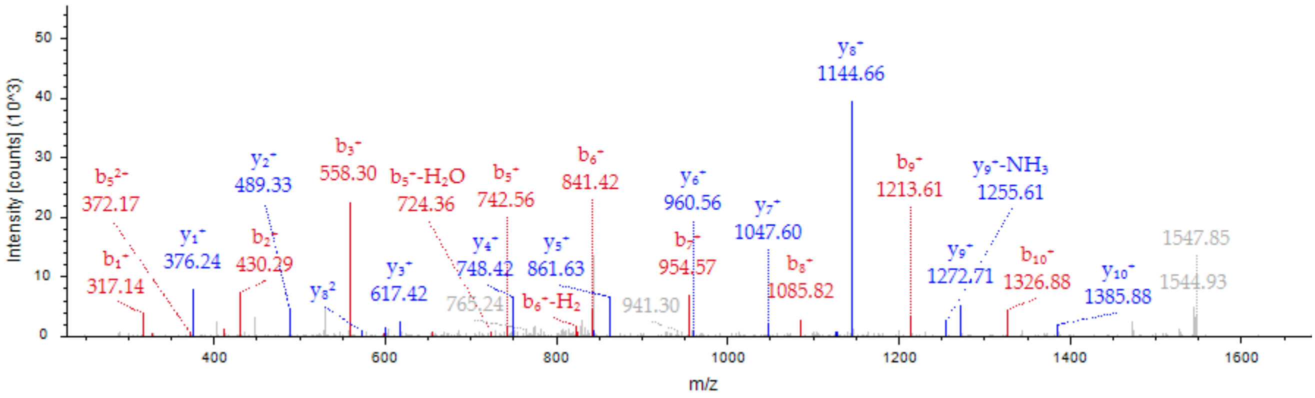

LAFAYLEDLHSEFDEQHGK (I1VE18)

Xcorr: 5.01

#1	b^{+}	b^{2+}	b^{3+}	b^{4+}	Seq.	y^{+}	y^{2+}	y^{3+}	y^{4+}	#2
1	343.25427	172.13077	115.08961	86.56903	L-TMT6plex					19
2	414.29139	207.64933	138.76865	104.32830	A	2365.12981	1183.06854	789.04812	592.03791	18
3	561.35980	281.18354	187.79145	141.09541	F	2294.09269	1147.54998	765.36908	574.27863	17
4	632.39691	316.70210	211.47049	158.85469	A	2147.02428	1074.01578	716.34628	537.51153	16
5	795.46024	398.23376	265.82493	199.62052	Y	2075.98716	1038.49722	692.66724	519.75225	15
6	908.54431	454.77579	303.51962	227.89153	L	1912.92384	956.96556	638.31280	478.98642	14
7	1037.58690	519.29709	346.53382	260.15218	E	1799.83977	900.42352	600.61811	450.71540	13
8	1152.61384	576.81056	384.87613	288.90892	D	1670.79718	835.90223	557.60391	418.45475	12
9	1265.69791	633.35259	422.57082	317.17993	L	1555.77024	778.38876	519.26160	389.69802	11
10	1402.75682	701.88205	468.25712	351.44466	H	1442.68617	721.84672	481.56691	361.42700	10
11	1489.78885	745.39806	497.26780	373.20267	S	1305.62726	653.31727	435.88060	327.16227	9
12	1618.83144	809.91936	540.28200	405.46332	E	1218.59523	609.80125	406.86993	305.40427	8
13	1765.89985	883.45356	589.30480	442.23042	F	1089.55264	545.27996	363.85573	273.14362	7
14	1880.92680	940.96704	627.64712	470.98716	D	942.48423	471.74575	314.83293	236.37651	6
15	2009.96939	1005.48833	670.66131	503.24780	E	827.45728	414.23228	276.49061	207.61978	5
16	2138.02797	1069.51762	713.34751	535.26245	Q	698.41469	349.71098	233.47641	175.35913	4
17	2275.08688	1138.04708	759.03381	569.52718	H	570.35611	285.68169	190.79022	143.34449	3
18	2332.10834	1166.55781	778.04097	583.78254	G	433.29720	217.15224	145.10392	109.07976	2
19					K-TMT6plex	376.27574	188.64151	126.09676	94.82439	1

FL0008628.raw #78338 RT: 142.8208 min
ITMS, 677.8498@cid35.00, z=+4, Mono m/z=677.59924 Da, MH+=2707.37514 Da, Match Tol.=0.6 Da

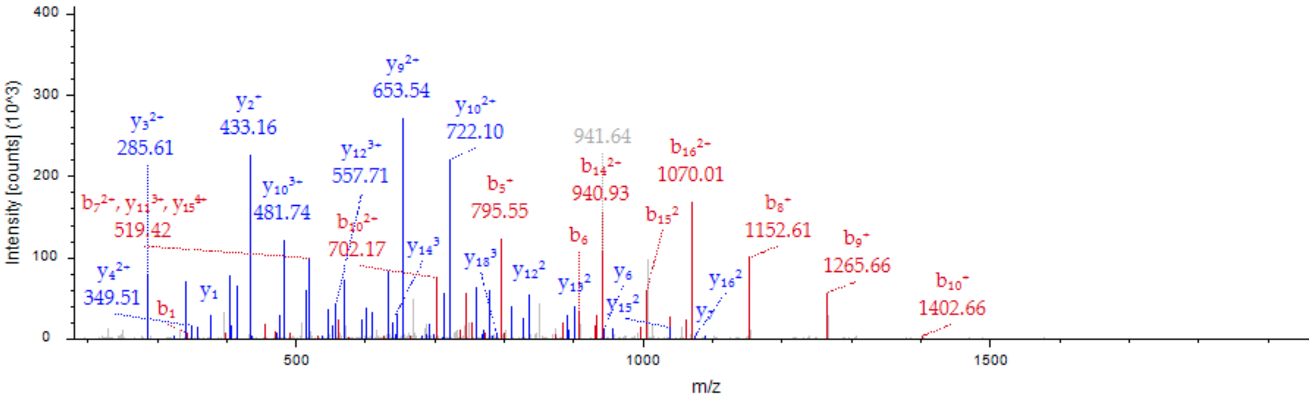

TLSSLYSEMQK (Q86WC4)

Xcorr: 3.11

#1	b+	b2+	Seq.	y+	y2+	#2
1	331.21789	166.11258	T-TMT6plex			11
2	444.30195	222.65461	L	1414.74493	707.87610	10
3	531.33398	266.17063	S	1301.66087	651.33407	9
4	618.36601	309.68664	S	1214.62884	607.81806	8
5	731.45007	366.22867	L	1127.59681	564.30204	7
6	894.51340	447.76034	Y	1014.51275	507.76001	6
7	981.54543	491.27635	S	851.44942	426.22835	5
8	1110.58802	555.79765	E	764.41739	382.71233	4
9	1241.62851	621.31789	M	635.37480	318.19104	3
10	1369.68708	685.34718	Q	504.33431	252.67080	2
11			K-TMT6plex	376.27574	188.64151	1

FL0008644.raw #61657 RT: 113.2214 min
ITMS, 872.9808@cid35.00, z=+2, Mono m/z=872.98077 Da, MH+=1744.95427 Da, Match Tol.=0.6 Da

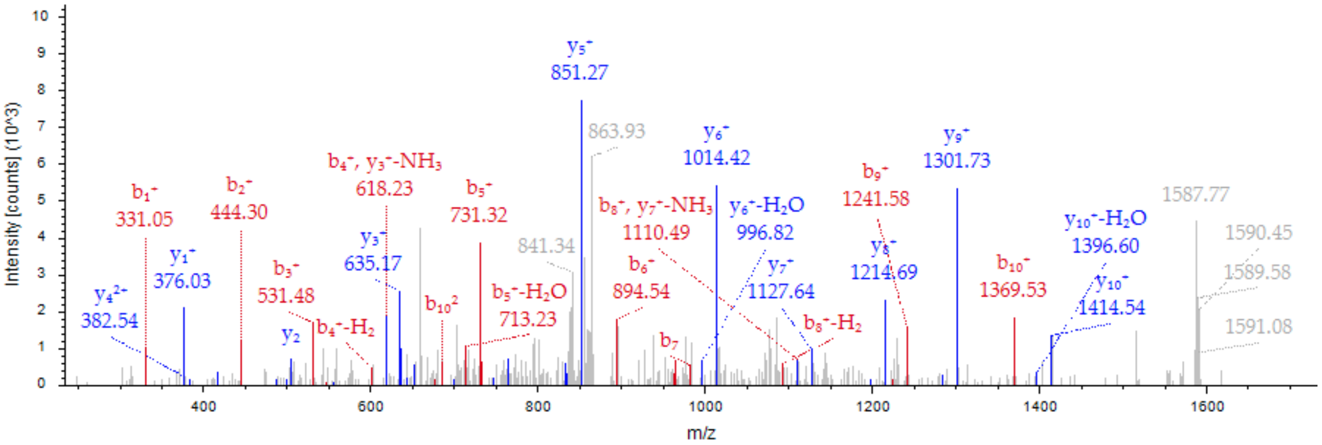

TGAVYYVAEIGAK (B4DRC8)

Xcorr: 3.62

#1	b⁺	b²⁺	Seq.	y⁺	y²⁺	#2
1	331.21789	166.11258	T-TMT6plex			12
2	388.23935	194.62331	G	1306.75682	653.88205	11
3	459.27646	230.14187	A	1249.73535	625.37132	10
4	558.34488	279.67608	V	1178.69824	589.85276	9
5	721.40821	361.20774	Y	1079.62983	540.31855	8
6	820.47662	410.74195	V	916.56650	458.78689	7
7	891.51373	446.26051	A	817.49808	409.25268	6
8	1020.55633	510.78180	E	746.46097	373.73412	5
9	1133.64039	567.32383	I	617.41838	309.21283	4
10	1190.66186	595.83457	G	504.33431	252.67080	3
11	1261.69897	631.35312	A	447.31285	224.16006	2
12			K-TMT6plex	376.27574	188.64151	1

FL0008628.raw #49969 RT: 94.8705 min
ITMS, 818.9887@cid35.00, z=+2, Mono m/z=818.98871 Da, MH+=1636.97014 Da, Match Tol.=0.6 Da

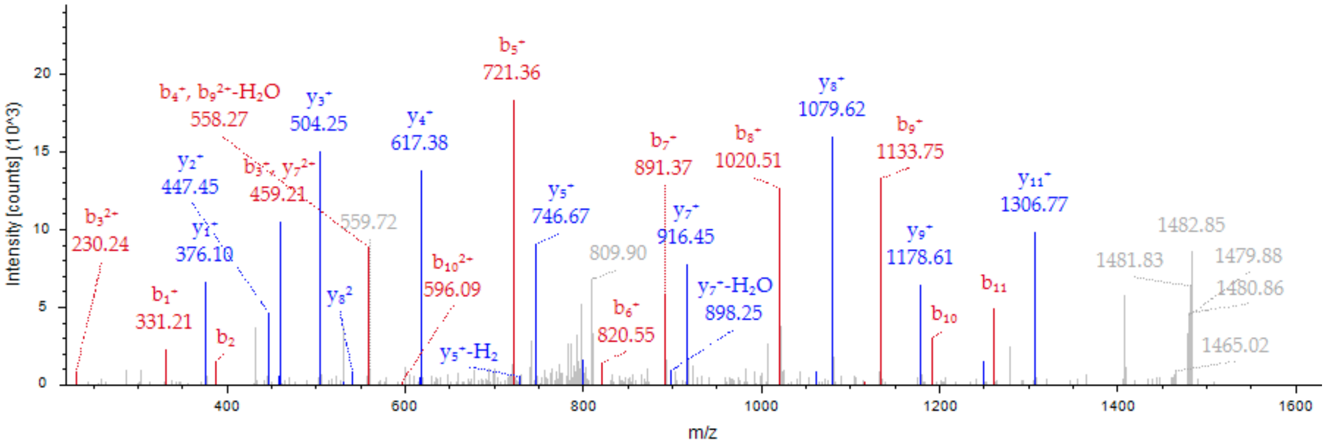

VTLPGQEEPWDIR (A0A024R8C7)

Xcorr: 3.96

#1	b+	b2+	Seq.	y+	y2+	#2
1	329.23862	165.12295	V-TMT6plex			13
2	430.28630	215.64679	T	1440.71177	720.85952	12
3	543.37036	272.18882	L	1339.66409	670.33568	11
4	640.42313	320.71520	P	1226.58003	613.79365	10
5	697.44459	349.22593	G	1129.52726	565.26727	9
6	825.50317	413.25522	Q	1072.50580	536.75654	8
7	954.54576	477.77652	E	944.44722	472.72725	7
8	1083.58836	542.29782	E	815.40463	408.20595	6
9	1180.64112	590.82420	P	686.36204	343.68466	5
10	1366.72043	683.86385	W	589.30927	295.15827	4
11	1481.74738	741.37733	D	403.22996	202.11862	3
12	1594.83144	797.91936	I	288.20302	144.60515	2
13			R	175.11895	88.06311	1

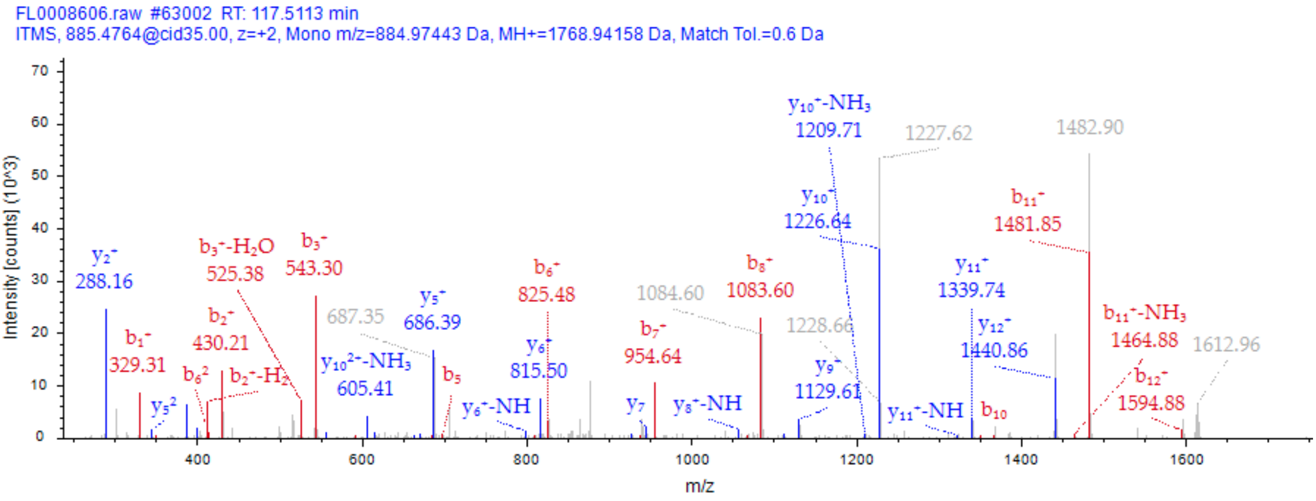

Xcorr: 3.48

FVNVVPTFGK (E9PR30)

#1	b+	b2+	Seq.	y+	y2+	#2
1	377.23862	189.12295	F-TMT6plex			10
2	476.30704	238.65716	V	1189.71423	595.36075	9
3	590.34996	295.67862	N	1090.64581	545.82654	8
4	689.41838	345.21283	V	976.60288	488.80508	7
5	788.48679	394.74703	V	877.53447	439.27087	6
6	885.53956	443.27342	P	778.46606	389.73667	5
7	986.58723	493.79726	T	681.41329	341.21028	4
8	1133.65565	567.33146	F	580.36561	290.68645	3
9	1190.67711	595.84219	G	433.29720	217.15224	2
10			K-TMT6plex	376.27574	188.64151	1

FL0008582.raw #68329 RT: 126.1514 min
ITMS, 783.4744@cid35.00, z=+2, Mono m/z=783.47443 Da, MH+=1565.94158 Da, Match Tol.=0.6 Da

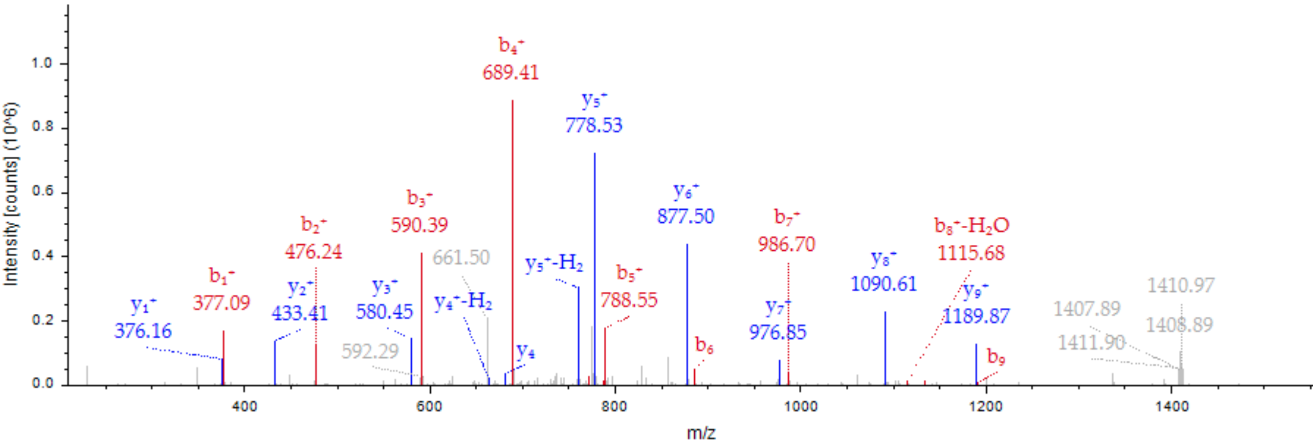

DDPQVMEDK (A6NDA2)

Xcorr: 3

#1	b+	b2+	Seq.	y+	y2+	#2
1	345.19715	173.10221	D-TMT6plex			9
2	460.22409	230.61569	D	1190.59245	595.79987	8
3	557.27686	279.14207	P	1075.56551	538.28639	7
4	685.33544	343.17136	Q	978.51275	489.76001	6
5	784.40385	392.70556	V	850.45417	425.73072	5
6	915.44433	458.22581	M	751.38576	376.19652	4
7	1044.48693	522.74710	E	620.34527	310.67627	3
8	1159.51387	580.26057	D	491.30268	246.15498	2
9			K-TMT6plex	376.27574	188.64151	1

FL0008606.raw #33826 RT: 69.0972 min
ITMS, 767.8938@cid35.00, z=+2, Mono m/z=767.89380 Da, MH+=1534.78032 Da, Match Tol.=0.6 Da

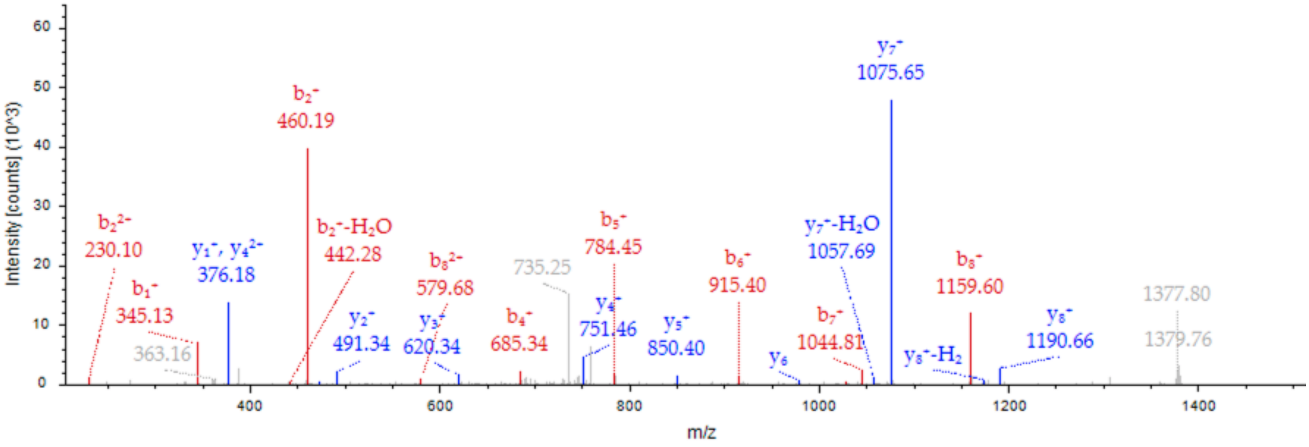

QNRPIPQWIR
(P62891)

Xcorr: 3.69

#1	b+	b2+	b3+	Seq.	y+	y2+	y3+	#2
1	358.22879	179.61803	120.08111	Q-TMT6plex				10
2	472.27171	236.63950	158.09542	N	1179.67454	590.34091	393.89636	9
3	628.37282	314.69005	210.12913	R	1065.63161	533.31944	355.88205	8
4	725.42559	363.21643	242.48005	P	909.53050	455.26889	303.84835	7
5	838.50965	419.75846	280.17474	I	812.47773	406.74251	271.49743	6
6	935.56242	468.28485	312.52566	P	699.39367	350.20047	233.80274	5
7	1063.62099	532.31414	355.21185	Q	602.34091	301.67409	201.45182	4
8	1249.70031	625.35379	417.23829	W	474.28233	237.64480	158.76563	3
9	1362.78437	681.89582	454.93297	I	288.20302	144.60515	96.73919	2
10				R	175.11895	88.06311	59.04450	1

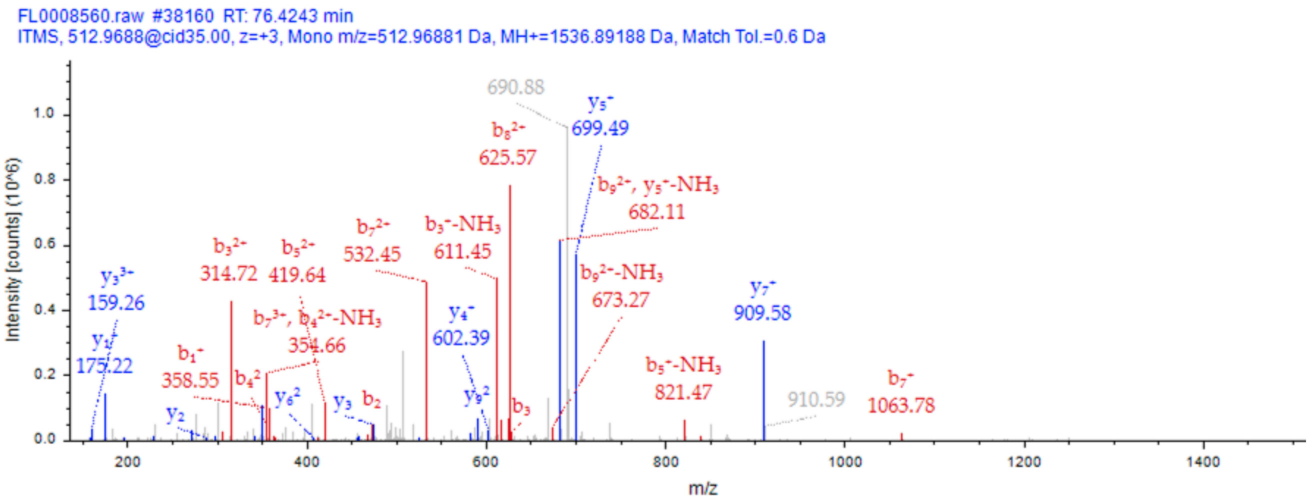

GTPFETPDQGK (Q53FV1)

Xcorr: 3.5

#1	b^+	b^{2+}	Seq.	y^+	y^{2+}	#2
1	287.19167	144.09947	G-TMT6plex			11
2	388.23935	194.62331	T	1348.69461	674.85094	10
3	485.29211	243.14970	P	1247.64693	624.32710	9
4	632.36053	316.68390	F	1150.59417	575.80072	8
5	761.40312	381.20520	E	1003.52576	502.26652	7
6	862.45080	431.72904	T	874.48316	437.74522	6
7	959.50356	480.25542	P	773.43548	387.22138	5
8	1074.53051	537.76889	D	676.38272	338.69500	4
9	1202.58908	601.79818	Q	561.35578	281.18153	3
10	1259.61055	630.30891	G	433.29720	217.15224	2
11			K-TMT6plex	376.27574	188.64151	1

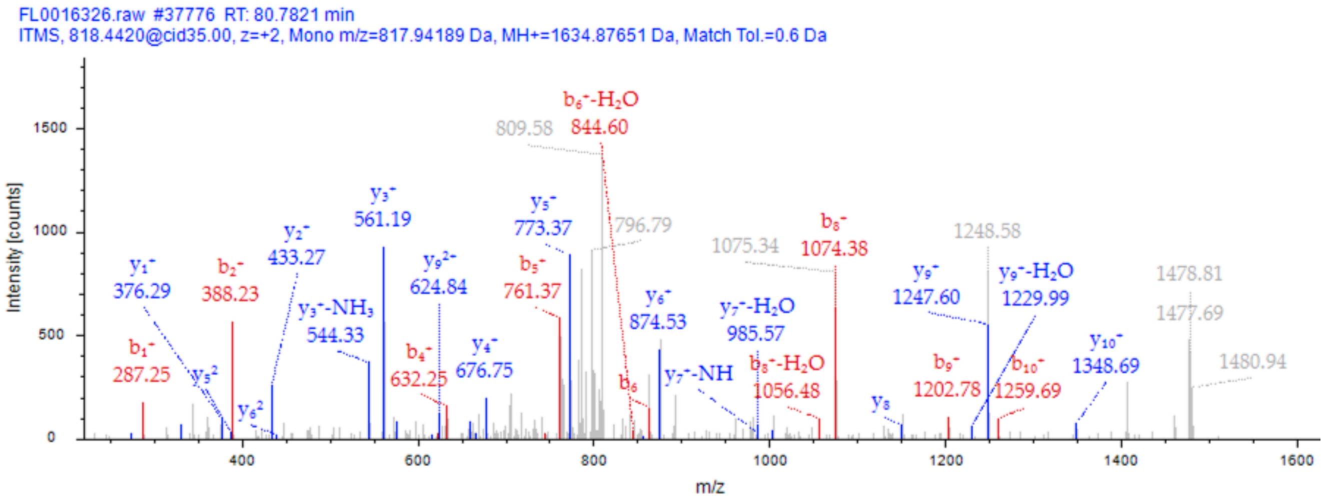

SVEESAGLVGLK
(Q96HJ9)

Xcorr: 3.02

#1	b⁺	b²⁺	Seq.	y⁺	y²⁺	#2
1	317.20224	159.10476	S-TMT6plex			12
2	416.27065	208.63896	V	1330.77795	665.89261	11
3	545.31324	273.16026	E	1231.70953	616.35841	10
4	674.35584	337.68156	E	1102.66694	551.83711	9
5	761.38787	381.19757	S	973.62435	487.31581	8
6	832.42498	416.71613	A	886.59232	443.79980	7
7	889.44644	445.22686	G	815.55521	408.28124	6
8	1002.53051	501.76889	L	758.53374	379.77051	5
9	1101.59892	551.30310	V	645.44968	323.22848	4
10	1158.62038	579.81383	G	546.38126	273.69427	3
11	1271.70445	636.35586	L	489.35980	245.18354	2
12			K-TMT6plex	376.27574	188.64151	1

FL0008568.raw #53696 RT: 101.3668 min
ITMS, 823.9886@cid35.00, z=+2, Mono m/z=823.98859 Da, MH+=1646.96990 Da, Match Tol.=0.6 Da

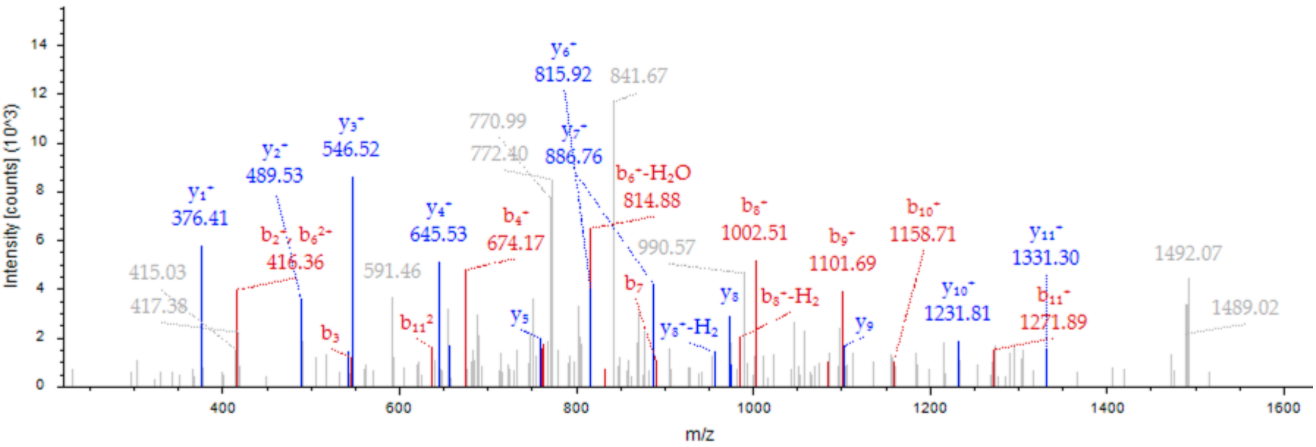

VLHDEILNKPMETLK (Q9Y2D2)

Xcorr: 3.96

#1	b⁺	b²⁺	b³⁺	b⁴⁺	Seq.	y⁺	y²⁺	y³⁺	y⁴⁺	#2
1	329.23862	165.12295	110.41773	83.06511	V-TMT6plex					15
2	442.32269	221.66498	148.11241	111.33613	L	2139.22478	1070.11603	713.74645	535.56165	14
3	579.38160	290.19444	193.79872	145.60086	H	2026.14072	1013.57400	676.05176	507.29064	13
4	694.40854	347.70791	232.14103	174.35759	D	1889.08181	945.04454	630.36545	473.02591	12
5	823.45113	412.22921	275.15523	206.61824	E	1774.05486	887.53107	592.02314	444.26917	11
6	936.53520	468.77124	312.84992	234.88926	I	1645.01227	823.00977	549.00894	412.00853	10
7	1049.61926	525.31327	350.54461	263.16027	L	1531.92821	766.46774	511.31425	383.73751	9
8	1163.66219	582.33473	388.55891	291.67100	N	1418.84414	709.92571	473.61957	355.46649	8
9	1520.92008	760.96368	507.64488	380.98548	K-TMT6plex	1304.80121	652.90425	435.60526	326.95576	7
10	1617.97285	809.49006	539.99580	405.24867	P	947.54332	474.27530	316.51929	237.64129	6
11	1749.01333	875.01030	583.67596	438.00879	M	850.49056	425.74892	284.16837	213.37810	5
12	1878.05593	939.53160	626.69016	470.26944	E	719.45007	360.22867	240.48821	180.61798	4
13	1979.10360	990.05544	660.37272	495.53136	T	590.40748	295.70738	197.47401	148.35733	3
14	2092.18767	1046.59747	698.06741	523.80237	L	489.35980	245.18354	163.79145	123.09541	2
15					K-TMT6plex	376.27574	188.64151	126.09676	94.82439	1

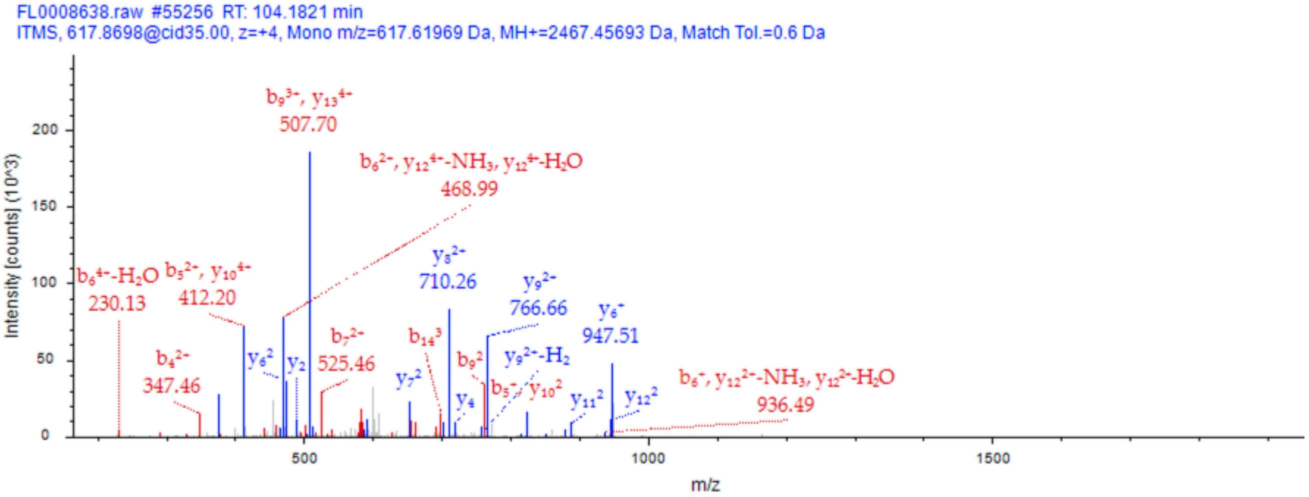

Xcorr: 3.35

EAPEGWQTPK (Q9NWT8)

#1	b+	b2+	Seq.	y+	y2+	#2
1	359.21280	180.11004	E-TMT6plex			10
2	430.24992	215.62860	A	1242.66800	621.83764	9
3	527.30268	264.15498	P	1171.63089	586.31908	8
4	656.34527	328.67627	E	1074.57813	537.79270	7
5	713.36674	357.18701	G	945.53553	473.27140	6
6	899.44605	450.22666	W	888.51407	444.76067	5
7	1027.50463	514.25595	Q	702.43476	351.72102	4
8	1128.55230	564.77979	T	574.37618	287.69173	3
9	1225.60507	613.30617	P	473.32850	237.16789	2
10			K-TMT6plex	376.27574	188.64151	1

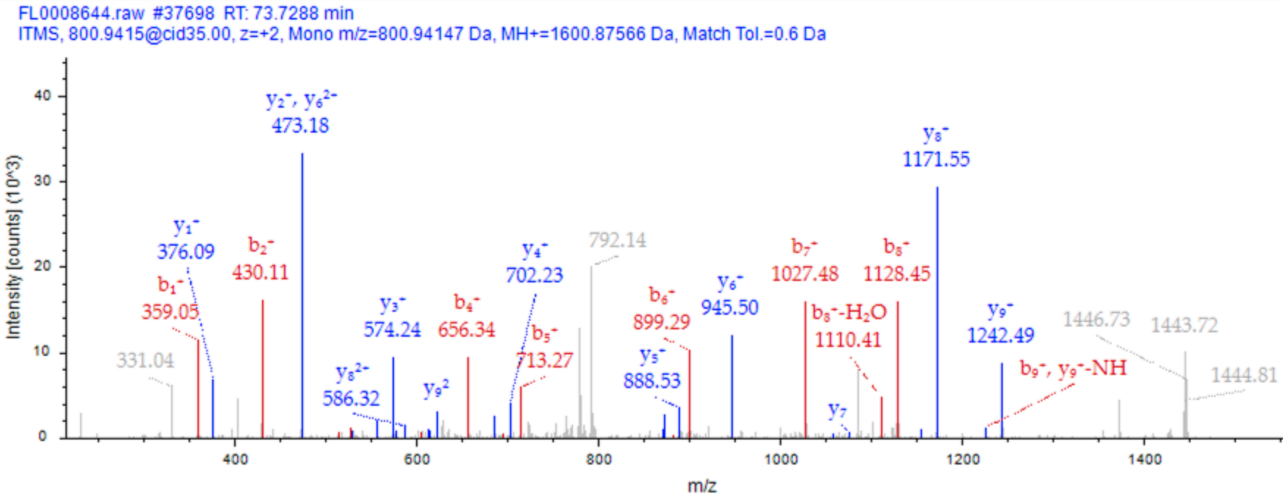

HAAGAANAGPAAGPR (Q8N5H3)

Xcorr: 3.99

#1	b⁺	b²⁺	b³⁺	Seq.	y⁺	y²⁺	y³⁺	#2
1	367.22912	184.11820	123.08122	H-TMT6plex				15
2	438.26623	219.63676	146.76026	A	1151.59159	576.29944	384.53538	14
3	509.30335	255.15531	170.43930	A	1080.55448	540.78088	360.85634	13
4	566.32481	283.66604	189.44645	G	1009.51737	505.26232	337.17731	12
5	637.36193	319.18460	213.12549	A	952.49590	476.75159	318.17015	11
6	708.39904	354.70316	236.80453	A	881.45879	441.23303	294.49111	10
7	822.44197	411.72462	274.81884	N	810.42168	405.71448	270.81208	9
8	893.47908	447.24318	298.49788	A	696.37875	348.69301	232.79777	8
9	950.50054	475.75391	317.50503	G	625.34163	313.17446	209.11873	7
10	1047.55331	524.28029	349.85595	P	568.32017	284.66372	190.11157	6
11	1118.59042	559.79885	373.53499	A	471.26741	236.13734	157.76065	5
12	1189.62754	595.31741	397.21403	A	400.23029	200.61879	134.08162	4
13	1246.64900	623.82814	416.22118	G	329.19318	165.10023	110.40258	3
14	1343.70176	672.35452	448.57211	P	272.17172	136.58950	91.39542	2
15				R	175.11895	88.06311	59.04450	1

FL0008620.raw #10627 RT: 26.9810 min
ITMS, 506.6094@cid35.00, z=+3, Mono m/z=506.60941 Da, MH+=1517.81366 Da, Match Tol.=0.6 Da

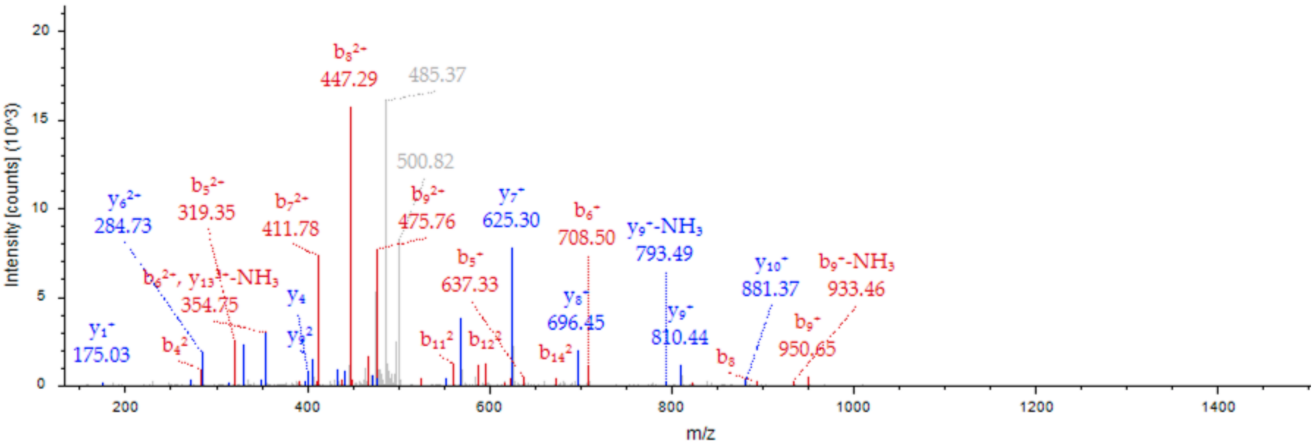

LLFNPSGPYQK (Q9UBR5)

Xcorr: 2.72

#1	b+	b2+	Seq.	y+	y2+	#2
1	343.25427	172.13077	L-TMT6plex			11
2	456.33834	228.67281	L	1379.75207	690.37967	10
3	603.40675	302.20701	F	1266.66800	633.83764	9
4	717.44968	359.22848	N	1119.59959	560.30343	8
5	814.50244	407.75486	P	1005.55666	503.28197	7
6	901.53447	451.27087	S	908.50390	454.75559	6
7	958.55593	479.78161	G	821.47187	411.23957	5
8	1055.60870	528.30799	P	764.45041	382.72884	4
9	1218.67203	609.83965	Y	667.39764	334.20246	3
10	1346.73060	673.86894	Q	504.33431	252.67080	2
11			K-TMT6plex	376.27574	188.64151	1

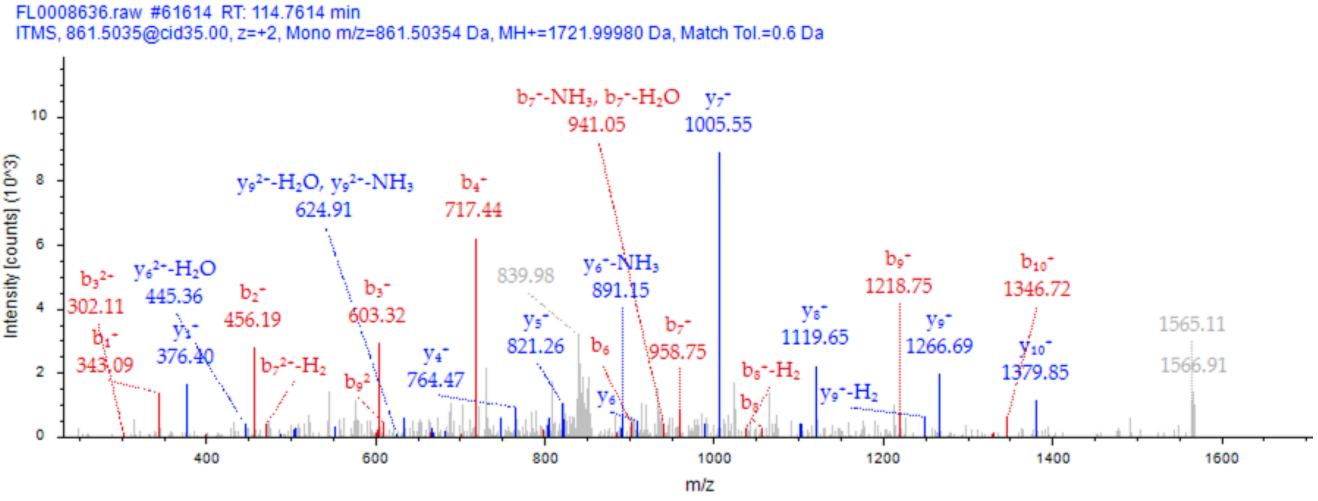

AGSIDGTDEDPHDR
(Q16560)

Xcorr: 4.33

#1	b^{+}	b^{2+}	b^{3+}	Seq.	y^{+}	y^{2+}	y^{3+}	#2
1	301.20732	151.10730	101.07396	A-TMT6plex				14
2	358.22879	179.61803	120.08111	G	1413.58769	707.29748	471.86741	13
3	445.26081	223.13405	149.09179	S	1356.56623	678.78675	452.86026	12
4	558.34488	279.67608	186.78648	I	1269.53420	635.27074	423.84958	11
5	673.37182	337.18955	225.12879	D	1156.45013	578.72871	386.15490	10
6	730.39328	365.70028	244.13595	G	1041.42319	521.21523	347.81258	9
7	831.44096	416.22412	277.81851	T	984.40173	492.70450	328.80543	8
8	946.46791	473.73759	316.16082	D	883.35405	442.18066	295.12287	7
9	1075.51050	538.25889	359.17502	E	768.32711	384.66719	256.78055	6
10	1190.53744	595.77236	397.51733	D	639.28451	320.14590	213.76636	5
11	1287.59021	644.29874	429.86825	P	524.25757	262.63242	175.42404	4
12	1424.64912	712.82820	475.55456	H	427.20481	214.10604	143.07312	3
13	1539.67606	770.34167	513.89687	D	290.14590	145.57659	97.38682	2
14				R	175.11895	88.06311	59.04450	1

FL0008622.raw #17806 RT: 41.9720 min
ITMS, 571.9330@cid35.00, z=+3, Mono m/z=571.93298 Da, MH+=1713.78440 Da, Match Tol.=0.6 Da

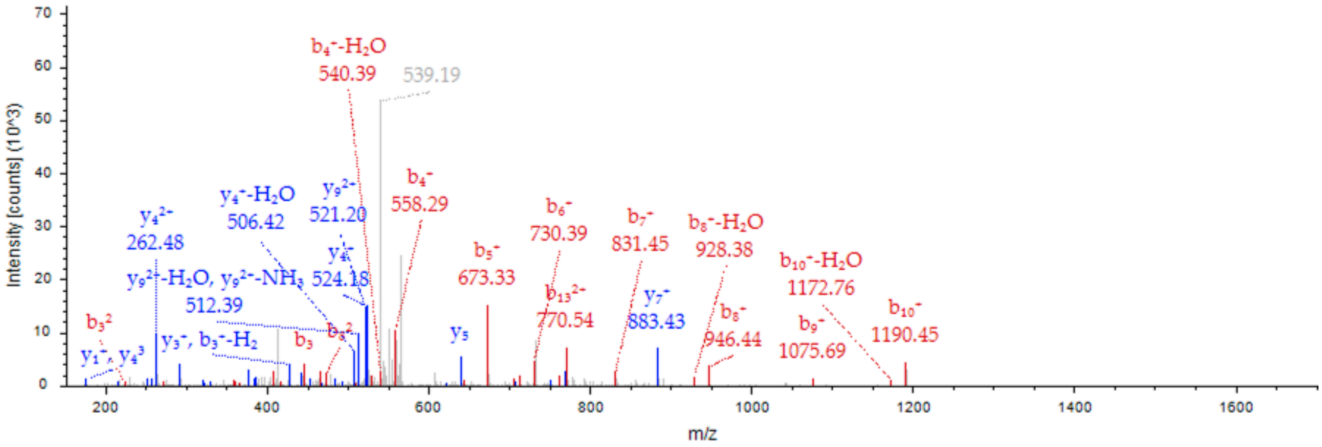

Xcorr: 2.92

QAMQEAR (Q9NRQ5)

#1	b⁺	b²⁺	Seq.	y⁺	y²⁺	#2
1	358.22879	179.61803	Q-TMT6plex			7
2	429.26590	215.13659	A	705.33483	353.17106	6
3	560.30638	280.65683	M	634.29772	317.65250	5
4	688.36496	344.68612	Q	503.25724	252.13226	4
5	817.40755	409.20742	E	375.19866	188.10297	3
6	888.44467	444.72597	A	246.15607	123.58167	2
7			R	175.11895	88.06311	1

FL0008644.raw #10624 RT: 26.4887 min
ITMS, 531.7817@cid35.00, z=+2, Mono m/z=531.78168 Da, MH+=1062.55608 Da, Match Tol.=0.6 Da

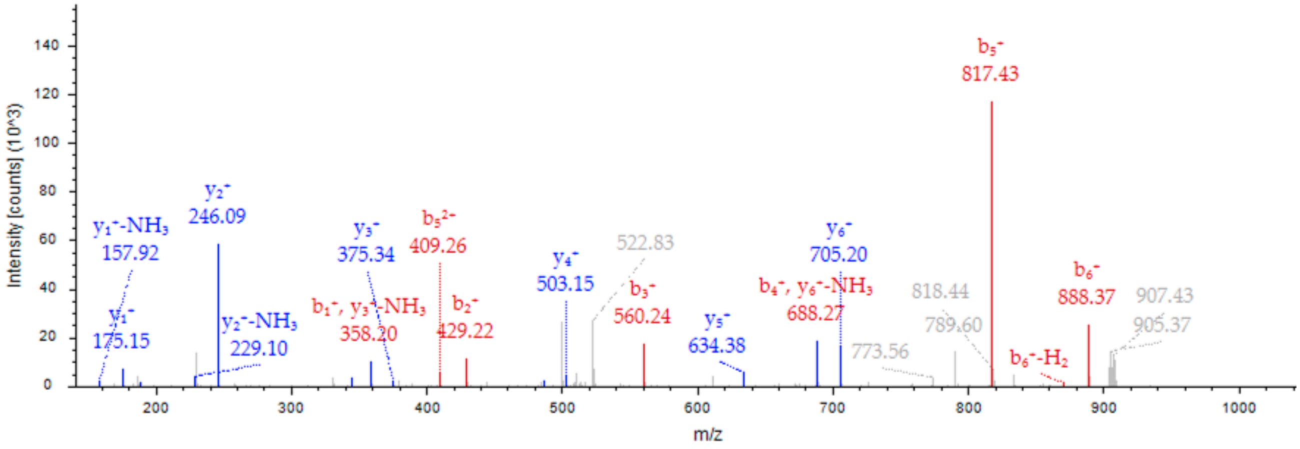

IIQFIQEQLK (Q502W6)

Xcorr: 3.69

#1	b⁺	b²⁺	Seq.	y⁺	y²⁺	#2
1	343.25427	172.13077	I-TMT6plex			10
2	456.33834	228.67281	I	1375.81467	688.41097	9
3	584.39691	292.70210	Q	1262.73060	631.86894	8
4	731.46533	366.23630	F	1134.67203	567.83965	7
5	844.54939	422.77833	I	987.60361	494.30544	6
6	972.60797	486.80762	Q	874.51955	437.76341	5
7	1101.65056	551.32892	E	746.46097	373.73412	4
8	1229.70914	615.35821	Q	617.41838	309.21283	3
9	1342.79320	671.90024	L	489.35980	245.18354	2
10			K-TMT6plex	376.27574	188.64151	1

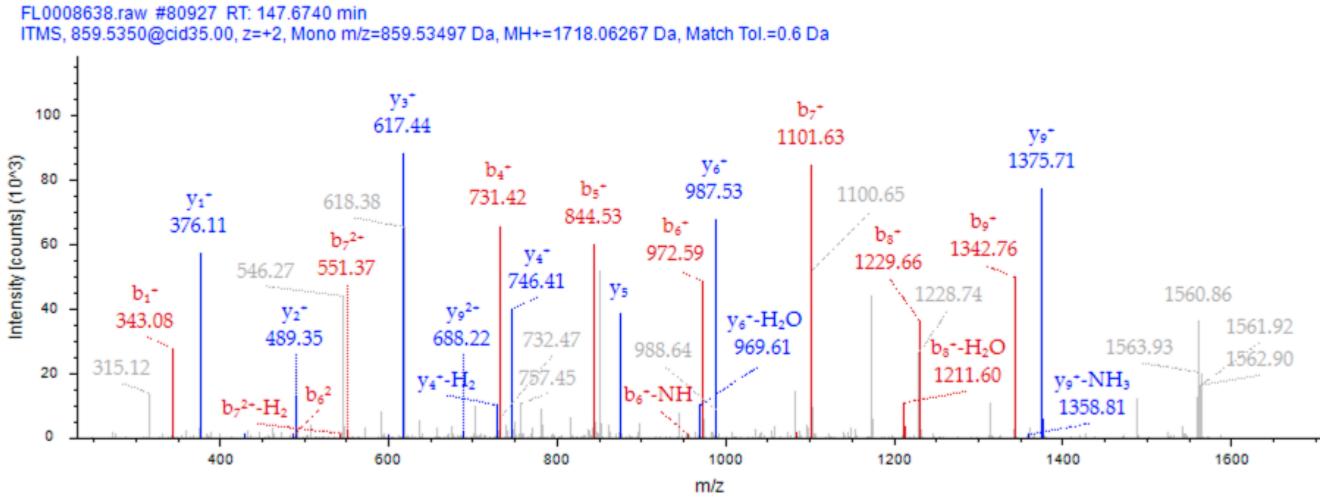

HWQEITFNLPVK
(A2A2G4)

Xcorr: 3.65

#1	b⁺	b²⁺	b³⁺	Seq.	y⁺	y²⁺	y³⁺	#2
1	367.22912	184.11820	123.08122	H-TMT6plex				12
2	553.30843	277.15785	185.10766	W	1603.90454	802.45591	535.30637	11
3	681.36701	341.18714	227.79385	Q	1417.82523	709.41625	473.27993	10
4	810.40960	405.70844	270.80805	E	1289.76665	645.38697	430.59374	9
5	923.49367	462.25047	308.50274	I	1160.72406	580.86567	387.57954	8
6	1024.54135	512.77431	342.18530	T	1047.64000	524.32364	349.88485	7
7	1171.60976	586.30852	391.20810	F	946.59232	473.79980	316.20229	6
8	1285.65269	643.32998	429.22241	N	799.52391	400.26559	267.17949	5
9	1398.73675	699.87201	466.91710	L	685.48098	343.24413	229.16518	4
10	1495.78952	748.39840	499.26802	P	572.39691	286.70210	191.47049	3
11	1594.85793	797.93260	532.29083	V	475.34415	238.17571	159.11957	2
12				K-TMT6plex	376.27574	188.64151	126.09676	1

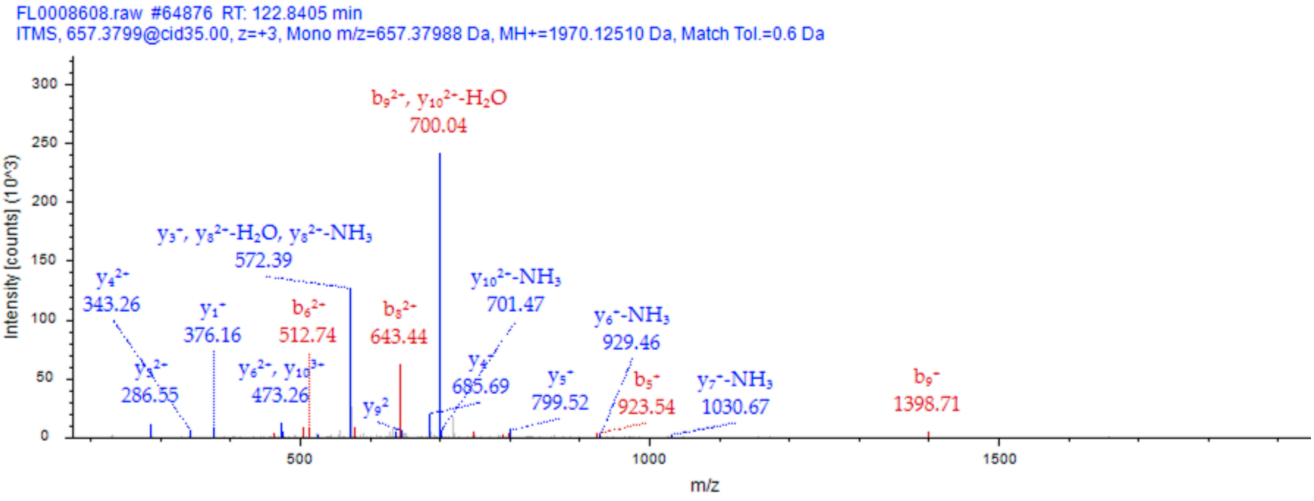

AVSAIVQK
(Q9UNW9)

Xcorr: 2.64

#1	b⁺	b²⁺	Seq.	y⁺	y²⁺	#2
1	301.20732	151.10730	A-TMT6plex			8
2	400.27574	200.64151	V	973.62435	487.31581	7
3	487.30776	244.15752	S	874.55593	437.78161	6
4	558.34488	279.67608	A	787.52391	394.26559	5
5	671.42894	336.21811	I	716.48679	358.74703	4
6	770.49736	385.75232	V	603.40273	302.20500	3
7	898.55593	449.78161	Q	504.33431	252.67080	2
8			K-TMT6plex	376.27574	188.64151	1

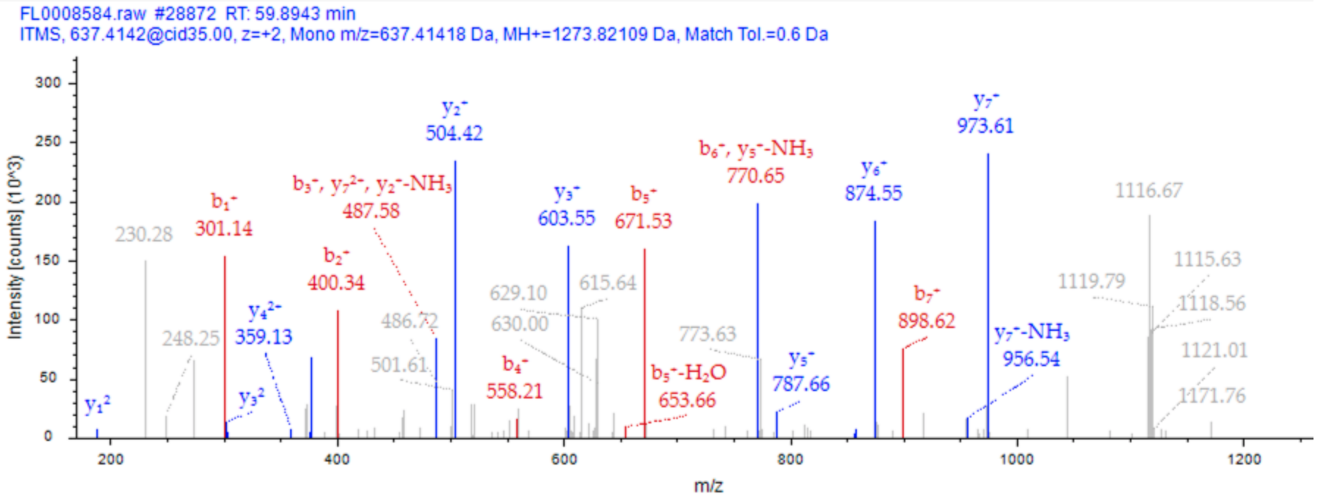

Xcorr: 2.48

KSPEIISGR (A0A087WVZ0)

#1	b⁺	b²⁺	b³⁺	Seq.	y⁺	y²⁺	y³⁺	#2
1	587.42810	294.21769	196.48089	K-TMT6ple...				9
2	674.46013	337.73370	225.49156	S	858.46796	429.73762	286.82750	8
3	771.51290	386.26009	257.84248	P	771.43593	386.22160	257.81683	7
4	900.55549	450.78138	300.85668	E	674.38317	337.69522	225.46591	6
5	1013.63955	507.32341	338.55137	I	545.34057	273.17392	182.45171	5
6	1126.72362	563.86545	376.24606	I	432.25651	216.63189	144.75702	4
7	1213.75564	607.38146	405.25673	S	319.17244	160.08986	107.06233	3
8	1270.77711	635.89219	424.26389	G	232.14042	116.57385	78.05166	2
9				R	175.11895	88.06311	59.04450	1

FL0008588.raw #27255 RT: 57.3575 min
ITMS, 482.2995@cid35.00, z=+3, Mono m/z=482.29950 Da, MH+=1444.88395 Da, Match Tol.=0.6 Da

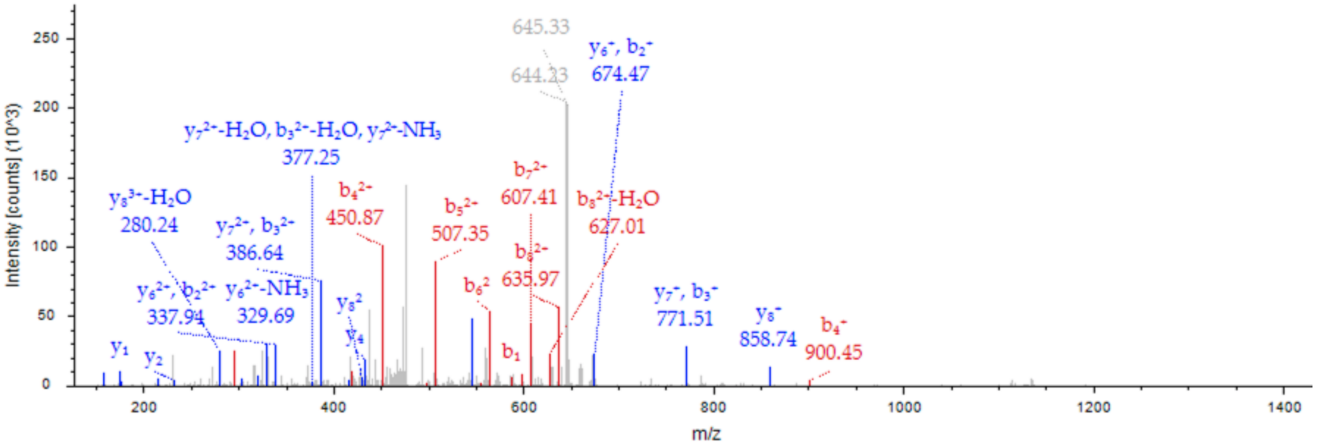

DRDLLVVAHDLIR (Q9NWS6)

Xcorr: 3.55

#1	b⁺	b²⁺	b³⁺	b⁴⁺	Seq.	y⁺	y²⁺	y³⁺	y⁴⁺	#2
1	345.19715	173.10221	115.73723	87.05475	D-TMT6plex					13
2	501.29826	251.15277	167.77094	126.08002	R	1419.84306	710.42517	473.95254	355.71622	12
3	616.32521	308.66624	206.11325	154.83676	D	1263.74195	632.37461	421.91883	316.69094	11
4	729.40927	365.20827	243.80794	183.10777	L	1148.71500	574.86114	383.57652	287.93421	10
5	842.49333	421.75030	281.50263	211.37879	L	1035.63094	518.31911	345.88183	259.66319	9
6	941.56175	471.28451	314.52543	236.14589	V	922.54688	461.77708	308.18714	231.39218	8
7	1040.63016	520.81872	347.54824	260.91300	V	823.47846	412.24287	275.16434	206.62507	7
8	1111.66727	556.33728	371.22728	278.67228	A	724.41005	362.70866	242.14153	181.85797	6
9	1248.72619	624.86673	416.91358	312.93700	H	653.37293	327.19011	218.46250	164.09869	5
10	1363.75313	682.38020	455.25589	341.69374	D	516.31402	258.66065	172.77619	129.83396	4
11	1476.83719	738.92224	492.95058	369.96476	L	401.28708	201.14718	134.43388	101.07723	3
12	1589.92126	795.46427	530.64527	398.23577	I	288.20302	144.60515	96.73919	72.80621	2
13					R	175.11895	88.06311	59.04450	44.53520	1

FL0008626.raw #61829 RT: 114.3799 min
ITMS, 441.7636@cid35.00, z=+4, Mono m/z=441.76361 Da, MH+=1764.03261 Da, Match Tol.=0.6 Da

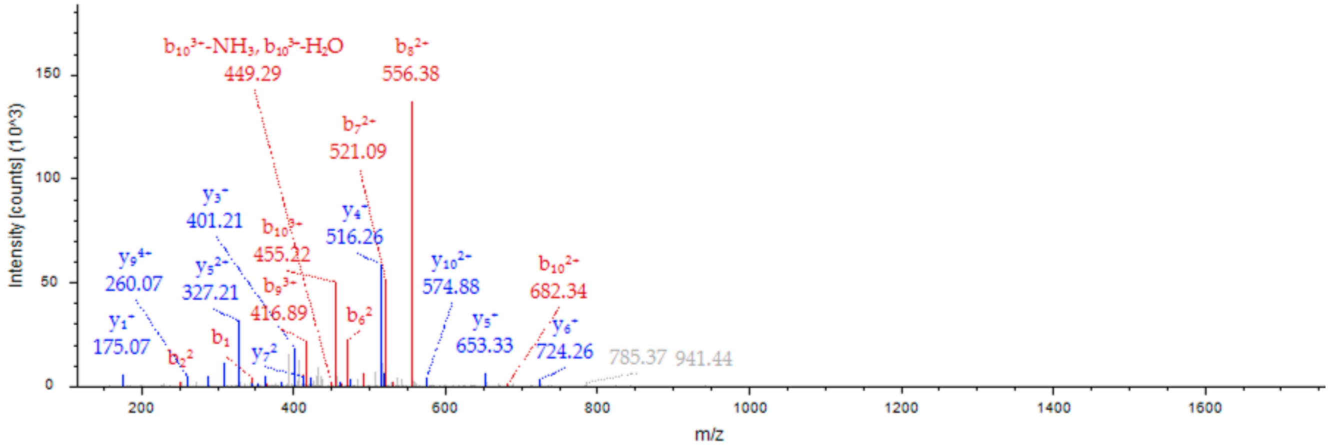

NPVLVSQYEK (A0A024R8K0)

Xcorr: 3.24

#1	b⁺	b²⁺	Seq.	y⁺	y²⁺	#2
1	344.21314	172.61021	N-TMT6plex			10
2	441.26590	221.13659	P	1291.74592	646.37660	9
3	540.33431	270.67080	V	1194.69316	597.85022	8
4	653.41838	327.21283	L	1095.62474	548.31601	7
5	752.48679	376.74703	V	982.54068	491.77398	6
6	839.51882	420.26305	S	883.47226	442.23977	5
7	967.57740	484.29234	Q	796.44024	398.72376	4
8	1130.64073	565.82400	Y	668.38166	334.69447	3
9	1259.68332	630.34530	E	505.31833	253.16280	2
10			K-TMT6plex	376.27574	188.64151	1

FL0008582.raw #45173 RT: 87.4051 min
ITMS, 817.9769@cid35.00, z=+2, Mono m/z=817.97693 Da, MH+=1634.94658 Da, Match Tol.=0.6 Da

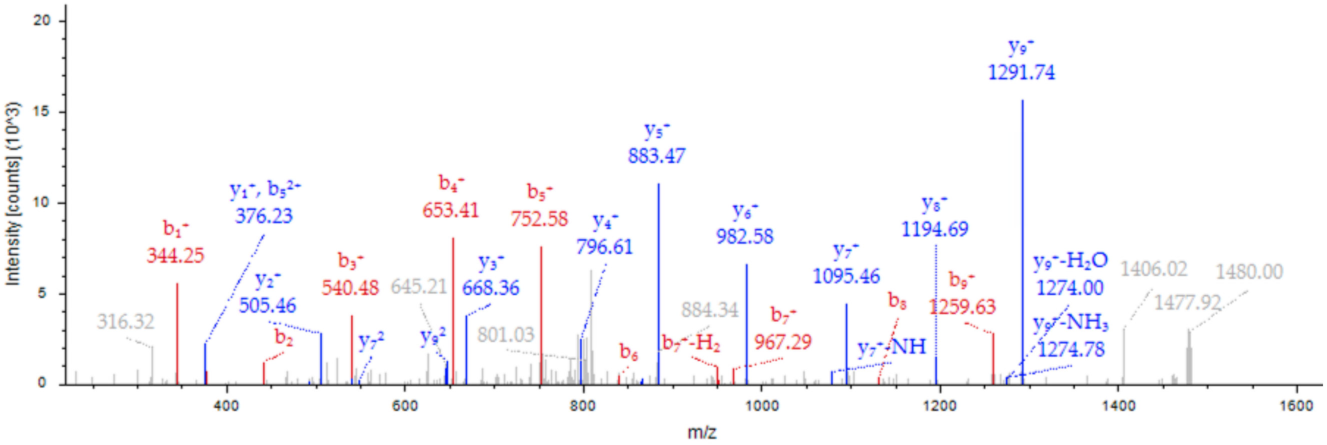

VQTLEAWVIHGGR
(B2R880)

Xcorr: 4.1

#1	b⁺	b²⁺	b³⁺	Seq.	y⁺	y²⁺	y³⁺	#2
1	329.23862	165.12295	110.41773	V-TMT6plex				13
2	457.29720	229.15224	153.10392	Q	1366.72261	683.86494	456.24572	12
3	558.34488	279.67608	186.78648	T	1238.66403	619.83565	413.55953	11
4	671.42894	336.21811	224.48117	L	1137.61635	569.31181	379.87697	10
5	800.47154	400.73941	267.49536	E	1024.53229	512.76978	342.18228	9
6	871.50865	436.25796	291.17440	A	895.48970	448.24849	299.16808	8
7	1057.58796	529.29762	353.20084	W	824.45258	412.72993	275.48905	7
8	1156.65638	578.83183	386.22364	V	638.37327	319.69027	213.46261	6
9	1269.74044	635.37386	423.91833	I	539.30486	270.15607	180.43980	5
10	1406.79935	703.90331	469.60463	H	426.22079	213.61403	142.74511	4
11	1463.82082	732.41405	488.61179	G	289.16188	145.08458	97.05881	3
12	1520.84228	760.92478	507.61894	G	232.14042	116.57385	78.05166	2
13				R	175.11895	88.06311	59.04450	1

FL0008606.raw #71850 RT: 132.4565 min
ITMS, 565.9902@cid35.00, z=+3, Mono m/z=565.65582 Da, MH+=1694.95292 Da, Match Tol.=0.6 Da

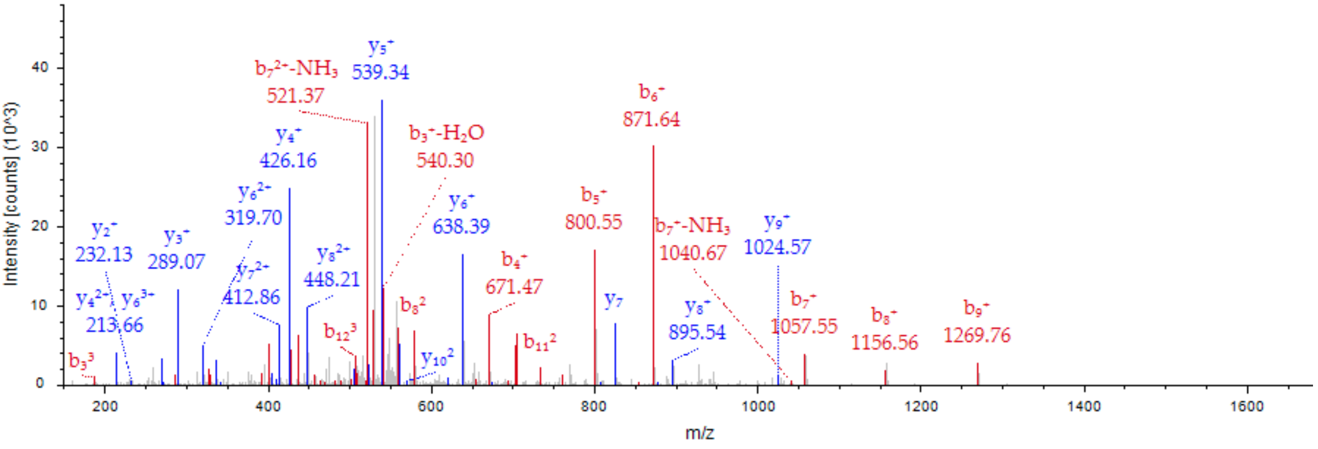

LENTEDIEEVEQHIQTIK (Q9NUN5)

Xcorr: 4.5

#1	b+	b2+	b3+	Seq.	y+	y2+	y3+	#2
1	343.25427	172.13077	115.08961	L-TMT6plex				18
2	472.29687	236.65207	158.10381	E	2284.15060	1142.57894	762.05505	17
3	586.33979	293.67353	196.11812	N	2155.10801	1078.05764	719.04085	16
4	687.38747	344.19737	229.80067	T	2041.06508	1021.03618	681.02654	15
5	816.43006	408.71867	272.81487	E	1940.01740	970.51234	647.34399	14
6	931.45701	466.23214	311.15719	D	1810.97481	905.99104	604.32979	13
7	1044.54107	522.77417	348.85187	I	1695.94787	848.47757	565.98747	12
8	1173.58366	587.29547	391.86607	E	1582.86380	791.93554	528.29279	11
9	1302.62626	651.81677	434.88027	E	1453.82121	727.41424	485.27859	10
10	1401.69467	701.35097	467.90307	V	1324.77862	662.89295	442.26439	9
11	1530.73726	765.87227	510.91727	E	1225.71020	613.35874	409.24159	8
12	1658.79584	829.90156	553.60346	Q	1096.66761	548.83744	366.22739	7
13	1795.85475	898.43102	599.28977	H	968.60903	484.80815	323.54120	6
14	1908.93882	954.97305	636.98446	I	831.55012	416.27870	277.85489	5
15	2036.99739	1019.00234	679.67065	Q	718.46606	359.73667	240.16020	4
16	2138.04507	1069.52617	713.35321	T	590.40748	295.70738	197.47401	3
17	2251.12914	1126.06821	751.04790	I	489.35980	245.18354	163.79145	2
18				K-TMT6plex	376.27574	188.64151	126.09676	1

FL0016322.raw #70247 RT: 135.9842 min
ITMS, 876.4711@cid35.00, z=+3, Mono m/z=876.13690 Da, MH+=2626.39615 Da, Match Tol.=0.6 Da

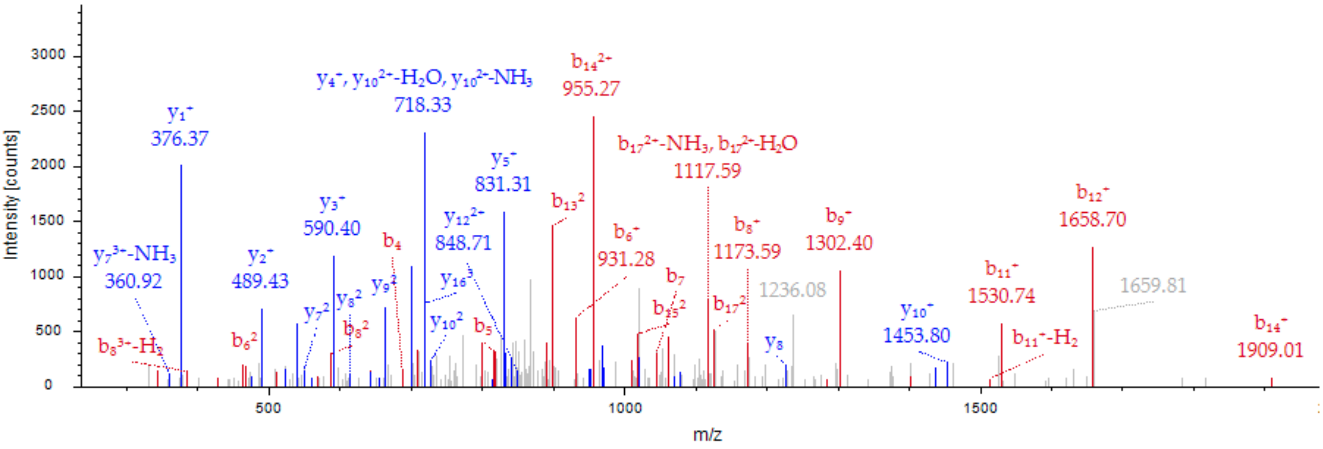

DASTLQSQK (A0A024R046)

Xcorr: 3.09

#1	b+	b2+	Seq.	y+	y2+	#2
1	345.19715	173.10221	D-TMT6plex			9
2	416.23427	208.62077	A	1091.62580	546.31654	8
3	503.26629	252.13679	S	1020.58869	510.79798	7
4	604.31397	302.66062	T	933.55666	467.28197	6
5	717.39804	359.20266	L	832.50898	416.75813	5
6	845.45661	423.23195	Q	719.42492	360.21610	4
7	932.48864	466.74796	S	591.36634	296.18681	3
8	1060.54722	530.77725	Q	504.33431	252.67080	2
9			K-TMT6plex	376.27574	188.64151	1

FL0016322.raw #26083 RT: 58.8410 min
ITMS, 718.4117@cid35.00, z=+2, Mono m/z=718.41168 Da, MH+=1435.81609 Da, Match Tol.=0.6 Da

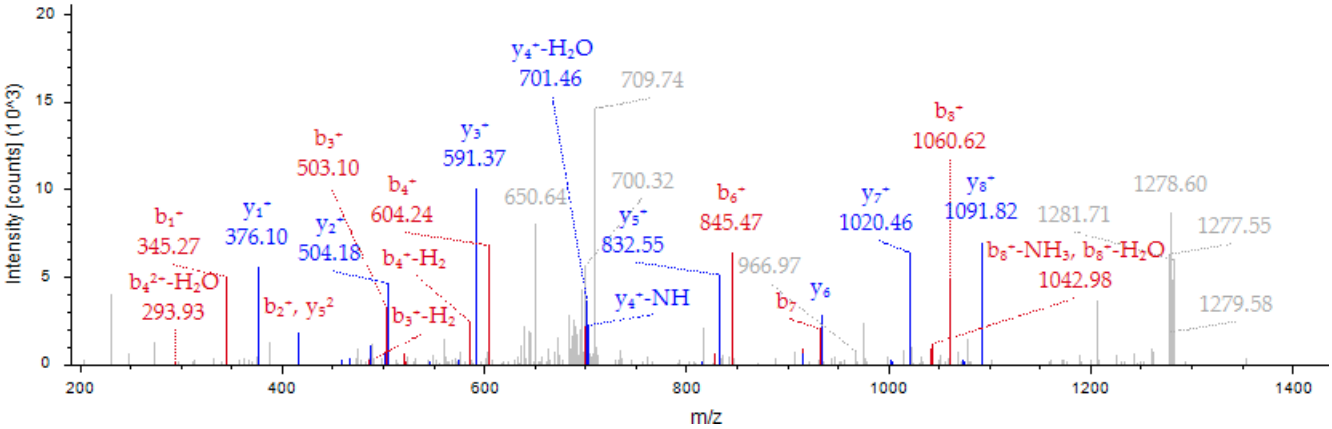

NSPVGVNHR
(Q99595)

Xcorr: 3.5

#1	b^{+}	b^{2+}	b^{3+}	Seq.	y^{+}	y^{2+}	y^{3+}	#2
1	344.21314	172.61021	115.40923	N-TMT6plex				9
2	431.24516	216.12622	144.41991	S	865.46388	433.23558	289.15948	8
3	528.29793	264.65260	176.77083	P	778.43185	389.71956	260.14880	7
4	627.36634	314.18681	209.79363	V	681.37908	341.19318	227.79788	6
5	684.38781	342.69754	228.80079	G	582.31067	291.65897	194.77507	5
6	783.45622	392.23175	261.82359	V	525.28921	263.14824	175.76792	4
7	897.49915	449.25321	299.83790	N	426.22079	213.61403	142.74511	3
8	1034.55806	517.78267	345.52420	H	312.17786	156.59257	104.73081	2
9				R	175.11895	88.06311	59.04450	1

FL0008638.raw #3643 RT: 11.8891 min
ITMS, 403.5615@cid35.00, z=+3, Mono m/z=403.56152 Da, MH+=1208.67002 Da, Match Tol.=0.6 Da

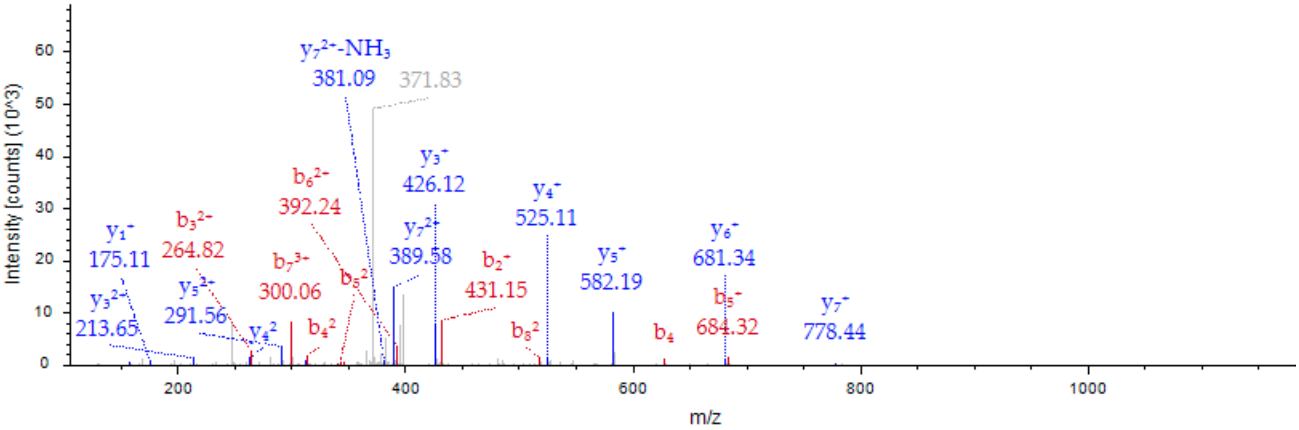

EQLSSSDTAPR
(B2RCL8)

Xcorr: 3.4

#1	b+	b2+	Seq.	y+	y2+	#2
1	359.21280	180.11004	E-TMT6plex			11
2	487.27138	244.13933	Q	1061.52218	531.26473	10
3	600.35544	300.68136	L	933.46360	467.23544	9
4	687.38747	344.19737	S	820.37954	410.69341	8
5	774.41950	387.71339	S	733.34751	367.17739	7
6	861.45153	431.22940	S	646.31548	323.66138	6
7	976.47847	488.74287	D	559.28345	280.14536	5
8	1077.52615	539.26671	T	444.25651	222.63189	4
9	1148.56326	574.78527	A	343.20883	172.10805	3
10	1245.61603	623.31165	P	272.17172	136.58950	2
11			R	175.11895	88.06311	1

FL0008642.raw #19515 RT: 42.7883 min
ITMS, 710.3668@cid35.00, z=+2, Mono m/z=710.36682 Da, MH+=1419.72637 Da, Match Tol.=0.6 Da

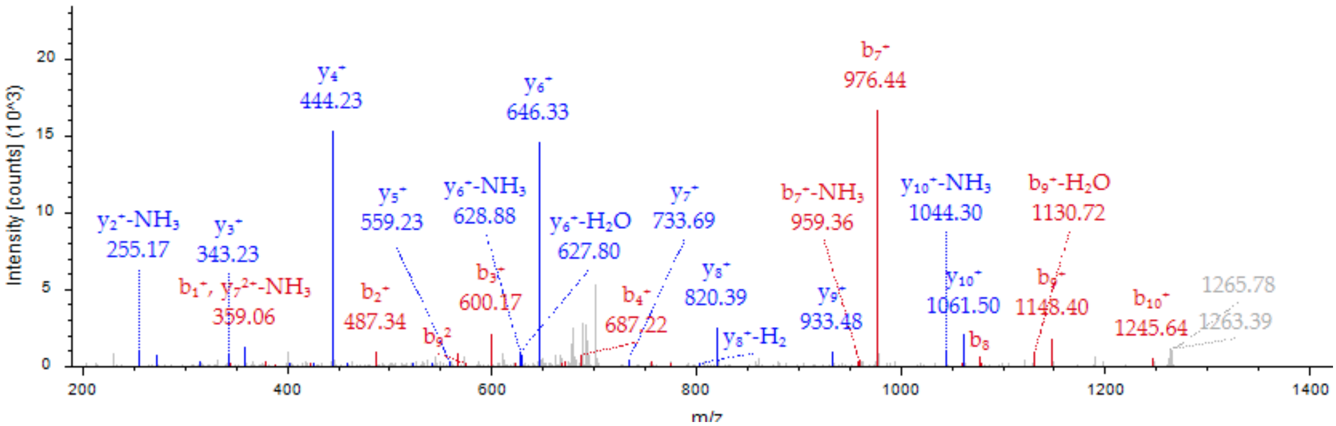

GHCQDAPDSVR
(C9JG63)

Xcorr: 3.34

#1	b⁺	b²⁺	b³⁺	Seq.	y⁺	y²⁺	y³⁺	#2
1	287.19167	144.09947	96.40208	G-TMT6plex				11
2	424.25058	212.62893	142.08838	H	1184.51130	592.75929	395.50862	10
3	584.28123	292.64425	195.43193	C-Carbami...	1047.45238	524.22983	349.82231	9
4	712.33981	356.67354	238.11812	Q	887.42174	444.21451	296.47876	8
5	827.36675	414.18701	276.46044	D	759.36316	380.18522	253.79257	7
6	898.40387	449.70557	300.13947	A	644.33621	322.67175	215.45026	6
7	995.45663	498.23195	332.49039	P	573.29910	287.15319	191.77122	5
8	1110.48357	555.74543	370.83271	D	476.24634	238.62681	159.42030	4
9	1197.51560	599.26144	399.84339	S	361.21939	181.11334	121.07798	3
10	1296.58402	648.79565	432.86619	V	274.18737	137.59732	92.06731	2
11				R	175.11895	88.06311	59.04450	1

FL0008636.raw #10675 RT: 28.0226 min
ITMS, 490.9033@cid35.00, z=+3, Mono m/z=490.90329 Da, MH+=1470.69532 Da, Match Tol.=0.6 Da

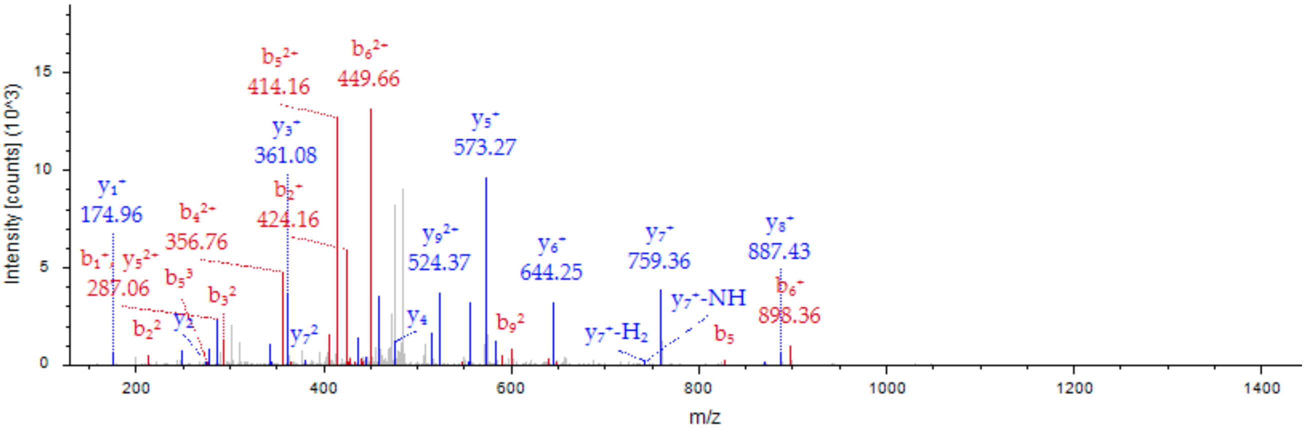

CTHNEGLNQK
(Q6ICH7)

Xcorr: 3.7

#1	b+	b2+	b3+	Seq.	y+	y2+	y3+	#2
1	390.20086	195.60407	130.73847	C-Carbami...				10
2	491.24854	246.12791	164.42103	T	1269.67488	635.34108	423.89648	9
3	628.30745	314.65736	210.10733	H	1168.62720	584.81724	390.21392	8
4	742.35037	371.67883	248.12164	N	1031.56829	516.28778	344.52761	7
5	871.39297	436.20012	291.13584	E	917.52536	459.26632	306.51330	6
6	928.41443	464.71085	310.14299	G	788.48277	394.74502	263.49911	5
7	1041.49850	521.25289	347.83768	L	731.46131	366.23429	244.49195	4
8	1155.54142	578.27435	385.85199	N	618.37724	309.69226	206.79726	3
9	1283.60000	642.30364	428.53818	Q	504.33431	252.67080	168.78296	2
10				K-TMT6plex	376.27574	188.64151	126.09676	1

FL0016322.raw #19246 RT: 46.3706 min
ITMS, 553.6274@cid35.00, z=+3, Mono m/z=553.62738 Da, MH+=1658.86759 Da, Match Tol.=0.6 Da

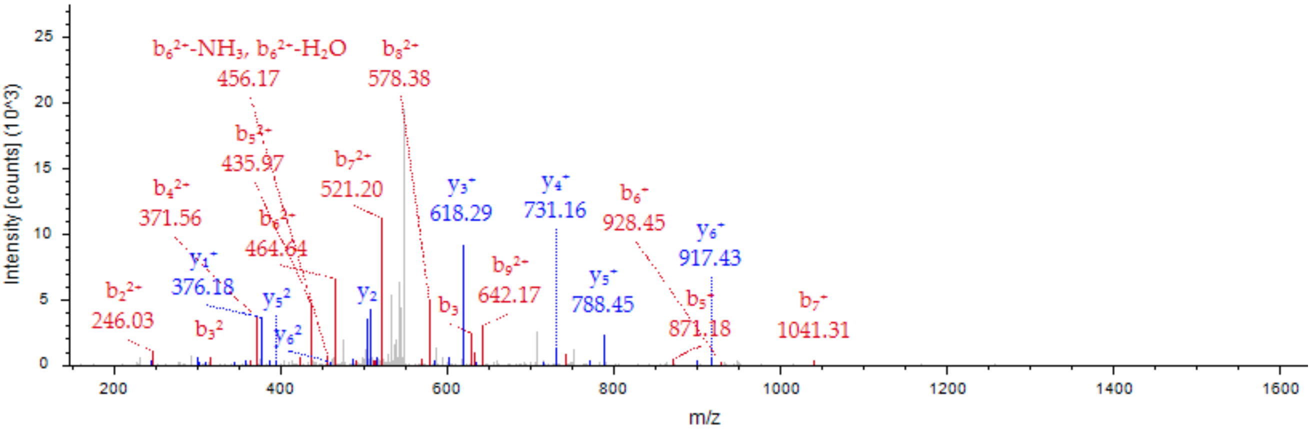

VGDNVLHLAQR (B7Z6L7)

Xcorr: 2.9

#1	b⁺	b²⁺	b³⁺	Seq.	y⁺	y²⁺	y³⁺	#2
1	329.23862	165.12295	110.41773	V-TMT6plex				11
2	386.26009	193.63368	129.42488	G	1122.60143	561.80435	374.87199	10
3	501.28703	251.14715	167.76719	D	1065.57997	533.29362	355.86484	9
4	615.32996	308.16862	205.78150	N	950.55302	475.78015	317.52253	8
5	714.39837	357.70282	238.80431	V	836.51010	418.75869	279.50822	7
6	827.48243	414.24486	276.49900	L	737.44168	369.22448	246.48541	6
7	964.54135	482.77431	322.18530	H	624.35762	312.68245	208.79072	5
8	1077.62541	539.31634	359.87999	L	487.29871	244.15299	163.10442	4
9	1148.66252	574.83490	383.55903	A	374.21464	187.61096	125.40973	3
10	1276.72110	638.86419	426.24522	Q	303.17753	152.09240	101.73069	2
11				R	175.11895	88.06311	59.04450	1

FL0008626.raw #39513 RT: 77.4590 min
ITMS, 484.6158@cid35.00, z=+3, Mono m/z=484.28149 Da, MH+=1450.82993 Da, Match Tol.=0.6 Da

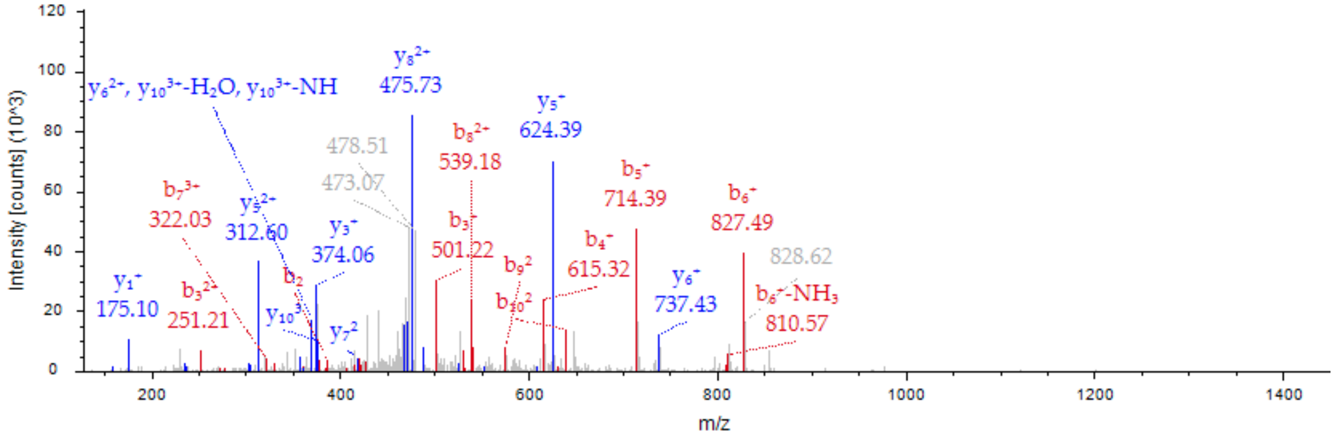

Xcorr: 3.15

HLYICDFHK (Q9HAJ7)

#1	b⁺	b²⁺	b³⁺	b⁴⁺	Seq.	y⁺	y²⁺	y³⁺	y⁴⁺
1	367.22912	184.11820	123.08122	92.56274	H-TMT6plex				
2	480.31318	240.66023	160.77591	120.83375	L	1324.69211	662.84969	442.23555	331.92848
3	643.37651	322.19189	215.13036	161.59959	Y	1211.60805	606.30766	404.54087	303.65747
4	756.46058	378.73393	252.82504	189.87060	I	1048.54472	524.77600	350.18642	262.89164
5	916.49123	458.74925	306.16859	229.87826	C-Carbami...	935.46065	468.23396	312.49174	234.62062
6	1031.51817	516.26272	344.51091	258.63500	D	775.43000	388.21864	259.14819	194.61296
7	1178.58658	589.79693	393.53371	295.40210	F	660.40306	330.70517	220.80587	165.85622
8	1315.64549	658.32639	439.22002	329.66683	H	513.33465	257.17096	171.78307	129.08912
9					K-TMT6plex	376.27574	188.64151	126.09676	94.82439

FL0008644.raw #42592 RT: 81.7965 min
ITMS, 423.4840@cid35.00, z=+4, Mono m/z=423.48401 Da, MH+=1690.91421 Da, Match Tol.=0.6 Da

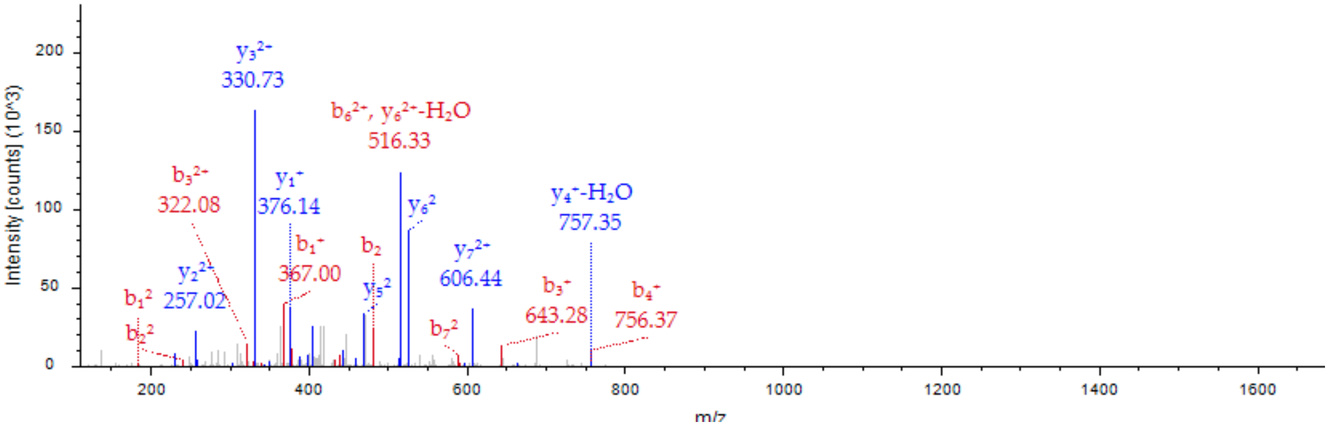

Xcorr: 3.14

GGPEDGGLGALR (O95977)

#1	b⁺	b²⁺	Seq.	y⁺	y²⁺	#2
1	287.19167	144.09947	G-TMT6plex			12
2	344.21314	172.61021	G	1041.53235	521.26981	11
3	441.26590	221.13659	P	984.51088	492.75908	10
4	570.30849	285.65788	E	887.45812	444.23270	9
5	685.33544	343.17136	D	758.41553	379.71140	8
6	742.35690	371.68209	G	643.38858	322.19793	7
7	799.37836	400.19282	G	586.36712	293.68720	6
8	912.46243	456.73485	L	529.34566	265.17647	5
9	969.48389	485.24558	G	416.26159	208.63444	4
10	1040.52100	520.76414	A	359.24013	180.12370	3
11	1153.60507	577.30617	L	288.20302	144.60515	2
12			R	175.11895	88.06311	1

FL0008616.raw #36354 RT: 71.9006 min
ITMS, 664.3618@cid35.00, z=+2, Mono m/z=664.36182 Da, MH+=1327.71636 Da, Match Tol.=0.6 Da

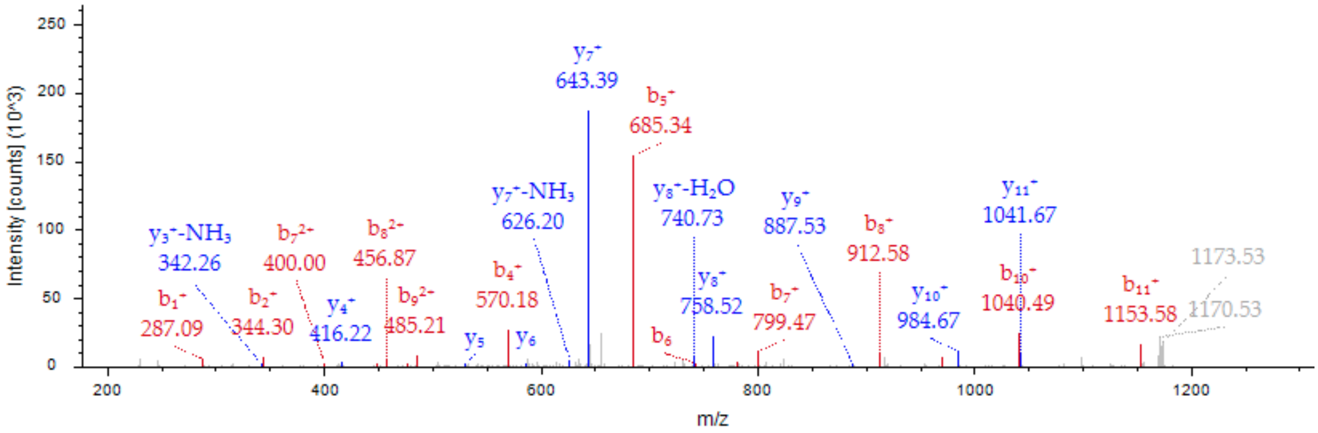

HQIHDADVR
(Q9UIW2)

Xcorr: 1.97

#1	b⁺	b²⁺	b³⁺	Seq.	y⁺	y²⁺	y³⁺	#2
1	367.22912	184.11820	123.08122	H-TMT6plex				9
2	495.28770	248.14749	165.76742	Q	953.47992	477.24360	318.49816	8
3	608.37176	304.68952	203.46211	I	825.42134	413.21431	275.81196	7
4	745.43067	373.21898	249.14841	H	712.33728	356.67228	238.11728	6
5	860.45762	430.73245	287.49072	D	575.27837	288.14282	192.43097	5
6	931.49473	466.25100	311.16976	A	460.25142	230.62935	154.08866	4
7	1046.52167	523.76447	349.51208	D	389.21431	195.11079	130.40962	3
8	1145.59009	573.29868	382.53488	V	274.18737	137.59732	92.06731	2
9				R	175.11895	88.06311	59.04450	1

FL0008644.raw #7583 RT: 19.5958 min
ITMS, 440.5717@cid35.00, z=+3, Mono m/z=440.57166 Da, MH+=1319.70041 Da, Match Tol.=0.6 Da

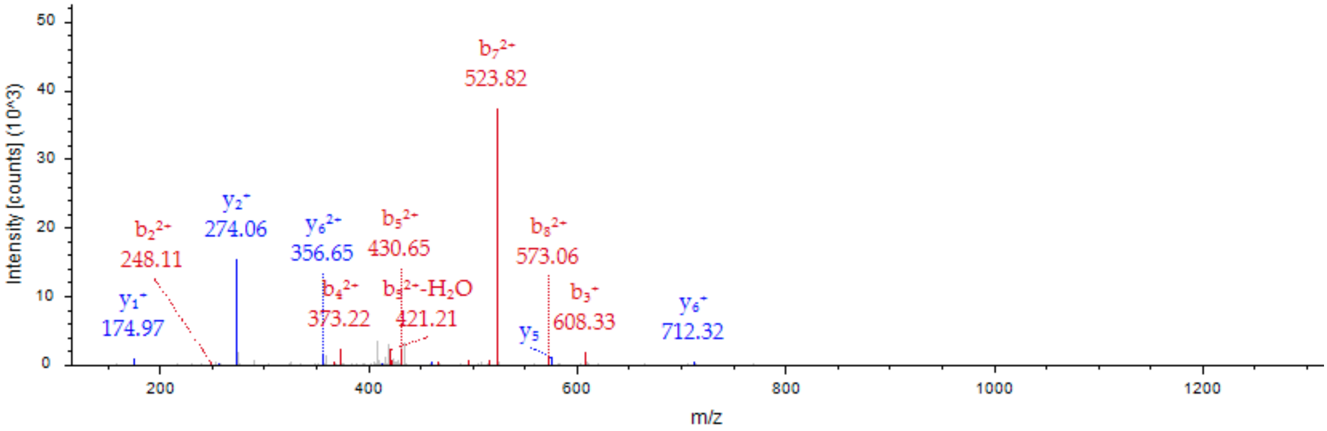

ETAAEEESR
(Q7Z5W3)

Xcorr: 2.34

#1	b⁺	b²⁺	Seq.	y⁺	y²⁺	#2
1	359.21280	180.11004	E-TMT6plex			9
2	460.26048	230.63388	T	892.40067	446.70397	8
3	531.29759	266.15244	A	791.35299	396.18013	7
4	602.33471	301.67099	A	720.31587	360.66158	6
5	731.37730	366.19229	E	649.27876	325.14302	5
6	860.41989	430.71359	E	520.23617	260.62172	4
7	989.46249	495.23488	E	391.19357	196.10043	3
8	1076.49451	538.75090	S	262.15098	131.57913	2
9			R	175.11895	88.06311	1

FL0008606.raw #12468 RT: 31.4594 min
ITMS, 625.8063@cid35.00, z=+2, Mono m/z=625.80627 Da, MH+=1250.60527 Da, Match Tol.=0.6 Da

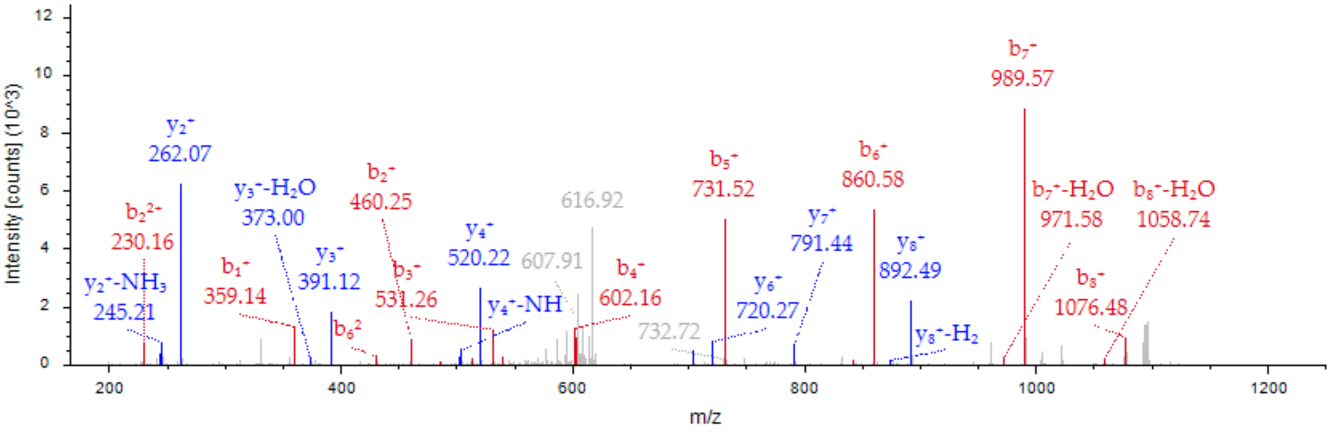

KEQQQSEANELR
(Q5VSY0)

Xcorr: 3.06

#1	b+	b2+	b3+	Seq.	y+	y2+	y3+	#2
1	587.42810	294.21769	196.48089	K-TMT6ple...				12
2	716.47070	358.73899	239.49508	E	1331.61860	666.31294	444.54438	11
3	844.52927	422.76828	282.18128	Q	1202.57600	601.79164	401.53019	10
4	972.58785	486.79756	324.86747	Q	1074.51743	537.76235	358.84399	9
5	1100.64643	550.82685	367.55366	Q	946.45885	473.73306	316.15780	8
6	1187.67846	594.34287	396.56434	S	818.40027	409.70377	273.47161	7
7	1316.72105	658.86416	439.57853	E	731.36824	366.18776	244.46093	6
8	1387.75816	694.38272	463.25757	A	602.32565	301.66646	201.44673	5
9	1501.80109	751.40418	501.27188	N	531.28854	266.14791	177.76770	4
10	1630.84368	815.92548	544.28608	E	417.24561	209.12644	139.75339	3
11	1743.92775	872.46751	581.98077	L	288.20302	144.60515	96.73919	2
12				R	175.11895	88.06311	59.04450	1

FL0008626.raw #19615 RT: 43.7738 min
ITMS, 640.0178@cid35.00, z=+3, Mono m/z=640.01782 Da, MH+=1918.03891 Da, Match Tol.=0.6 Da

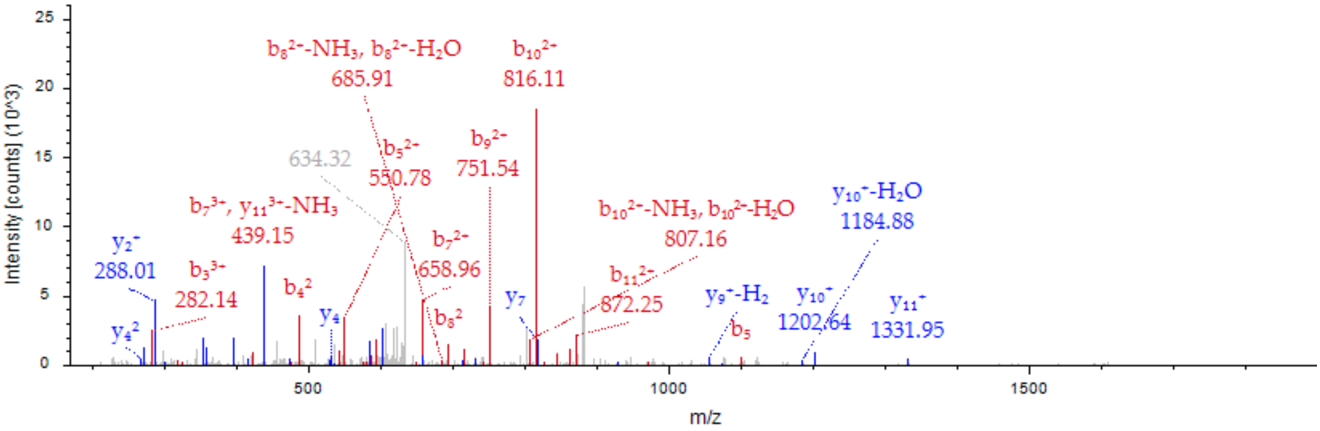

SFNSDDFDYEELK (Q8IVV7)

Xcorr: 3.97

#1	b⁺	b²⁺	Seq.	y⁺	y²⁺	#2
1	317.20224	159.10476	S-TMT6plex			13
2	464.27065	232.63896	F	1750.80093	875.90410	12
3	578.31358	289.66043	N	1603.73251	802.36989	11
4	665.34561	333.17644	S	1489.68959	745.34843	10
5	780.37255	390.68991	D	1402.65756	701.83242	9
6	895.39949	448.20338	D	1287.63061	644.31895	8
7	1042.46791	521.73759	F	1172.60367	586.80547	7
8	1157.49485	579.25106	D	1025.53526	513.27127	6
9	1320.55818	660.78273	Y	910.50831	455.75780	5
10	1449.60077	725.30402	E	747.44499	374.22613	4
11	1578.64336	789.82532	E	618.40239	309.70483	3
12	1691.72743	846.36735	L	489.35980	245.18354	2
13			K-TMT6plex	376.27574	188.64151	1

FL0008588.raw #63727 RT: 118.0956 min
ITMS, 1034.4998@cid35.00, z=+2, Mono m/z=1033.99817 Da, MH+=2066.98906 Da, Match Tol.=0.6 Da

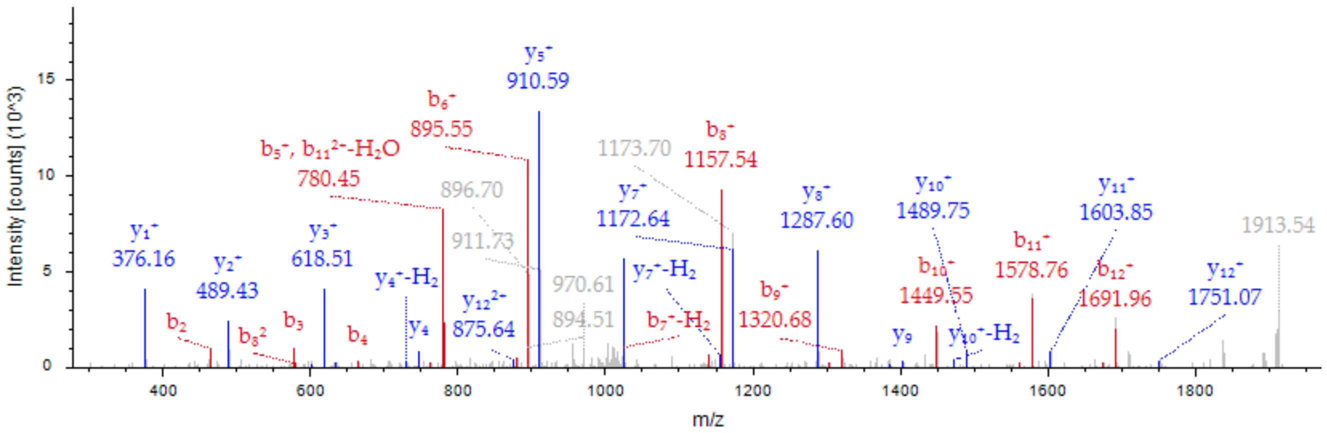

QTEFAPETGK
(Q8N8R7)

Xcorr: 2.66

#1	b⁺	b²⁺	Seq.	y⁺	y²⁺	#2
1	358.22879	179.61803	Q-TMT6plex			10
2	459.27646	230.14187	T	1208.63603	604.82166	9
3	588.31906	294.66317	E	1107.58836	554.29782	8
4	735.38747	368.19737	F	978.54576	489.77652	7
5	806.42459	403.71593	A	831.47735	416.24231	6
6	903.47735	452.24231	P	760.44024	380.72376	5
7	1032.51994	516.76361	E	663.38747	332.19737	4
8	1133.56762	567.28745	T	534.34488	267.67608	3
9	1190.58908	595.79818	G	433.29720	217.15224	2
10			K-TMT6plex	376.27574	188.64151	1

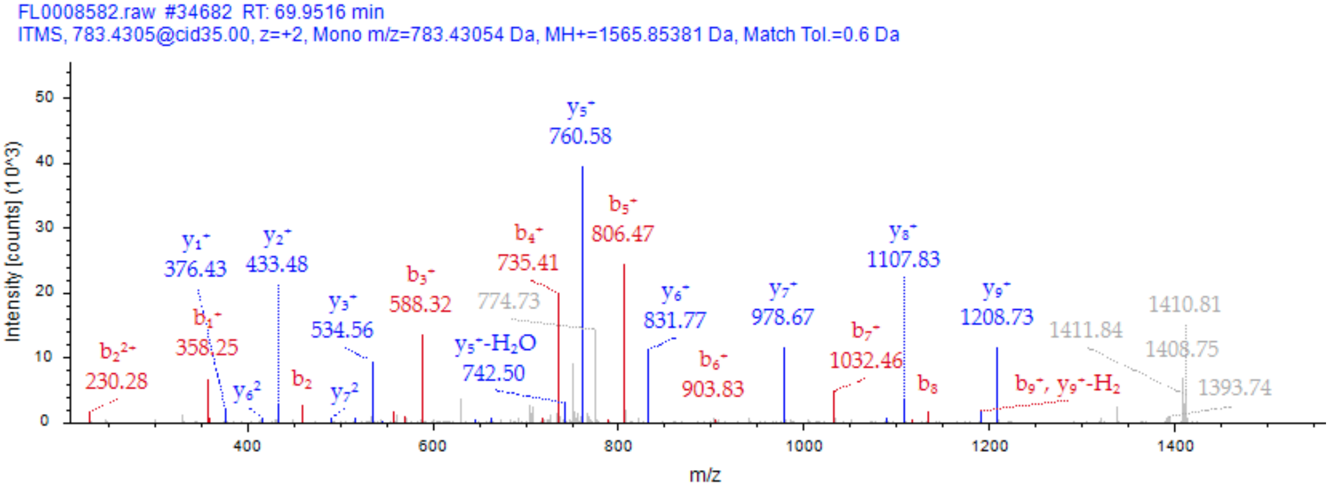

KETQILLLDLLPDR
(Q6DHV7)

Xcorr: 1.87

#1	b+	b2+	b3+	Seq.	y+	y2+	y3+	#2
1	587.42810	294.21769	196.48089	K-TMT6ple...				13
2	716.47070	358.73899	239.49508	E	1425.79477	713.40102	475.93644	12
3	817.51838	409.26283	273.17764	T	1296.75218	648.87973	432.92224	11
4	945.57695	473.29211	315.86384	Q	1195.70450	598.35589	399.23968	10
5	1058.66102	529.83415	353.55852	I	1067.64592	534.32660	356.55349	9
6	1171.74508	586.37618	391.25321	L	954.56186	477.78457	318.85880	8
7	1284.82914	642.91821	428.94790	L	841.47779	421.24254	281.16412	7
8	1399.85609	700.43168	467.29021	D	728.39373	364.70050	243.46943	6
9	1512.94015	756.97371	504.98490	L	613.36679	307.18703	205.12711	5
10	1626.02422	813.51575	542.67959	L	500.28272	250.64500	167.43243	4
11	1723.07698	862.04213	575.03051	P	387.19866	194.10297	129.73774	3
12	1838.10392	919.55560	613.37283	D	290.14590	145.57659	97.38682	2
13				R	175.11895	88.06311	59.04450	1

FL0008638.raw #74949 RT: 137.4886 min
ITMS, 671.7446@cid35.00, z=+3, Mono m/z=671.40955 Da, MH+=2012.21408 Da, Match Tol.=0.6 Da

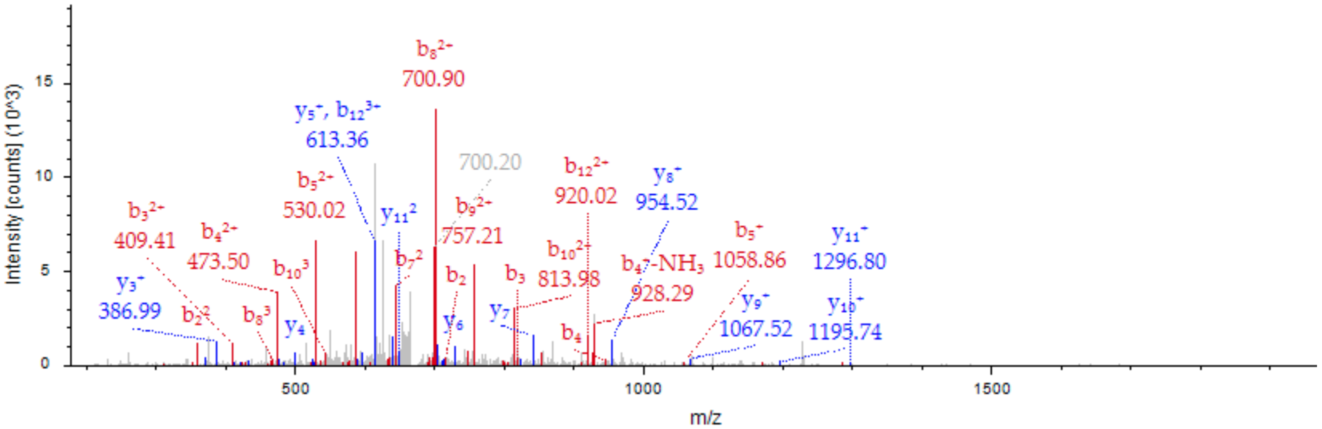

ICIDPWLLDHR
(Q8NC42)

Xcorr: 3.39

#1	b⁺	b²⁺	b³⁺	Seq.	y⁺	y²⁺	y³⁺	#2
1	343.25427	172.13077	115.08961	I-TMT6plex				11
2	503.28492	252.14610	168.43316	C-Carbami...	1324.64667	662.82697	442.22041	10
3	616.36898	308.68813	206.12785	I	1164.61602	582.81165	388.87686	9
4	731.39593	366.20160	244.47016	D	1051.53195	526.26962	351.18217	8
5	828.44869	414.72798	276.82108	P	936.50501	468.75614	312.83985	7
6	1014.52800	507.76764	338.84752	W	839.45225	420.22976	280.48893	6
7	1127.61207	564.30967	376.54221	L	653.37293	327.19011	218.46250	5
8	1240.69613	620.85170	414.23690	L	540.28887	270.64807	180.76781	4
9	1355.72308	678.36518	452.57921	D	427.20481	214.10604	143.07312	3
10	1492.78199	746.89463	498.26551	H	312.17786	156.59257	104.73081	2
11				R	175.11895	88.06311	59.04450	1

FL0008642.raw #67402 RT: 122.7091 min
ITMS, 556.6365@cid35.00, z=+3, Mono m/z=556.30182 Da, MH+=1666.89090 Da, Match Tol.=0.6 Da

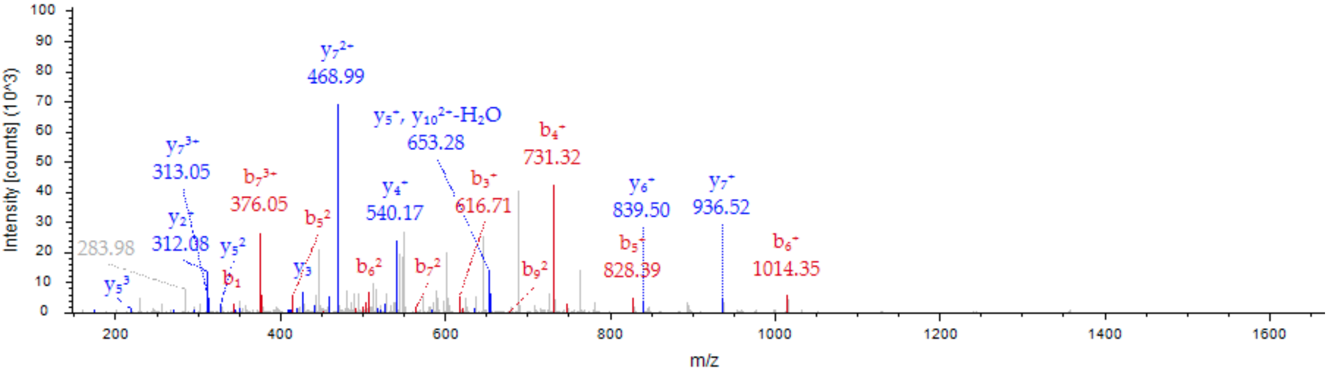

Supplement: Supplemental Dataset S2 [file mmc3.pdf]
