# Supplementary material for: Tuning DO:DM Ratios Modulates MHC Class II Immunopeptidomes
Source: Mol Cell Proteomics. 2022 Jan 25;21(3):100204. doi: 10.1016/j.mcpro.2022.100204 (PMC10329146; doi:10.1016/j.mcpro.2022.100204)
Supplement: Supplemental Table S1 [file mmc5.pdf]

Sample	Unique peptide IDs	Unique peptide IDs (quant both bioreps.)	Cores	Cores for NetMHC
T2DR4 (biorep1)<br>T2DR4 (biorep2)	2768<br>2873	1380	476	475
T2DR4DM (biorep1)<br>T2DR4DM (biorep2)	3119<br>3349	1943	527	525
T2DR4DMDO(+) (biorep1)<br>T2DR4DMDO(+) (biorep2)	3514<br>3594	2187	607	605
T2DR4DMDO(++) (biorep1)<br>T2DR4DMDO(++) (biorep2)	3337<br>3125	1839	538	534
T2DR4DMDO(+++) (biorep1)<br>T2DR4DMDO(+++) (biorep2)	3423<br>3529	1849	658	656
T2DR4DMDO-Knockout (biorep1)<br>T2DR4DMDO-Knockout (biorep2)	1502<br>2727	799	243	242
Total # of unique peptides in dataset	10587	4528	1206*	1206*

*Total core epitopes when all cell lines analyzed together
